# The lexical categorization model: A computational model of left ventral occipito-temporal cortex activation in visual word recognition

**DOI:** 10.1371/journal.pcbi.1009995

**Published:** 2022-06-09

**Authors:** Benjamin Gagl, Fabio Richlan, Philipp Ludersdorfer, Jona Sassenhagen, Susanne Eisenhauer, Klara Gregorova, Christian J. Fiebach

**Affiliations:** 1 Department of Psychology, Goethe University Frankfurt, Frankfurt am Main, Germany; 2 Center for Individual Development and Adaptive Education of Children at Risk (IDeA), Frankfurt am Main, Germany; 3 Department of Linguistics, University of Vienna, Vienna, Austria; 4 Centre for Cognitive Neuroscience, Paris-Lodron-University of Salzburg, Salzburg, Austria; 5 Wellcome Trust Centre for Neuroimaging, Institute of Neurology, University College London, London, United Kingdom; 6 Universitätsklinikum Würzburg, Universität Würzburg, Würzburg, Germany; 7 Brain Imaging Center, Goethe University Frankfurt, Frankfurt am Main, Germany; UC Irvine: University of California Irvine, UNITED STATES

## Abstract

To characterize the functional role of the left-ventral occipito-temporal cortex (lvOT) during reading in a quantitatively explicit and testable manner, we propose the *lexical categorization model* (LCM). The LCM assumes that lvOT optimizes linguistic processing by allowing fast meaning access when words are familiar and filtering out orthographic strings without meaning. The LCM successfully simulates benchmark results from functional brain imaging described in the literature. In a second evaluation, we empirically demonstrate that quantitative LCM simulations predict lvOT activation better than alternative models across three functional magnetic resonance imaging studies. We found that word-likeness, assumed as input into a lexical categorization process, is represented posteriorly to lvOT, whereas a dichotomous word/non-word output of the LCM could be localized to the downstream frontal brain regions. Finally, training the process of lexical categorization resulted in more efficient reading. In sum, we propose that word recognition in the ventral visual stream involves word-likeness extraction followed by lexical categorization before one can access word meaning.

## Author summary

Visual word recognition is a critical process for reading and relies on the human brain’s left ventral occipito-temporal (lvOT) regions. However, the lvOTs specific function in visual word recognition is not yet clear. We propose that these occipito-temporal brain systems are critical for lexical categorization, i.e., the process of determining whether an orthographic percept is a known word or not, so that further lexical and semantic processing can be restricted to those percepts that are part of our "mental lexicon". We demonstrate that a computational model implementing this process, the *lexical categorization model*, can explain seemingly contradictory benchmark results from the published literature. We further use functional magnetic resonance imaging to show that the lexical categorization model successfully predicts brain activation in the left ventral occipito-temporal cortex elicited during a word recognition task. It does so better than alternative models proposed so far. Finally, we provide causal evidence supporting this model by empirically demonstrating that training the process of lexical categorization improves reading performance.

## Introduction

Reading is a crucial cultural achievement, and efficient recognition of written words is at its core. Investigations of brain activations in response to visually presented words highlighted the role of a region in the left ventral occipito-temporal cortex (lvOT). This region is often also referred to as the visual word form area [1]. The presentation of letter strings (i.e., letter combinations that can be words or non-words) reliably activates this region [2–4], its structure and function is compromised in developmental reading disorders [5,6], lesions of this brain region result in severe reading deficits [7], and electrical stimulation of this brain region impairs word recognition [3,8]. However, there is no agreed-upon mechanistic understanding of the specific processes that lvOT contributes to visual word recognition [9]. Here, we propose a simple computational model of the lvOT function during reading. The *lexical categorization model* (LCM) integrates insights from neurocognitive and psycholinguistic research to explicitly model the profile of the lvOT responses to different types of orthographic stimuli.

The lvOT is part of the ventral visual processing stream [10]. It was proposed that lvOT receives converging bottom-up visual input from both hemispheres and that it processes abstract representations of recurring letter sequences–including sublexical units and small words [1,11]. This proposal is in part based on the finding that lvOT is sensitive to word-*similarity* [12,13], in the sense of decreasing lvOT activation (measured with functional magnetic resonance imaging; fMRI) with decreasing word-*similarity* of non-words. Specifically, high activation is observed when processing non-words containing letter sequences that frequently occur in real words (e.g., ‘ous’ in *mousa*). In contrast, non-words containing illegal letter combinations (e.g., *mkzsq*) result in low lvOT activation [12,13]. Seemingly contradictory, it was reported that more *familiar* (i.e., more frequently occurring) words elicit less lvOT activation as compared to rarely occurring (low frequent) words [14]. In sum, empirical data indicate that while word-*similarity* (in the sense of sub-lexical orthographic similarity) increases lvOT activation, word-*familiarity* (in the sense of word frequency) decreases lvOT activation. We interpret this counter-intuitive set of results as suggesting that lvOT responds in a non-linear fashion to the ‘word-likeness’ of orthographic strings, i.e., showing highest activity for words of intermediate word-likeness (e.g., words with low word-familiarity and non-words with high word-similarity) and least activity both for highly familiar, frequent words as well as for orthographically illegal and rarely co-occurring (‘word-un-like’) strings of letters (see also [4,15]).

The non-linear response profile of lvOT to different types of orthographic stimuli resembles the relationship between word-likeness and behavioral performance in word recognition tasks. Using a lexical decision task (categorical word/non-word decisions), Balota and Chumbley [16] observed that lexical decisions for letter strings with intermediate levels of word-likeness were more difficult (e.g., produced higher error rates) than decisions to very familiar words or very ‘word-un-like’ non-words (see also [17,18]). Based on these results, these authors proposed that letter strings at both extreme ends of the word-likeness continuum (i.e., highly familiar or highly word-un-like) can be classified quickly as words vs. non-words based on word-likeness information. In contrast, at intermediate levels of word-likeness, e.g., for rarely occurring words, words of a foreign language, or–as often used in psychological experiments–orthographically legal but meaningless pseudowords, uncertainty exists concerning the lexical nature of the letter strings. Balota and Chumbley [16] assume that this ambiguity is reduced by further analytic processing, e.g., based on processing sub-lexical units (e.g., letter) or word spellings.

We propose that lvOT implements, as core computation, an analogous process, i.e., a dichotomous lexical/non-lexical categorization of perceived orthographic strings informed by their word-likeness. lvOT thereby allows for fast access to subsequent linguistic processing stages for highly familiar words and, at the same time, filters out unknown or linguistically irrelevant percepts. Lexical categorization, thus, effectively functions as a gate that can save energy resources by preventing attempts to interpret unknown linguistic percepts. Thus, the proposed neurocognitive process of lexical categorization mirrors the behavioral pattern observed in lexical decision tasks [16], and the non-linear patterns of both lvOT activity and behavior are reflections of the level of uncertainty associated with the categorization process. The LCM proposal is consistent with one of the core computational functions of the ventral visual stream, i.e., categorizing percepts into different categories of objects based on a hierarchy of spatially different cortical processing stages [19]. For example, animate vs. inanimate objects activate separable ventral stream regions, while spatially dissociable sub-regions represent different lower-level features, such as faces or eyes for the animate subcategory. This object categorizing architecture is also known to categorize words vs. non-orthographic objects efficiently [8,20,21], and activation in these regions correlates with lexical decision performance [22]. Within the framework of hierarchically organized ventral stream processing, we thus assume that a categorical distinction between known and unknown orthographic percepts is computed after a lower-level categorization of sensory information into orthographic vs. non-orthographic input has taken place. More domain-general computational work has characterized the functional role of category-specific response patterns in vOT (e.g., [23]). In contrast, we here aim to more specifically model the effects of orthographic and lexical stimulus properties on word-selective cortex. Whereas previous work indicates that the former categorization process (e.g., words vs. faces) is based on visually distinct features, the lexical categorization process postulated here is proposed to rely on category-specific (e.g., orthographic, lexical) information.

To characterize the lvOT’s role for word recognition in a quantitatively explicit and testable manner, we implemented this hypothesis in a simple computational model: the *lexical categorization model* (LCM). The LCM predicts lexical categorization difficulty based on the word-likeness of the input letter string. Given its computational implementation, the LCM allows for model simulations and direct model evaluations by correlating simulated with empirical data. This proposal contrasts with previous conceptions of lvOT functioning in visual word recognition, which have been purely verbal-descriptive. Also, previous proposals divide into models that either assume linear or non-linear response profiles. Linear models include the *local combination detector* model [11,12] or the lexicon model [14,24,25]. The *local combination detector* model suggests that lvOT activation reflects a perceived letter string’s overlap with stored representations. The *lexicon* model assumes a word-frequency-based whole word lexicon search process (that lasts longer and thus elicits more activity in low-frequency words and non-words. However, these models cannot account for the non-linear response profile of lvOT discussed above. Non-linear models include the *engagement and effort* (E&E) model [4] and the *interactive account* (IA) model [15]. The E&E model assumes that the lvOT activation reflects the combination of the engagement and effort related to an orthographic stimulus. Engagement reflects that an illegal letter string (i.e., highly unfamiliar letter combinations) is less likely to be processed by a word recognition area when compared to a known word, so that brain activation is expected to be lower for the illegal string. Effort, in contrast, reflects that lvOT activation is greater for less often processed words than for frequently encountered words [4]. The IA model assumes orthographic processing based on the principles of predictive coding [26] based orthographic processing, such that lvOT activation reflects the error associated with internally generated predictions about the upcoming word [15]. The non-linearity of the IA model comes from the assumption that consonant strings do activate lvOT to a lesser extent since for these letter strings, few top-down predictions can be formed (e.g., in the context of an experimental task), which results in a low prediction error. In contrast, rare words are less often expected and thus often violate current predictions, so that the prediction error (and consequently the activation of lvOT), is high. Frequent words, on the other hand, can be predicted better so that both the prediction error and lvOT activation are lower for high- than for low-frequent words. None of these models of lvOT activation during word recognition have been implemented yet, so that explicit model comparisons are missing. Also, these alternative non-linear models can likely capture the response profile of lvOT activation to some extent but are conceptually harder to integrate into the predominant categorization-based accounts present for object recognition (e.g., [19] and see above) than the LCM.

Here, we evaluate the LCM against ad hoc implementations of the four alternative models introduced above and against a widely acknowledged, implemented cognitive model of visual word recognition, the *Dual Route* model [27] (which is in part a connectionist model). These evaluations focus primarily on comparing simulated vs. empirically observed lvOT responses to different stimulus characteristics–by generating simulated brain activations of observed activation patterns from different models (model-based fMRI analysis; see, e.g., [28]). First, models are compared based on their ability to simulate nine benchmark effects reported in the literature (Evaluation based on simulations). Second, we report empirical evaluations based on new fMRI studies that localize a neural correlate of the lexical categorization computation. After that, we compare models based on how well their simulations fit to the observed patterns of lvOT activatio (fMRI-based Evaluation). Finally, we investigate if training the core computation assumed in the LCM, i.e., the process of lexical categorization, increases reading efficiency (Training-based Evaluation).

### Implementation of the lexical categorization model

**Fig 1:**
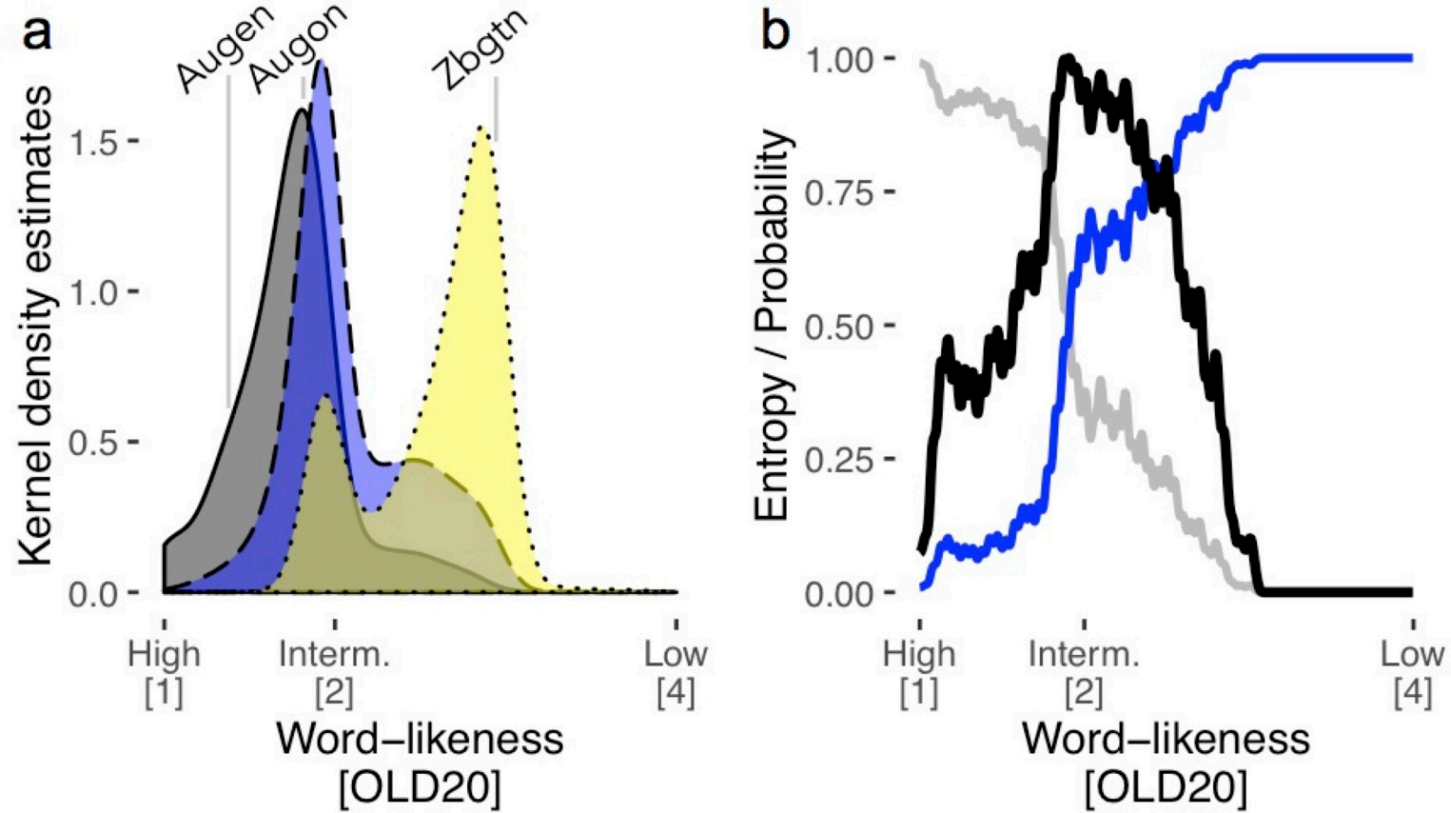
Description of the lexical categorization model (LCM). (a) Word-likeness distributions (kernel density estimates), based on the orthographic Levenshtein distance (OLD20 [29]) of words (gray), pseudowords (blue), and consonant strings (yellow) including an example for each category. (b) Probability that a letter string given its OLD20 value is a word (gray line) or a non-word (i.e., either pseudoword or consonant string; blue line). The black line represents the estimated entropy (see Eq 1, *Methods* section, for more details), which combines the probabilities of being a word or non-word across all possible OLD20 values. The LCM’s central hypothesis is that word-sensitive lvOT activation reflects this entropy function across all possible levels of word-likeness, effectively representing the difficulty of the lexical categorization process postulated for the lvOT. In an attempt to assess the internal stability of the LCM, we estimated LCM simulations based on only subsets of the words, pseudowords, and consonant strings used for the results presented here. In doing so, we found that only about 8% of the lexical items are needed to achieve stable LCM simulations (see S6 Fig).

To implement the LCM, we first derived the word-likeness distributions of a large set of orthographic strings: We estimated word-likeness (using an established measure from word recognition research, i.e., the orthographic Levenshtein distance, OLD20 [29]) of all German words with a length of five letters and an initial uppercase letter (i.e., nouns and noun-forms, e.g., plural form; N = 3,110; example: *Augen/eyes*), as well as for the same numbers of pseudowords (e.g., *Augon*) and consonant strings (e.g., *Zbgtn*) derived from these words. We visualized these distributions in Fig 1A. Expectedly, real words are characterized by high word-likeness, as shown by a relatively focused distribution between OLD20 values of 1 and 2 (gray distribution in Fig 1A). Technically, this indicates that they share many orthographic features with their twenty nearest orthographic neighbors. In contrast, the yellow distribution in Fig 1A, with its peak around an OLD20 of 3, demonstrates that consonant strings differ strongly from orthographically legal, existing words. For these two stimulus types with peaks at the extreme ends of the word-likeness distribution, word/non-word categorizations can be achieved with high degrees of certainty based on a word-likeness estimate. This certainty is reflected in the probabilities of a string being a word or a non-word given its word-likeness (gray and blue line, respectively, in Fig 1B). In contrast, the word-likeness (OLD20) distributions of words and pseudowords overlap strongly in the intermediate-likeness range (Fig 1A; consistent with previous literature [16]). Thus, for stimuli with intermediate word-likeness levels, lexical status is ambiguous, so that the word/non-word categorization cannot be based on word-likeness alone (probability of being a word or non-word is ~.5; Fig 1B). As a consequence, correct lexical categorization can only be achieved based on additional evidence [16], for example by comparing the presented letter string to more explicit representations that allow the correct spelling when writing words. To summarize, these considerations suggest that computing a lexical (word/non-word) categorization from an estimate of word-likeness is hard when word-likeness distributions of words and non-words overlap (as is the case for orthographically legal pseudowords) and easy when they do not (as is the case for frequent words as opposed to pure consonant strings). As Fig 1B (black line) indicates, this pattern of lexical categorization difficulty is very well captured by the information-theoretical concept of entropy, i.e., in our case, how much additional information is needed for the categorization (see *Methods* for details of the implementation). Thus, the basic assumption of the LCM is that the non-linear response profile of word-sensitive areas of lvOT should be reflected in the entropy of the lexical categorization, i.e., how difficult is it to categorize a letter string correctly.

## Results

### Evaluation based on simulations: LCM simulations

As a first test, we used the LCM, as well as the implementations of four alternative neurocognitive models described above [1,4,15,30] and one purely cognitive model, the Dual-Route model [27], to assess how well these models reproduce lvOT activation reported in the literature for the most frequently published contrasts between different types of visually presented letter strings. We implemented the LCM simulations by transforming the word-likeness of each letter string into a lexical categorization uncertainty via the entropy function depicted in Fig 1B and described in more detail in the *Methods* section.

**Fig 2:**
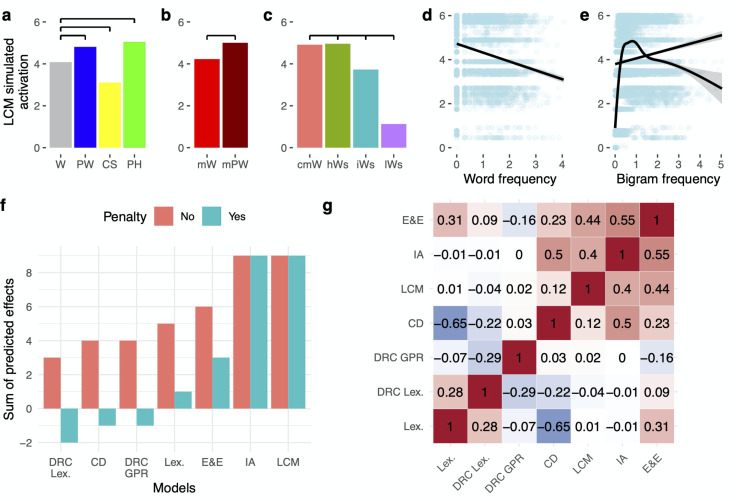
Evaluation based on simulations: LCM and model comparisons based on simulations of lvOT benchmark effects from the fMRI literature. Model comparisons involve the lexicon model, local combination detector model (CD), effort and engagement model (E&E), the interactive account model (IA; for implementations and detailed simulations see *S1 Text*), and the cognitive Dual Route model, specifically its orthographic lexicon (DRC Lex.) and its grapheme to phoneme conversion route (DRC GPR; for detailed simulations see *S1 Fig*). Simulated lvOT activation (in arbitrary units: min = 0; max = 6) for all groups of letter strings is presented using bar graphs depicting their respective mean activation. In addition, horizontal black bars indicate significant differences of the simulation results between letter string categories, as derived from linear models (Bonferroni corrected). LCM simulated lvOT activation is presented, from left to right, (a) for words (W), pseudowords (PW), consonant strings (CS), pseudohomophones (PH), (b) words and pseudowords matched on number of syllables, number of Coltheart’s orthographic neighbors, frequency of the highest frequency neighbor, initial bigram frequency, final bigram frequency, and summated bigram frequency (mW, mPW), and (c) the word similarity effect comparing words (cmW: comparative matched words) to non-words with high word similarity (matched on quadrigram frequency; hWS), to non-words with intermediate word similarity (matched on bigram frequency; iWS), and, to non-words with low word similarity (lWS). In addition, (d) the effect of log. transformed word frequency (for all words and pseudowords as tested in [14]) and (e) log. transformed bigram frequency are presented as scatter plots with a linear regression line. Note that for bigram frequency, also a non-linear regression line is shown. In (d) and (e), each dot represents one letter string, the more saturated the blue gets, the more letter strings are included. See text for more detailed description of the replicated benchmark effects including the specific stimulus sets used and references to the original studies. (f) Qualitative model comparisons showing the sum of correctly simulated stimulus differences (orange bars) and all correct minus all incorrectly simulated effects excluding null effects (cyan bars). The LCM and the IA were able to correctly simulate all contrasts. (g) Correlation matrix of all model parameters included in the model comparison, showing that simulations of the non-linear models, i.e., LCM, E&E, and IA, were substantially correlated (all r’s > .4).

Fig 2A–2E displays LCM simulations for different types of letter strings (see *Methods*), all of which successfully reproduce published fMRI-based lvOT activation results. In detail, this involves fMRI contrasts of pseudowords > words [4] (Fig 2A); words > consonant strings [2] (Fig 2A); pseudowords > words > consonant strings [31] (Fig 2A); pseudohomophones > words and pseudohomophones = pseudowords [24]; pseudowords > words matched on multiple lexical characteristics [32] (Fig 2B); the word similarity effect: low word similarity < intermediate word similarity < high word similarity = words [12] (Fig 2C); increasing lvOT activation with decreasing word frequency [14] including pseudowords (Fig 2D; note that when only words were used, the beta-weight in that study was reduced from -0.40 to -0.17; see also [33–35]); bigram frequency effect: increasing lvOT activation with increasing bigram frequency [13] (Fig 2E; both frequency measures had been log. transformed). Note that bigram frequency was also previously used for estimating word-likeness and is correlated to the OLD20 parameter (e.g., see Fig 2B in Ref. [18]). Therefore, we also visualize the non-linear bigram frequency effect in the LCM simulation (Fig 2E, dashed line), resolving the inconsistent findings concerning lvOT and bigram frequency previously reported, i.e., an increase in activation with bigram frequency in [13] as opposed to a decrease in [35].

When we simulate these contrasts with all five neurocognitive models and the German version of the Dual Route model, the different models can be compared based on the number of correctly simulated contrasts (Fig 2F and 2G; for details on the implementations and the results of the simulations of alternative models [1,4,14,15,27], see *S1 Text*). Note that for the Dual Route model [27], we conducted two such simulations, one reflecting the model-based activations of the orthographic lexicon, similar as assumed in the lexicon model [30], and one reflecting the activation of the grapheme-to-phoneme route, as suggested by [25] (reflecting letter-by-letter decoding). This differentiation allowed us to investigate the model’s two routes as independent hypotheses for the cognitive processes implemented in the word-sensitive lvOT (see S1 Fig for details). We also investigated the response times simulated by the Dual Route model. However, no difference between the response times and the Dual Route model’s orthographic lexicon simulation was found, so that this was not included separately into the model comparison.

Only the LCM (Fig 2A–2E) and the IA model (S1 Text) could simulate all nine contrasts of interest correctly (Fig 2F). The E&E model, also implementing a non-linear response profile, performs better (6 out of 9 contrasts) than the neurocognitive models assuming a linear response profile and the cognitive Dual Route model. This finding suggests that implementing a non-linear response profile is a critical feature of the lvOT response to letter strings. This conclusion receives further support when we penalize the model performance for incorrect simulations (minus one for each incorrectly simulated effect) and from the positive inter-correlations among LCM, IA, and E&E models (Fig 2G; all *r*’s > .4). Given these results, we focused our empirical fMRI-based model comparisons on the three best-performing models, i.e., LCM, IA, and E&E models.

### fMRI-based evaluation: Brain activity measured with functional MRI

To test whether the quantitative predictions of the LCM capture empirically measured BOLD activity in lvOT, we used data from three fMRI studies (cf. *Methods* for acquisition parameters and preprocessing). In this section, we will first focus on the LCM effect and model comparisons. After that, we will present two additional effects of interest, i.e., the correlation of brain activation and word-likeness (OLD20) and the regions with higher activation levels for words, compared to pseudowords.

#### fMRI Study 1

**Fig 3:**
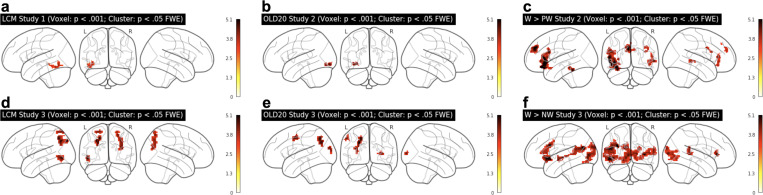
fMRI-based Evaluation of the LCM model: fMRI whole brain analyses. (a, b, d, e) Significant correlation results between BOLD signals and LCM simulations modeled as a single, continuous predictor in Study 1 (a) and Study 3 (d), and between BOLD activation and word-likeness represented by the OLD20 as a single, continuous predictor in Study 2 (b) and Study 3 (e). (c, f) Significantly increased BOLD signals for words as compared to non-words in Study 2 (c) and Study 3 (f). For OLD20 results and word > non-word contrasts of Study 1, see S2 Fig). Thresholds for all whole brain analyses: voxel level: *p* < .001 uncorrected (cluster-forming); cluster level: *p* < .05 family-wise error corrected. No other regions than those displayed were significant.

**Fig 4:**
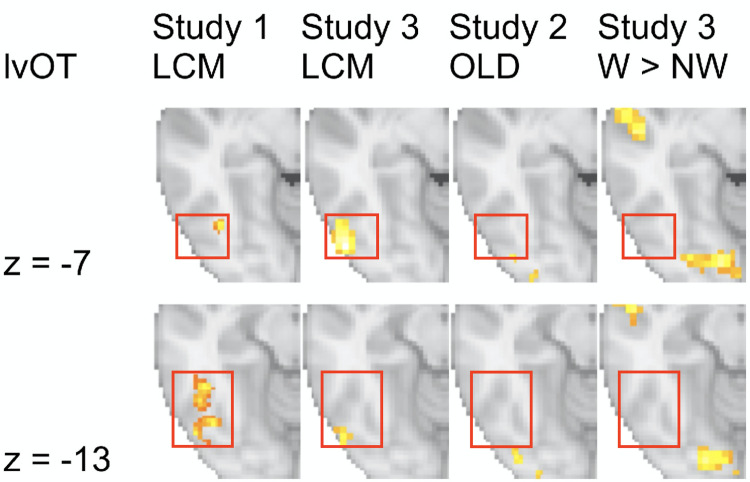
Comparison of BOLD activation across studies and contrasts. Significant LCM activation clusters in the lvOT across studies show some overlap, but no overlap to word-likeness and lexicality contrasts. Thresholds for all whole brain analyses: voxel level: *p* < .001 uncorrected (cluster-forming); cluster level: *p* < .05 family-wise error corrected.

Participants (N = 15 viewed a set of letter strings covering a wide range of word-likeness (i.e., words, pseudowords, and consonant strings; each five letters length), and strings of scrambled letters as a low-level perceptual control condition in a blocked design with 6 blocks of 16 items for each of the four conditions, presented in a random sequence (see *Methods* for details). Participants pressed a button whenever they detected a target stimulus (‘#####’). Control stimuli with scrambled letters were generated by randomly exchanging 90% of the pixels of the monochrome images of the word condition that were used for stimulus presentation, to obtain a list of maximally unfamiliar stimuli with identical physical input as the word condition. The analysis of BOLD activation was exclusively based on the lexical categorization difficulty as simulated by the LCM. In detail, we used item-specific LCM simulations as a continuous predictor for the fMRI data without explicitly accounting for condition differences and treated the control condition as having a very low word-likeness so that the LCM simulation resulted in a value of 0 (see *Methods* for details). We reasoned that if the proposed categorization process is indeed implemented in lvOT, quantitative LCM simulations should predict activation in this area. Our results strongly support this prediction: Across the entire brain volume, only voxels in the lvOT show a positive relationship between LCM simulations and BOLD response (Fig 3A; see also Fig 4, left-most column, and Table 1). Thus, the higher the categorization uncertainty expressed in the entropy derived from the word-likeness distributions from the lexicon, the higher the BOLD response in the identified lvOT voxels. The location of the detected activation cluster is consistent with previous functional localizations reported for the visual word form area (including published MNI peak coordinates such as -48, -56, -16 [12]).

**Table 1:** Significant activation clusters from the fMRI evaluation with respective anatomical labels (most likely regions from the Juelich and Harvard-Oxford atlases including % overlap), cluster size (in mm^3^), and peak voxel coordinates (MNI space).

Cluster	x	y	z	Mean T	Max T	Volume [mm^3^]	Juelich labels	Harvard-Oxford labels
Experiment 1: LCM effect								
1	-40	-64	-12	3.1	4.2	1760	5.45% Visual cortex V5 L	41.36% Left Inferior Temporal Gyrus temporooccipital part; 23.18% Left Lateral Occipital Cortex inferior division; 14.55% Left Occipital Fusiform Gyrus; 12.73% Left Temporal Occipital Fusiform Cortex; 6.36% Left Inferior Temporal Gyrus posterior division
Experiment 3: LCM effect								
1	24	-69	54	3.6	4.5	4158	29.22% Inferior parietal lobule PGp R; 28.57% Superior parietal lobule 7P R; 8.44% Superior parietal lobule 7A R	100.00% Right Lateral Occipital Cortex superior division
2	-30	-87	39	3.8	5.0	3159	35.04% Superior parietal lobule 7A L; 23.93% Inferior parietal lobule PGp L; 17.09% Anterior intra-parietal sulcus hIP3 L; 8.55% Superior parietal lobule 7P L; 5.13% Anterior intra-parietal sulcus hIP1 L	99.15% Left Lateral Occipital Cortex superior division
3	-51	-75	-6	3.8	5.1	1755	49.23% Visual cortex V5 L	96.92% Left Lateral Occipital Cortex inferior division
4	-21	-72	63	3.7	4.4	1350	80.00% Superior parietal lobule 7A L; 16.00% Superior parietal lobule 7P L	100.00% Left Lateral Occipital Cortex superior division
Experiment 2: Word-likeness effect								
1	-34	-90	-10	3.8	4.7	792	65.66% Visual cortex V4 L; 28.28% Visual cortex V3V L	72.73% Left Lateral Occipital Cortex inferior division; 25.25% Left Occipital Pole
Experiment 3: Word-likeness effect								
1	-24	-69	36	3.9	5.0	2511	35.48% Anterior intra-parietal sulcus hIP3 L; 34.41% Superior parietal lobule 7A L; 17.20% Anterior intra-parietal sulcus hIP1 L	100.00% Left Lateral Occipital Cortex superior division
2	-57	-3	48	3.7	4.6	1134	52.38% Premotor cortex BA6 L; 14.29% Primary somatosensory cortex BA1 L	88.10% Left Precentral Gyrus; 9.52% Left Postcentral Gyrus
3	-36	-90	21	3.7	4.6	1026	36.84% Inferior parietal lobule PGp L; 21.05% Visual cortex V3V L; 21.05% Visual cortex V2 BA18 L; 13.16% Visual cortex V1 BA17 L	73.68% Left Occipital Pole; 26.32% Left Lateral Occipital Cortex superior division
4	33	-96	6	3.6	4.5	864	53.12% Visual cortex V3V R; 40.62% Visual cortex V2 BA18 R	100.00% Right Occipital Pole
Experiment 2: Lexicality effect								
1	-36	34	-18	4.0	6.6	10704	54.11% Broca’s area BA45 L; 14.35% Broca’s area BA44 L	37.07% Left Frontal Orbital Cortex; 34.68% Left Inferior Frontal Gyrus pars triangularis; 16.52% Left Inferior Frontal Gyrus pars opercularis; 5.16% Left Frontal Pole
2	-6	52	28	3.8	5.5	3336		46.04% Left Superior Frontal Gyrus; 37.17% Left Frontal Pole; 5.76% Right Superior Frontal Gyrus; 5.52% Left Paracingulate Gyrus; 5.28% Right Frontal Pole
3	56	32	10	3.5	4.3	1304	85.89% Broca’s area BA45 R	60.74% Right Frontal Pole; 24.54% Right Inferior Frontal Gyrus pars triangularis; 14.72% Right Frontal Orbital Cortex
4	-40	-36	-18	3.8	5.2	976		80.33% Left Temporal Fusiform Cortex posterior division; 18.03% Left Temporal Occipital Fusiform Cortex
5	60	-34	-2	3.5	4.5	904	24.78% Insula Id1 R	61.95% Right Middle Temporal Gyrus posterior division; 22.12% Right Middle Temporal Gyrus temporooccipital part; 15.93% Right Superior Temporal Gyrus posterior division
6	44	10	28	3.5	4.1	784	70.41% Broca’s area BA44 R	43.88% Right Precentral Gyrus; 40.82% Right Inferior Frontal Gyrus pars opercularis; 15.31% Right Middle Frontal Gyrus
Experiment 3: Lexicality effect								
1	-6	-84	3	3.6	4.8	15687	23.92% Visual cortex V1 BA17 R; 21.00% Visual cortex V2 BA18 L; 19.28% Visual cortex V1 BA17 L; 7.23% Visual cortex V3V L; 6.88% Visual cortex V2 BA18 R	33.39% Left Occipital Pole; 11.02% Right Occipital Pole; 11.02% Left Lingual Gyrus; 10.50% Right Intracalcarine Cortex; 9.12% Left Occipital Fusiform Gyrus; 8.95% Right Occipital Fusiform Gyrus; 6.37% Left Intracalcarine Cortex; 6.02% Right Lingual Gyrus
2	-57	12	-9	3.7	5.3	9855	53.42% Broca’s area BA45 L; 30.14% Broca’s area BA44 L	31.51% Left Inferior Frontal Gyrus pars triangularis; 20.27% Left Inferior Frontal Gyrus pars opercularis; 16.99% Left Middle Frontal Gyrus; 15.34% Left Frontal Orbital Cortex; 6.85% Left Frontal Operculum Cortex; 6.03% Left Temporal Pole
3	-48	-72	21	3.7	5.4	7587	30.25% Inferior parietal lobule PGp L; 15.66% Inferior parietal lobule PFm L; 15.30% Inferior parietal lobule Pga L; 5.69% Inferior parietal lobule PF L	33.81% Left Lateral Occipital Cortex superior division; 24.91% Left Angular Gyrus; 18.15% Left Middle Temporal Gyrus temporooccipital part; 8.54% Left Supramarginal Gyrus posterior division; 5.34% Left Lateral Occipital Cortex inferior division
4	48	-39	9	3.5	4.4	2943	31.19% Inferior parietal lobule PFm R; 20.18% Inferior parietal lobule Pga R; 8.26% Inferior parietal lobule PF R	66.06% Right Supramarginal Gyrus posterior division; 19.27% Right Middle Temporal Gyrus temporooccipital part; 7.34% Right Superior Temporal Gyrus posterior division
5	-54	-18	-3	3.7	4.7	1593	8.47% Primary auditory cortex TE1.2 L	42.37% Left Superior Temporal Gyrus anterior division; 30.51% Left Superior Temporal Gyrus posterior division; 18.64% Left Middle Temporal Gyrus anterior division; 8.47% Left Middle Temporal Gyrus posterior division
6	36	33	3	3.7	5.1	1539	66.67% Broca’s area BA45 R	57.89% Right Inferior Frontal Gyrus pars triangularis; 24.56% Right Frontal Orbital Cortex; 17.54% Right Frontal Operculum Cortex
7	21	-99	18	3.6	4.4	783	86.21% Visual cortex V2 BA18 R; 10.34% Visual cortex V1 BA17 R	100.00% Right Occipital Pole

Note:

Cluster-level FWE-corrected at p < .05, peak-level uncorrected at p < .001. All clusters with a volume smaller than 100 mm3 (i.e., only lexicality clusters) and all lables for white matter regions (i.e., from the Juelich atlas) were omitted.

**Fig 5:**
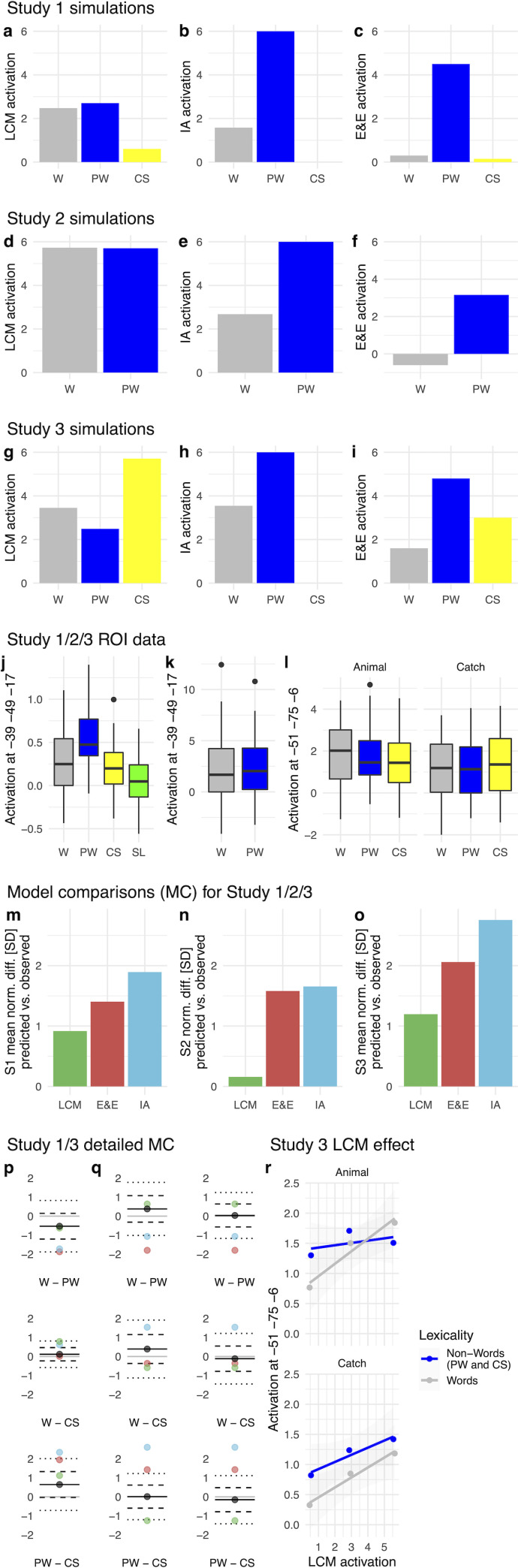
**Simulations for Words (W), pseudowords (PW), and consonant strings (CS; all medians) used in Study 1 from (a) the lexical categorization model (LCM), (b) the interactive account model (IA), and (c) the effort and engagement model (E&E). (d) LCM, (e) IA, and (f) E&E simulations for Study 2. (g) LCM, (h) IA, and (i) E&E simulations for Study 3.** Empirically observed lvOT ROI activation for W, PW, CS, and scrambled letters (SL), extracted from the same peak voxel region, i.e., defined in Study 1, of interest (ROI) of (j) Study 1, and (k) Study 2. (l) For the animal decision and catch trial detection task of Study 3 the ROI data was extracted from the peak voxel of Study 3. We present the percent signal change, in arbitrary units, for each condition, including the variance across participants (horizontal line represents the median; box +- 1 standard deviation; whiskers +- 2 standard deviations). Besides, we present model comparisons for the LCM, E&E, and IA models of Study 1 (S1) in (m), of Study 2 (S2) in (n), and of Study 3 (S3) in (o). We show the mean difference between the model simulated contrasts and the observed contrast differences from the ROI data. We standardize these model comparisons via the standard deviations of the observed data (SD). I.e., the difference between simulated and observed contrast differences in standard deviations of the observed data. For Study 1, we summarize three contrasts, which can be inspected in detail in (p), Study 2 includes only one contrast presented in (n), and Study 3 combines six contrasts shown in detail in (q). In the single contrast figures (pq), the solid black line and dot show the mean observed difference (across all participants), dashed lines +- 1 standard deviation, and dotted lines +-2 standard deviations also from the observed differences. Green dots show the LCM simulated contrast estimate, blue dots the IA contrast estimate, and red dots the E&E contrast estimates. In (q) the left panel represents the animal detection and the right panel the catch trial detection task. (r) The linear relationship between lvOT ROI activation and LCM-simulations in study 3 separated for words and non-words and the two tasks conducted in the study.

The model simulations for the two other non-linear models (IA, E&E) conducted with the identical set of stimulus materials resulted in qualitatively similar patterns (Consonant strings/CS < words/W < pseudowords/PW) as for the LCM model (see simulation results in Fig 5A–5C). However, critical quantitative differences remained, including much larger differences between modeled activations for words and pseudowords than predicted based on the LCM (compare Fig 5B and 5C to 5A). The empirically measured BOLD activation at the peak voxel of the lvOT cluster (Fig 5J) also showed the same qualitative pattern of differences as simulated by the three models, i.e., CS < W < PW (cp. Fig 5A–5C). To implement a quantitative comparison between the three models, we transformed all simulations and the empirical BOLD data to a common scale (i.e., z-transformation within each dataset to conserve relative differences but remove absolute differences). Then we compared every model’s contrast differences with the empirically measured contrast differences (i.e., W vs. PW, W vs. CS, PW vs. CS). As a difference measure, we set each contrast difference in relation to the standard deviation from the observed data (see Fig 5M; Fig 5P for all individual contrasts), allowing a quantitative comparison of the predicted vs. the observed data. As Fig 5M shows, quantitative predictions of the LCM accounted best for the empirical lvOT ROI data, yielding the lowest difference between predicted and observed data summed across all three contrasts (i.e., <1 SD). When examining these differences by contrasts, LCM predictions were best for the W vs. PW and PW vs. CS contrasts. In contrast, the E&E model was more accurate for the W vs. CS contrast (but the LCM was still within 2 SDs of the empirically measured activation difference between W and CS). In summary, the fMRI data of Study 1 indicate that the LCM provides a fair characterization of BOLD activation patterns in several lvOT voxels during visual word recognition and that it predicts activity in these voxels better than other current models of lvOT function.

#### Study 2

In Study 1, letter strings were presented in condition-specific blocks, so that the predictability of the next item concerning word/non-word categorization was high. Since the LCM does not account for the experimental context, it cannot be excluded that the blocked design may have strategically reduced the amount of processing devoted to the lexical categorization. To exclude this potential confound, we examined a second fMRI study in which stimulus categories (words and pseudowords matched for word-likeness/OLD20) were randomly intermixed (event-related design). Participants (N = 39) performed a silent reading task and had to detect a rarely occurring target word, i.e., the German word *Taste* (button). The correlation analysis from study 1, i.e., including the LCM simulated activation, did not result in any significant activation cluster. This finding was not surprising since stimuli had been selected to equalize the word-likeness between words and pseudowords with low variance.

Still, with this stimulus material, model simulations differed between the three models so that we were able to implement the model comparison analysis using the data from the peak voxel of study 1. The LCM simulations predicted high lvOT activation for both stimulus conditions with only subtle condition differences (Words>Pseudowords; Fig 5D). In contrast, both the IA and E&E models predicted substantially higher activation for pseudowords than words (Fig 5E and 5F). In the peak voxel from study 1, we found positive activation levels with virtually no differences between words and pseudowords (see Fig 5K). Model comparisons based on the word/pseudoword contrast showed that the LCM predicted the observed activation contrast best (i.e., <1 SD; Fig 5N). To summarize, here we replicated the superior prediction performance of the LCM model in an event-related design, even when no stimulus-specific difference was found in a ROI located in the lvOT.

#### Study 3

In study 1, we used stimuli with characteristics similar to previous studies. In study 2, in contrast, we included words and pseudowords with equalized word-likeness to test the LCM model in a situation in which it predicted a different pattern than alternative models. In study 3 (N = 35), we aimed at replicating this result in a study that uses stimulus materials resulting in an LCM activation pattern previously unseen in the literature and that could not be captured by the alternative models. To achieve this, we again investigated words, pseudowords, and consonant strings. We selected stimuli so that the LCM-simulated lvOT activation predicted a pattern that was inverted relative to the results predominant in the literature and observed in our study 1, i.e., with greatest activity elicited by consonant strings (CS > W > PW; cf. Fig 5G and compare to the more typically observed pattern of PW > W > CS shown in Fig 2A and 2B). If word-sensitive regions in lvOT indeed implement a word similarity-based lexical categorization process, lvOT voxels should be detectable that show this pattern of activation. Contrary to this LCM prediction, the simulations of the IA and E&E models (Fig 5H and 5I) predict different activation patterns for this specific stimulus set (IA: PW > W > CS; E&E: PW > CS > W), of which only the IA model reflects the ‘classic’ pattern predominantly found in the literature (i.e., when using ‘conventional’ stimulus sets). Second, to assess whether the depth of processing of the participants’ task (i.e., word recognition vs. semantic analysis) influences the assumed lexical categorization process, two different tasks were included in this study, an animal detection task (AD) and a catch trial detection task (CD). Each participant conducted two runs of each task, with two task sequences (AD/CD/AD/CD vs. CD/AD/CD/AD) assigned randomly to participants.

Analogous to Study 1 and 2, we used the LCM simulated activation as a single continuous predictor in a first whole-brain analysis. Again, a robust correlation of BOLD activation and LCM simulations was observed in a lvOT cluster (Fig 3D), slightly more laterally and posteriorly than in Study 1 (cp. columns 1 and 2 in Fig 4) but with a peak within 1 cm distance of the lvOT peak in Study 1 (Table 1; LCM effect Study 3, Cluster 3). We also found correlations between LCM simulations and BOLD activation in the left and right parietal cortex (Fig 3D and Table 1). Peak voxel activation of the lvOT cluster showed significant LCM correlations in both words and non-words (PW and CS combined) and both tasks (all t’s(34) > 4.3; all p’s < .001; Fig 5R). For this analysis, we estimated the activation for words and non-words separated for three LCM activation levels for each task (see blue and gray dots in Fig 5R). In this region of interest analysis, we did not find an interaction of condition and task (all t’s < 1.47; all p’s > 0.14) and no significant differences between stimulus categories (all t’s < 1.34; all p’s > 0.17; Fig 5L). Still, we found a task effect showing a higher activation in the animal detection task (*t*(34) = -2.2; *p* < 0.03). This finding is consistent with the behavioral results, which showed that the animal detection task was more demanding than the catch trial detection task, reflected in more errors (AD: 4.4%; CD: 1.5%; GLM Estimate: -0.03; SE = 0.015; *t* = 2.2) and longer response times (AD: 644 ms; CD: 520 ms; LM Estimate: -0.2; SE = 0.02; *t* = 9.7).

The model comparisons, implemented as in Studies 1 and 2, found that the LCM had, again, the lowest error in predicting the empirically observed pattern of activation contrasts (Fig 5O). Like in Studies 1 and 2, the E&E model simulated the lvOT pattern better than the IA model. In detail, of the six contrasts investigated, the LCM had three predicted contrasts within one standard deviation (SD) of the empirically observed BOLD activation difference, two that fell within 2 SDs, and one that was just above 2 SDs (Fig 5Q). In contrast, the E&E (two < 1 SD’s & four > 2 SD’s) and the IA (two < 2 SD’s & four > 2 SD’s) simulations resulted in larger differences. To protect against potential biases due to the ROI definition from the LCM contrast, we explored whether the alternative E&E and IA models predict brain activation in other lvOT voxels. To this end, we implemented two separate additional whole-brain analyses in Study 3 that correlate E&E and IA model parameters with BOLD activation. As the results in S2 Fig show, we observed no significant voxels in lvOT, neither for the E&E nor for the IA model. However, BOLD activations in frontal and bilateral lateral temporal regions correlated with the IA model simulation and left peri-central activation was associated with the E&E model parameter. Thus, the findings of Study 3 further support the proposal that involvement of lvOT subregions during visual word recognition is best described by a lexical categorization computation as implemented in the LCM.

#### Studies 1–3 –Word-likeness and Lexicality effects

Besides the LCM-based analyses, we found correlations with word-likeness (i.e., represented by OLD20; see above) in all three studies. We localized the word-likeness representations predominately to areas posterior to LCM effect in lvOT, i.e., to the posterior lvOT effect in Studies 1 (S3 Fig) and 2 (Figs 3B and 4 column 3) and parietal cortex (Fig 3E, see also Table 1). In Study 3, we also observed a correlation between OLD20 and BOLD signals in the sensorimotor cortex. A dichotomous lexicality effect with larger activity for words (W) than non-words (PW and CS), was found most consistently in brain regions downstream to lvOT in the inferior frontal cortex (Fig 3C and 3F and Table 1). In Study 2, we found additional activation for words in the Superior frontal gyrus (Fig 3C). In Study 3, we observed activation increases for words in the left superior temporal gyrus and extensive activity in the left and right occipital cortex. (Fig 3F).

In sum, empirical data from three fMRI studies support the proposal that there is a region in lvOT that implements a lexical categorization process which operates upon an estimate of word-likeness computed by upstream, more posteriorly located brain regions. Within the here-proposed LCM model, this lexical categorization process is modeled quantitatively as an entropy measure reflecting the difficulty of lexical categorization given the input’s word-likeness (see Fig 1), and LCM simulations of three different stimulus sets have accounted better for lvOT activation patterns than two alternative models. Of note, this LCM-based prediction of lvOT activity was obtained without restricting the search space to this area, but rather in whole-brain analyses, whereas only in Study 2, where no activation difference was expected due to stimulus characteristics, the LCM analysis was based on ROI data. To avoid bias, we demonstrate with further whole-brain analyses in Study 3 that competitor models do not account for lvOT activity (S2 Fig). Also, word-likeness accounted for BOLD activation differences in brain regions posterior to the LCM effect, which is consistent with the proposal that word recognition processes in lvOT operate upon a previously computed word-likeness estimate. Furthermore, we found dichotomous lexicality (Words > Non-words) effects anterior to the LCM effect (e.g., in left inferior frontal regions), which is consistent with a further assumption of LCM, i.e., that the lexical categorization process ‘selects’ orthographic input for further linguistic processing. In summary, we conclude that the empirical evaluation based on three fMRI datasets provides support for the LCM.

### Training-based evaluation

**Fig 6:**
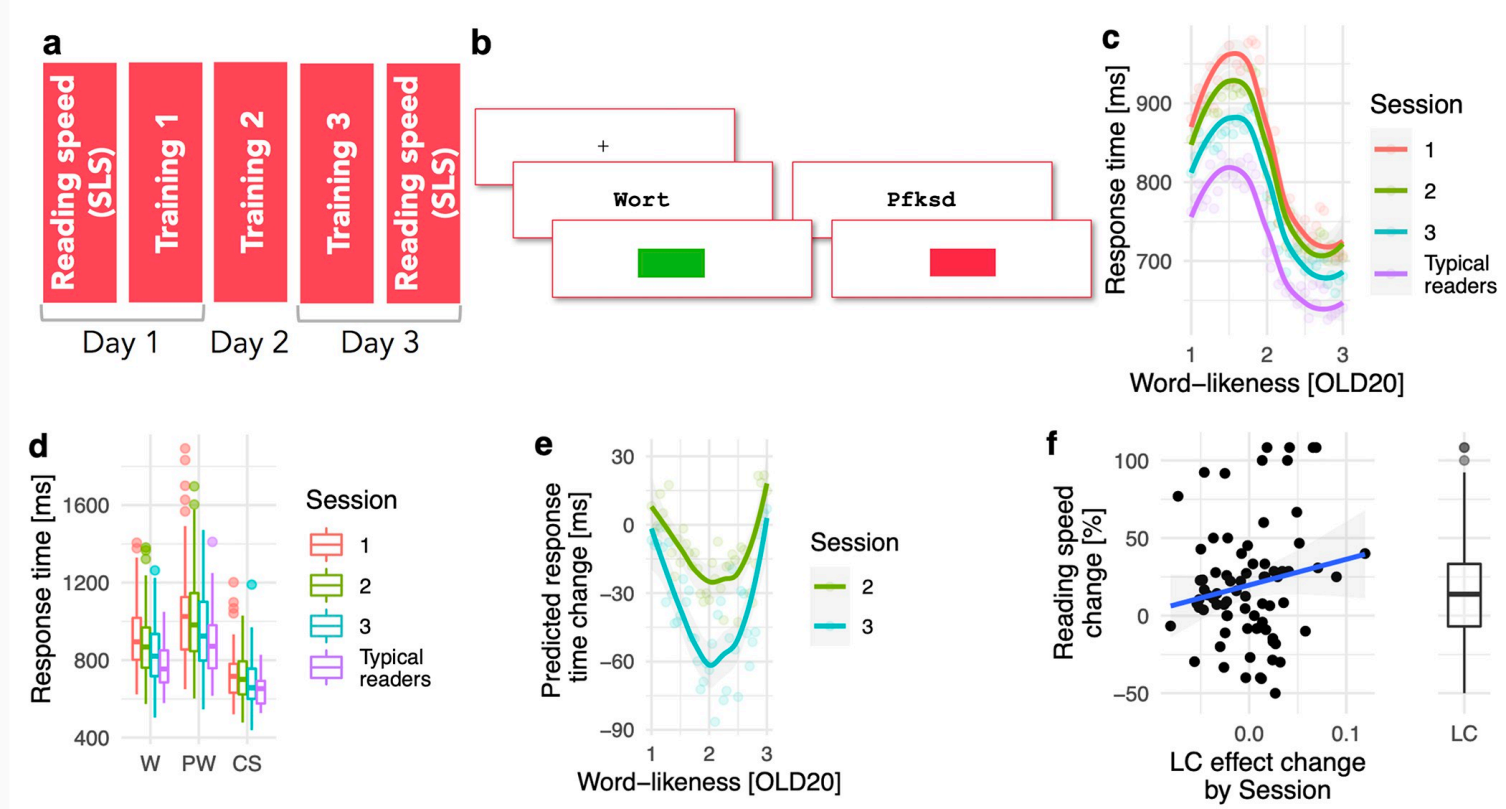
Training-based Evaluation. Behavioral training of lexical categorization: Paradigm and results. (a) Temporal structure of the training study, including the three training sessions on three separate days, as well as pre- and post-training assessments of reading speed (based on the Salzburger Lese Screening/SLS; see Methods). (b) Lexical decision paradigm performed by the participants during the three training sessions, including feedback screens. (c, d) Lexical decision response times in relation to (c) word-likeness (OLD20; i.e., high OLD20 represents low word-likeness; see Fig 1) and (d) separated for stimulus categories (W: Words; PW: Pseudowords; CS: Consonant strings) from 76 non-native learners of German across three sessions. For comparison, data for a group of 48 native German speakers (i.e., typical readers) who completed the lexical decision task with the same stimuli are presented. (e) Predicted response time reduction with session, i.e., from session 1 to 2 (green) and from session 1 to 3 (blue), extracted from fitted linear mixed models using the *remef* function to remove all but the variance which reflects the session by entropy interaction (for details see the Methods section). (f) Change of reading speed (pre-post; see *Methods*) correlated with the individual training effect (quantified as the estimate for the LCM by session interaction from a linear mixed model with random slopes). The boxplot (right panel) shows the overall increase in reading speed on the pre-post test. For all boxplots, the horizontal lines represent the median, the boxes represent +/- 1 standard deviation, and the whiskers represent +/- 2 standard deviations.

After repeatedly finding that lexical categorization difficulty is useful to describe the activation pattern of a brain area identified as highly relevant for reading, we tested the causal role of the assumed cognitive process for reading. We reasoned that when the postulated cognitive process of lexical categorization is relevant for reading, modifications of this process should affect reading performance, i.e., here defined as reading speed (which is often used as performance measure in German, see [36]). To test this hypothesis, we implemented a training procedure for 76 non-native early-stage learners of the German language who were from diverse language backgrounds. The training started and ended with a reading speed assessment (Fig 6A). Inbetween, three training sessions of about 50 min in length were implemented on consecutive days. To train the lexical categorization process, we used a lexical decision task, i.e., the task we motivated our model from [16]. Previous research also found that performance in a lexical decision task could predict lvOT activation [22]. To reinforce lexical categorization behavior, we implemented feedback that rewarded correct lexical decisions with a green square and indicated incorrect responses with a red square (Fig 6B). For comparison, we included a lexical decision dataset with the same stimuli (800 words and non-words) and a task from a sample of native German readers (N = 48) from one session.

The analysis of response times from lexical decisions showed a non-linear effect of word likeness (i.e., OLD20) for native and non-native readers, with longest response times in an intermediate range of word-likeness as predicted by the LCM (Fig 6C; see also [16]). For the non-native readers, we found a significant interaction of the lexical categorization difficulty predictor derived from the LCM and training session, indicating that lexical categorization becomes more efficient with training. This interaction effect is most obvious for intermediate levels of word-likeness levels, which represent mostly words and pseudowords and which is characterized by categorization difficulty due to overlapping word similarity distributions (Estimate: -0.03; SE = 0.01; *t* = 2.4; See Fig 6D and 6E). Note that to model this non-linear relationship between OLD20 and response times, we used the first and second polynomials, i.e., the linear and squared OLD20.

Response times also became faster from session to session (main effect; Estimate: -0.04; SE = 0.005; *t* = 8.0). However, non-native readers only reached the performance of native German readers when processing non-words (see Fig 6D; equivalence test for a subset containing only pseudowords from session three and the data from typical readers: Upper bound: *t*(122) = -2.3; *p* = 0.012; Lower bound: *t*(122) = 3.2; *p* = 0.001; for consonant strings: Upper bound: *t*(122) = -2.6; *p* = 0.005; Lower bound: *t*(122) = 2.8; *p* = 0.003). For words, no significant group differences could be found (*t*(122) = 1.7; *p* = 0.085) but also equivalence could not be established (Upper bound: *t*(122) = -1.0; *p* = 0.166; Lower bound: *t*(122) = 4.5; *p* < 0.001), as we did not find a significant difference from both equivalence bounds. Still, note that the comparisons of word-related response times from sessions 1 and 2 between language learners and typical readers resulted in significant differences (both t’s > 3.7; p’s < .001).

Next, we implemented a linear mixed effect model that included estimating the random slope of the interaction term (i.e., the lexical categorization difficulty by training session interaction). Including this parameter in the model describing the reaction times in the training allowed us to estimate the increase of the lexical categorization effect with training for each non-native reader. This individualized estimate represents how strong the quadratic lexical categorization effect increased with training. This predictor allowed us to determine if the LCM-based training effect transfers to an increase in reading speed (determined using the pre/post-training reading speed test; see *Methods*). We estimated a regression analysis with reading speed change as the dependent variable and the effect estimates of the individual lexical categorization by session interaction effects, the pre-training reading speed, and their interaction as independent variables. We found no significant interaction (GLM Estimate: -25.8; SE = 13.4; *t* = -1.9; *p* = 0.058) but significant main effects of pre-training speed (GLM Estimate: -1.1; SE = 0.5; *t* = -2.3; *p* = 0.022) and of the individual lexical categorization by session interaction effects (GLM Estimate: 416.7; SE = 205.4; *t* = 2.0; *p* = 0.046; see Fig 6F) on the pre-post change in reading speed. Overall, on average, the increase in reading speed after the lexical categorization training was 20% (*t*(75) = 4.5; *p* = 0.001; Fig 6F right panel). In sum, this final evaluation of the LCM suggests that the process of lexical categorization contributes to efficient reading. This finding supports the LCM’s assumption of lexical categorization as a core process of visual word recognition.

## Discussion

In the work reported here, we used model-based fMRI analysis to clarify the nature of computations implemented in (subregions of) left-ventral occipitotemporal cortex (lvOT) in response to orthographic percepts (letter strings). To achieve this, we implemented the four most prevalent theories of lvOT function during language processing (e.g., [11]), the most widely accepted psychological model of visual word recognition (i.e., the Dual Route Model; [27]), and a new hypothesis, the lexical categorization model (LCM), as computationally explicit models and performed direct model comparisons using different types of data. The newly proposed LCM model postulates that word-sensitive lvOT areas in the vicinity of the functionally localized visual word form area implement a categorization process that classifies letter strings into meaningful (words) vs. meaningless input, preventing attempts to apply downstream lexical-semantic processing to non-linguistic input. The LCM implementation makes explicit how the lexical categorization process is related to neural processing, i.e., via the estimated difficulty of categorizing a letter string.

We compared the implementations of the LCM and alternative models directly by simulating established effects from previous fMRI studies. Through its lexical categorization mechanism, the LCM successfully accounts for the intricate pattern of lvOT activation in response to a variety of different (sets of) orthographic stimuli, irrespective of their specific task context (compare, e.g., [12,14]). Most notably, the LCM model can simulate multiple published fMRI findings that have been viewed as contradictory, and it does so better than competing models. In addition, we found that alternative models implementing processes that in principle allow for non-linear activation patterns (IA model, [15]; E&E model, [4]) performed better than linear models like the local combination detector model (e.g., [11]). Furthermore, we demonstrate that the LCM reliably predicts empirically measured BOLD fMRI activations in voxels of the mid-lvOT and outperforms alternative non-linear models in three experiments that differed concerning the selection and composition of stimulus sets (in both whole-brain and peak-voxel defined ROI analyses). We observed that word-likeness, i.e., the quantitative psycholinguistic property on which the LCM’s lexical categorization process is based, is predominantly represented in regions posterior to the brain regions showing a LCM effect. In contrast, a dichotomous lexicality effect with increased activity for words is implemented in brain regions anterior to the lvOT subregion associated with the LCM effect. These findings are consistent with the proposal that lvOT’s categorization process gates known words into subsequent stages of lexical processing (see also [37]).

**Fig 7:**
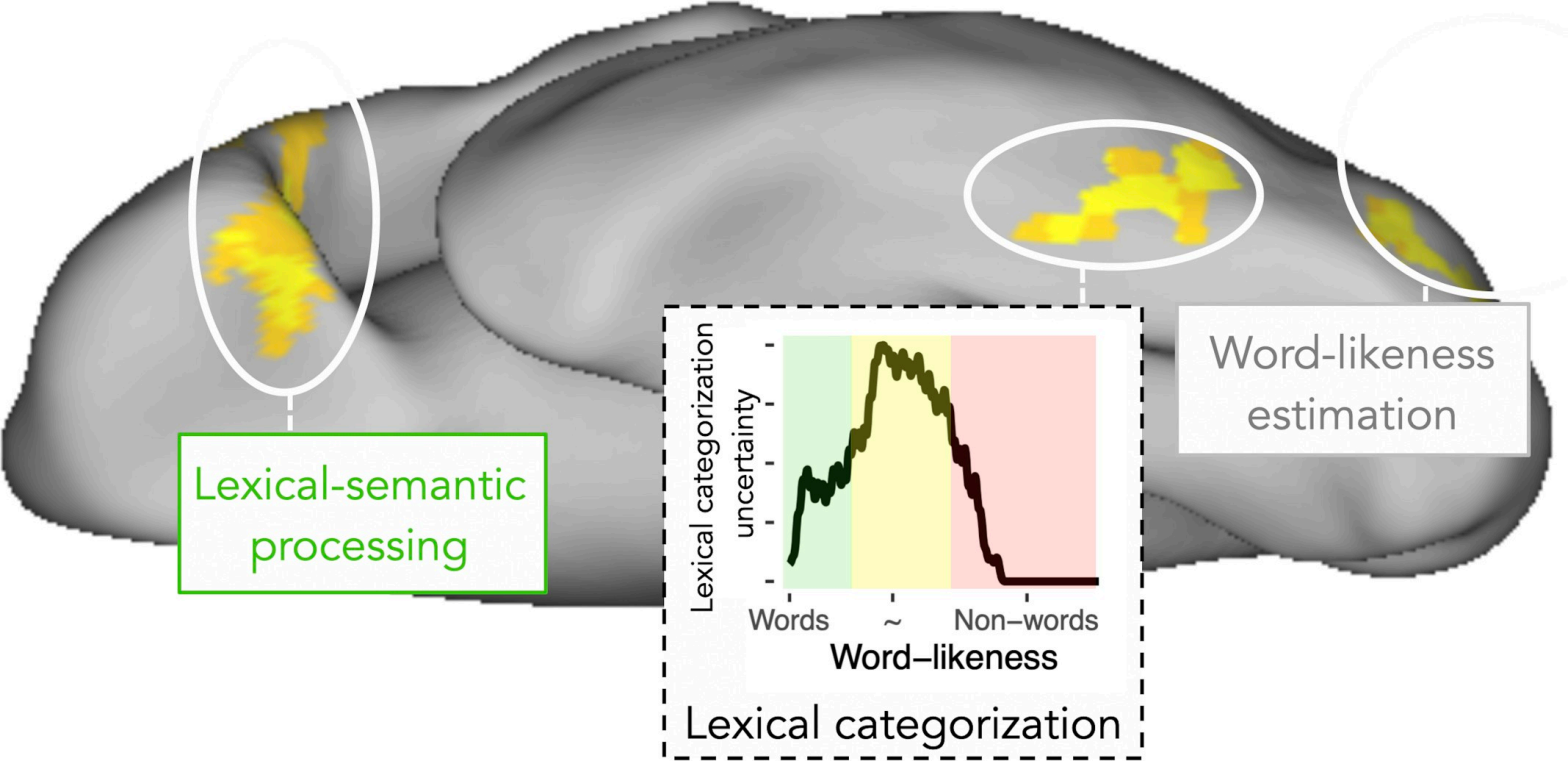
**Schematic description of processes during visual word recognition as assumed in the lexical categorization model**. The figure includes (i) word-likeness estimations in posterior visual-perceptual regions, (ii) lexical categorization in the left ventral occipito-temporal cortex (lvOT), and (iii) the extraction of word meaning in anterior regions including temporal and inferior frontal cortex. The lexical categorization process implemented in lvOT is schematically represented by the uncertainty (entropy), to visualize the LCM’s assumption that higher degrees of categorization uncertainty–in sections of intermediate word-likeness (yellow)–may require further elaborative processing to reach a lexical categorization.

Finally, we demonstrate that repetitive behavioral training based on lexical decisions, which involve lexical categorization as a core process, significantly increases reading speed. Given that reading speed as tested in this study does not explicitly involve word/nonword decisions, we argue that this training study can provide causal evidence for an involvement of lexical categorization in the process of reading. Overall, these findings suggest that during reading, following the initial stages of visual-perceptual processing, posterior brain regions compute an estimate of the word-likeness (relative to the known lexicon of words). According to the LCM model, this word-likeness estimate is fed forward to a lexical categorization process implemented in a subregion of lvOT, which in turn precedes the extraction of word meaning (lexical access) at downstream cortical sites, including more anterior temporal and frontal cortex of the left hemisphere (Fig 7).

The LCM is a quantitatively explicit model of the role of lvOT in visual word recognition. It allows for explicit comparisons to alternative models, given a specific set of stimuli. Notably, the LCM outperforms, both concerning simulating published data and modeling empirical data, multiple alternative models [4,11,15,27,30], most of which have been implemented computationally here for the first time. The comparisons based on model simulations of published BOLD activation effects showed a clear advantage for models that assume a non-linear response profile of the lvOT, i.e., LCM, the interactive account [15], and the engagement & effort model [4]. Among these three models, the LCM performed best in our fMRI data-based model comparisons.

Our work differs from previous approaches by considering a wider range of orthographic word-likeness (i.e., ranging from frequent words to pseudowords to consonant strings), whereas previous studies mostly focused only on parts of the word-likeness distribution (e.g., high vs. low-frequency [14,33,34] or words vs. pseudowords [31,32]). Also, we used a more optimal word-likeness estimate (i.e., the orthographic Levenshtein distance/OLD20 as compared to, e.g., quadrigram frequency), as is evident from previous comparisons of different word-likeness measures based on behavioral data [29]. We complemented this investigation with LCM simulation studies using alternative word-likeness estimates (see S4 Fig). The OLD20 word-likeness estimate is theoretically meaningful, as it is a reasonable proxy for the perceptual familiarity that one acquires while becoming an efficient reader [38]. On the other hand, we have argued in previous work that OLD20 may lack neuronal plausibility as it potentially subsumes multiple cognitive processes (pre-lexical and lexical; e.g., see [18]), as suggested by our finding that OLD20 correlates not only correlates with other word-likeness characteristics but also with lexical frequency [18]. To explore this in somewhat more depth, we have complemented the present investigation with LCM simulations based on three widely accepted alternative estimates of orthographic word-likeness, i.e., Coltheart’s N, trigram frequency, and quadrigram frequency. As S4 Fig shows, OLD20 captures the lvOT activation patterns best (in terms of correctly predicted effects). Also, one could criticize that OLD20 estimations are based exclusively on a lexicon of words while in the context of the LCM, they are also used to model brain responses to non-words. However, we think that this plausibly mirrors processes during visual word recognition, which have been shaped based on the words we have learned. The processes assumed by the LCM should, thus, also function at different stages of development (i.e., based on different lexicon sizes albeit at varying levels of precision). The effects of different lexicon sizes on LCM simulations are reported in S5 Fig. As can be seen, words, pseudowords, consonant strings, and pseudo-homophones result in the expected pattern (PW = PH > W > CS) only after at least 10,000 words (~ 5% of the full lexicon) were included in the lexicon (i.e., which was the basis for the OLD20 estimation). In this context, it is also noteworthy that stable LCM simulations can be achieved with lexicon sizes as small as ~ 8% of the lexicon used in the present work (*S6 Fig*). The LCM’s entropy estimation not only reflects how our brain responds to words, but also how non-words are processed. We think that including non-word distributions is essential, as the empirical tasks that the model simulates included both words and non-words. S1 Text demonstrates that an LCM without OLD20 distributions for non-words cannot reproduce core benchmark findings from the literature (like greater activity for pseudowords as compared to words). However, expectations of the reading material presented in different task contexts may shape the cognitive processes engaged. We propose that these and similar issues involving task, stimulus, and lexicon effects on lvOT activation can be systematically investigated in future work using the quantitative approach introduced here.

At its core, the LCM-simulated activation reflects the difficulty of the process of lexical categorization based on the word-likeness of the orthographic input. It is easy to identify a letter string as a meaningful word when the word-likeness (i.e., orthographic familiarity) is high. It is also easy to categorize a letter string as meaningless when early visual processing stages identify it as unfamiliar given what we have learned, i.e., the lexicon of words we have acquired so far. Both describe efficient cases of lexical categorization of letter strings, resulting in fast responses. At intermediate levels of word-likeness, however, lexical categorization difficulty is high. The LCM postulates that this results from similar orthographic-perceptual familiarity of words and non-words fed into (word-sensitive subregions of) lvOT from lower levels, making a more detailed analysis of the orthographic input necessary. In the LCM implementation, this is accounted for by the partly overlapping distributions of word-likeness (OLD20) of words and non-words. The additional processing required by letter strings with intermediate word-likeness results in increased lvOT activation and slower responses, a proposal consistent with seminal behavioral work which proposed spelling information as a potential source aiding categorization when word-likeness information is insufficient [16]. This, in turn, is compatible with our observation from fMRI Study 3, in which we used stimulus materials that were particularly difficult to categorize (e.g., Consonant strings with a high word-likeness). In this experiment, we observed that a parietal network that likely represents a dorsal sensorimotor interface connecting auditory-phonological with articulatory representations [39] co-activates with lvOT when lexical integration difficulty increases. This parietal co-activation, interestingly, also relates our results to the recent proposal of a posterior/anterior dissociation within the word-selective ventral occipito-temporal cortex [37,40]: Whereas posterior lvOT (also referred to as VWFA-1 by [37,40]) has been characterized as implementing pre-lexical processing during visual word recognition and was shown to be structurally connected to the parietal cortex [37,41], the anterior lvOT (VWFA-2) shows selectivity to lexical stimulus properties [37,40]. The LCM-correlate identified in lvOT in the present study (Study 1: y = -64; Study 3: y = -75) is located close to the posterior lvOT subregion (Ref. [37]: y < -63; Ref. [40]: y ~ -69), suggesting a primarily prelexical nature of the categorization process implemented in the LCM. A proposal that is in line with the functional profiles of reading-related brain regions identified with EcOG electrodes [3,42]. Note, however, that these group-level effects also need to be interpreted in light of substantial inter-individual differences in functional localization of word-selective activity (e.g., [41]), so that the association between LCM effects and posterior lvOT/VWFA-2 should be replicated based on individual functional localizers.

Various computational models have described cognitive processes in higher vision implemented in the vOT. These models are concerned with visual word recognition (e.g., [23,43]), object or face recognition (e.g., [44–46]). Current computational cognitive model approaches are often based on deep neuronal network models (DNNs) that can investigate complex system behavior and differences in computational architectures [47,48]. Also, these models achieve highly accurate predictions for vOT activation patterns during object recognition (e.g., [49]). Still, DNNs typically include numerous free parameters fitted in a specific training setup, i.e., typically in an image classification task. This characteristic of DNNs comes at the cost of reduced model transparency and explainability (e.g., [47,50]). More transparent approaches, like the *Template model* used by Yeatman and Kay [23], have a similar scope, i.e., an image input and a categorical output (word vs. face), but achieve this with only a limited number of free parameters (N = 3). The model described here shares this aspect of greater transparency and explainability. Developing the LCM was under the premise that the model should be as simple as possible in the implementation and as deterministic as possible (i.e., with a minimal number of free parameters). Given that the LCM model output only depends on the words stored in the lexicon, it is deterministic in the sense of not including any free parameters and thus also fully transparent. However, the current implementation of LCM lacks critical model infrastructure, i.e., an interface for image inputs or a decision algorithm.

The current implementation of the LCM model is limited by the fact that it cannot, at present, account for individual differences in reading abilities, reading experience, or knowledge of words. These limitations, however, are not principled limitations but a pragmatic decision to demonstrate the general feasibility and predictive value of the proposed lexical categorization process. Fitting the model to actual participants’ performance will require estimating parameters related to lexicon size, as these are the basis for deriving the word similarity parameter, which in turn is the basis for the lexical categorization process. The present results may also have implications for understanding dyslexia, i.e., a developmental deficit in reading performance. People who have dyslexia are characterized by altered structure and function in the subregion of lvOT that we related in the present work to the process of lexical categorization [5,6]. Consequently, novel hypotheses concerning the nature of the deficit(s) underlying dyslexia might emerge from LCM-based studies of the process of lexical categorization or the word-similarity signal on which the lexical categorization process operates. Extending the LCM model to fit individual subjects’ behavioral performance patterns would be interesting to test such hypotheses (i.e., as implemented here [51]). Also, modeling subject-specific behavioral performance would provide a novel approach to quantitative, model-driven diagnostics from which personalized training schemes could be derived.

Also, our current work was restricted to the German language. However, there are no principled reasons why the proposed lexical categorization process should apply more to (the orthography of) one language than others. Thus, future work should also explore the LCM model in other languages and orthographies (e.g., [51] for a potential use case). Lastly, the present work may also potentially be driven by the specific nature of pseudowords and non-words used in our work. However, during stimulus construction, we aimed to represent a wide variety of letter strings (concerning their word-likeness) so that the LCM evaluations reported here should be broadly generalizable. On the other hand, future work could test various types of pseudo- or non-words that, e.g., differ on specific word characteristics like morpheme structure.

The quantitative model comparison method applied in the present work is a straightforward way to compare models that assume different representations or different cognitive processes. In the context of the present work, this can, for example, be used to explore which representations are the most plausible input into the proposed categorization process (cp. *S4 Fig*) or the effects of different lexicon sizes on this process (*S5 and S6 Figs*), which may be relevant for understanding reading development. Such progress is hardly possible when models are exclusively based on verbal-metaphorical descriptions of cognitive processes. All models reported here are publicly available as code so that researchers can use them to test alternative and possibly more refined models. A crucial final stage of this approach, in addition to demonstrating the fit between model and data, however, will be to demonstrate causality of the assumed processes. The training-based evaluation we have reported in the present study is one plausible approach for achieving this, as it demonstrates the effect of manipulating the assumed process on behavioral performance in the respective domain.

In conclusion, the lexical categorization model (LCM), which is inspired by hierarchical (i.e., from occipital to anterior temporal to frontal regions) models of ventral visual stream processing, is a simple computationally explicit model that reliably describes lvOT activation patterns during visual word recognition elicited by a wide range of different letter strings. The LCM succeeds in simulating published seminal (and seemingly contradictory) fMRI results and is supported by empirical evaluations using tailored stimulus sets that differentiate between alternative models. Our empirical fMRI data suggest that a neural representation of word-likeness is estimated in brain regions posterior to word-sensitive lvOT cortex. This word-likeness representation is then fed forward into a lexical categorization process, which we could localize with new empirical fMRI data to a subregion of lvOT. Finally, after non-words are ‘filtered out’ by this categorization process, familiar words are passed through to higher-level cognitive processes such as lexical access and the extraction of word meaning, which are postulated to occur in downstream (temporal and possibly inferior frontal) areas. This framework (schematically visualized in Fig 7) is a significant step towards brain-based computational accounts [52] of information processing during visual word recognition and reading. It thus may in the future serve as an empirical basis for new interventions to help slow readers.

## Methods

### Ethics statement

Participants gave their written informed consent and received student credit or financial compensation (10€/h) as an incentive for participating in the experiments. The research was approved by the ethics board of the University of Salzburg (EK-GZ: 20/2014; fMRI studies 1 and 2) and Goethe-University Frankfurt (#2015–229; fMRI study 3; # 2019–65; Behavioral training study).

### LCM implementation

Given that previous work indicated a relationship between lvOT processing and word-*similarity* [12] or word-*familiarity* [14], the implementation of the LCM relies on an optimal measure of word-likeness–the mean Levenshtein distance over the 20 nearest words (i.e., the 20 words with the lowest distance; OLD20) [29]. Word-likeness distributions were derived for a larger number of orthographic strings by first estimating the Levenshtein distance, i.e., a measure of the similarity of any two strings of letters based on the number of insertions, deletions, substitutions, and/or transpositions of two adjacent letters [53]. OLD20 is considered superior to other established lexical measures [29], such as e.g., Coltheart’s N, as it was shown to be the better predictor of behavioral word recognition performance. Also, this measure allows us to differentiate between a large range of orthographic stimuli (compare, e.g., Fig 1A with the left panels of S4 Fig). For example, most non-words and a large number of words have zero orthographic neighbors. Thus, qualitatively very different letter strings (e.g., words as well as consonant strings; S4 Fig) have the same number of neighbors (i.e., zero). In contrast, the OLD20 can differentiate between a broader range of letter strings by describing more subtle differences in word-likeness (see above). The OLD20 is also a useful proxy of perceptual familiarity. E.g., the lexicon’s size, which is small for beginning readers and large for skilled readers, is the basis for the OLD20 estimation. When a reader has a small lexicon, the probability of finding a high number of similar words is very low since the estimation includes the 20 nearest words. With more words in the lexicon, one is more familiar with the orthography allowing a robust differentiation between words, i.e., relevant language items, and non-words, i.e., noise (see S5 Fig for simulations).

OLD20 was estimated for all German five letter uppercase words (n = 3,110; extracted from N = 193,236 words of the SUBTLEX database [54]; estimated using the *old20* function of the *vwr*-package [55] in GNU R). For each of the selected words (e.g., *Augen*—eye), we also generated a pseudoword by replacing vowels to form phonotactically and orthographically legal but meaningless letter strings (e.g., *Augon*). Pseudowords were created automatically by replacing the vowels with other vowels until the string could not be found in the SUBTLEX database anymore. Pseudowords were then revised manually based on visual inspection in order to identify illegal letter combinations. Consonant strings (i.e., orthographically illegal strings of letters; e.g., *Zbgtn*) were formed by replacing all vowels with randomly selected consonants before also computing OLD20 for each item.

Fig 1A displays the word-likeness (OLD20) distributions of these three groups of letter strings. It displays the variability of this word-likeness estimate (compare S4 Fig for distributions of the same words for alternative word-likeness estimations). Some words like *Leben* (life) are more prototypical, i.e., reflected by a high word-likeness (OLD20 = 1). Others like *Fazit* (conclusion) are less prototypical, which is reflected by a lower word-likeness (OLD20 = 2.3). Note that since the OLD20 is a distance measure, higher values represent less word-like letter strings and lower values highly word-like letter strings. Some pseudowords like *Mades* (base word *Modus*/mode) are highly similar to existing words (resulting in intermediate word-likeness; OLD20 = 1.6), while most consonants strings are dissimilar to the existing words (low word-likeness for *Zbgtn*: OLD20 = 2.95). Fig 1A demonstrates that OLD20 distributions of words (grey) and pseudowords (blue) overlap strongly in intermediate familiarity ranges, consistent with the description provided by Balota and Chumbley [16]. Letter strings with the highest word-likeness are words, and, expectedly, consonant string non-words (yellow) have the lowest levels of word-likeness. Thus, the LCM rests on the assumption that for these items, one can implement lexical categorizations (word non-word decisions) with high certainty. At the same time, lexical categorization is harder at intermediate word-likeness levels.

We propose that the information-theoretical concept of entropy [56] can describe the non-linear response profile of lvOT. We base this proposal on the assumption that lvOT implements lexical categorization to filter out perceived letter strings that are not known. Also, we assume that the uncertainty associated with this filter function in the face of overlapping distributions at intermediate levels of word-likeness reflects the difficulty (i.e., the effort) to implement the lexical categorization. Initially, entropy measures were used to determine the information value of an upcoming event in a time series. For example, in a binary categorization, if at a time point *t* the received information already allows a perfect categorization, the expected additional information value of *t*+1 is low (i.e., low entropy). In contrast, if previous information is ambiguous and does only allow a categorization around chance, the expected additional information at *t*+1 is high (i.e., high entropy) as this information might be critical for a future categorization [56]. For the current implementation, the previous information is defined by all known words–i.e., by the mental lexicon, here approximated by 3,110 five-letter words as described above. Each perceived letter string–be it a word or a non-word–can be characterized by its word-likeness, which can be quantified relative to the existing lexical knowledge, approximated in the present model by the OLD20 measure. The estimated entropy reflects the uncertainty of the lexical categorization given the word-likeness of the letter string. Note that these estimations are an approximation, based only on a subset of all possible non-words with five letters (i.e., the same number of words, pseudowords, and consonant strings: 3,110). Still, these non-words are, to some extent, related to words from the lexicon since these were the basis for constructing our non-words. Thus, non-words represent a potential source of noise added to a system based only on information from words (e.g., OLD20 estimation is related to the word items in the lexicon).

As displayed in Fig 1B, real words (grey line), most words have a high probability of being categorized as words tend to have high word-likeness (Fig 1A). On the other hand, non-words (blue line) tend to be less word-like and are thus clearly less likely to be categorized as words. As outlined above, the lexical categorization uncertainty is particularly high at intermediate levels of word-likeness. This relationship between word-likeness and lexical categorization uncertainty is captured well by the entropy estimation, represented by the black line in Fig 1B. Entropy is low when the word-likeness estimate allows a precise categorization (as either word or non-word). Only when the word-likeness estimate indicates a considerable uncertainty concerning the lexical categorization, the entropy is high. Of note, the shape of the entropy function over word-likeness strongly resembles the non-linear response profile of lvOT discussed in the Introduction section and described behavioral performance [16,18].

As a consequence, we here propose, as a central postulate of the LCM, that the difficulty of the lexical categorization computation (i.e., described by the entropy function; Fig 1B) drives the neuronal activity in lvOT. Critical here is the assumption that the lexical categorization is performed based on the word-likeness of a given letter string, only. Importantly, this entropy (*Ei*) function allows to formalize the non-linear activation profile of lvOT:

$$E_{i}=-p_{i}(W|{OLD20}_{i})∙{log}_{2}\;p_{i}(W|{OLD20}_{i})-p_{i}(nW|{OLD20}_{i})∙{log}_{2}\;p_{i}(nW|{OLD20}_{i})$$ (1)

The computational implementation of the LCM consists of the entropy function (Fig 1B, black line) derived from the probability (p_i_) of a letter string *i* being a word (W) or non-word (nW) given the specific letter string’s OLD20 (*log*_2_ indicates a logarithm on the basis of 2). *p*_*i*_(*W*|*OLD*20_*i*_) was derived by (i) taking all letter strings of a given OLD20 (e.g., at an OLD20 of 1.5 this would be 137 letter strings), (ii) identifying the words, (iii) counting them (n = 116), and (iv) calculating the probability of being a word given the OLD20 value (*p*_*i*_(*W*|*OLD*20_*i*_) = .85; *p*_*i*_(*nW*|*OLD*20_*i*_) is the inverse, i.e., 1 - .85 = .15).

### Participants

15, 39, and 35 healthy volunteers (age from 18 to 39) participated in Experiments 1, 2, and 3 of the fMRI-based Evaluation, respectively. All had normal reading speed (reading score above 20th percentile estimated by a standardized screening; unpublished adult version of [57]), reported absence of speech difficulties, had no history of neurological diseases, and normal or corrected to normal vision. In the Training-based Evaluation, 76 healthy non-native German speaking and 48 native German speaking volunteers (age from 18–74) participated. The non-native German speaking participants were all German language learners from diverse background (Arabic, Azerbaijani, Bulgarian, Chinese, English, Farsi, French, Georgian, Indonesian, Italian, Japanese, Persian, Russian, Serbo-Croatian, Spanish, Turkish, Ukrainian, Hungarian, Urdu, and Uzbek). Also, note that six of the non-native participants became literate without the acquisition of an alphabetic script. Overall, the non-native participants had a low reading score (i.e., < percentile of 30). Selecting this group was indented as we expected that lexical categorization is well established in experienced native German readers.

### Materials and stimulus presentation

#### Evaluation based on simulations

(i) Pseudowords>words contrast was implemented by contrasting LCM simulations of the 3,110 words and 3,110 pseudowords presented in Fig 1A. (ii) Words>consonant strings was implemented by contrasting LCM simulations of the 3,110 words and 3,110 consonant strings presented in Fig 1A. (iii) Pseudohomophones>words and (iv) pseudohomophones = pseudowords contrasts were implemented by contrasting LCM simulations of 3,110 words, 3,110 pseudowords, and 52 pseudohomophones (e.g., *Taksi*), which encompassed all 5-letter pseudohomophones presented by [25]. (v) Matched pseudowords>matched words were matched on multiple lexical characteristics, i.e., number of syllables, number of Coltheart’s orthographic neighbors, frequency of the highest frequency neighbor, initial bigram frequency, final bigram frequency, and summated bigram frequency (N = 108 vs. 108), as described in the original study reporting this benchmark effect [32]. (vi) Word similarity effect simulations are implemented with three non-word conditions including 332 letter strings with low word similarity, 4,034 letter strings with intermediate word similarity, and 220 letter strings with high word similarity, as well as 267 words. The words and non-words with high word similarity were matched on quadrigram frequency, whereas words and non-words with intermediate word similarity were matched on bigram frequency. Note that we selected the maximum possible number of items in each group to implement the match. (vii) The word frequency effect, i.e., log. counts per million, was implemented as described in the original benchmark study [14], with N = 3,110 words and pseudowords each; the frequency of pseudowords was set to zero. (viii) Bigram frequency effect simulations are implemented including 3,110 words, pseudowords, and consonant strings each. LCM simulations of lvOT BOLD signal strength were computed as described above.

#### fMRI-based evaluation: Experiment 1

90 five-letter words, pseudowords, consonant strings, and words of scrambled letters were presented. In addition, 90 checkerboards and 16 catch trials consisting of hash marks (“#####”) were presented; to which participants responded by a button press and which were excluded from the analysis. Words and pseudowords were matched on characteristics like the OLD20, the number of syllables, and the mean bi-/tri-gram frequency (based on the SUBTLEX frequency database [54]). In addition, words, pseudowords, and consonant strings were matched on letter frequency. Stimuli were presented using Presentation software (Neurobehavioral Systems Inc., Albany, CA, USA) in black courier new font on a white background for 350 ms (1,000 ms inter-stimulus interval/ISI), in six blocks per stimulus category with 16 items each. After two blocks, a fixation cross was presented for 2 s. In addition, six rest blocks (fixation cross) were interspersed. Each block lasted for 16 s, which resulted in approximately 10 min of recording time.

#### fMRI-based evaluation: Experiment 2

60 critical five-letter words and pseudowords were presented. In addition, 120 different pseudowords that underwent a learning procedure and, thus, not analyzed here. 10 practice trials, and 30 catch trials consisting of the German word *Taste* (Button) were presented. Participants responded to catch trials by a button press; these trials were excluded from the analysis. All words and pseudowords consisted of two syllables and were matched on OLD20 and mean bigram frequency. Letter strings were presented by the Experiment Builder software (SR-Research, Ontario, Canada) for 800 ms (yellow Courier New font on gray background; ISI 2,150 ms). To facilitate estimation of the hemodynamic response, an asynchrony between the TR (2,250 ms) and the stimulus presentation was established. In addition, 60 null events (fixation cross as in the ISI) were interspersed among trials. The sequence of presentation was determined by a genetic algorithm [58], which optimized for maximal statistical power and psychological validity. The fMRI session was divided in two runs with a duration of approximately 8 min each.

#### fMRI-based evaluation: Experiment 3

We presented 200 critical five-letter words, 100 consonant strings, and 100 pseudowords. Besides, we presented ten practice trials and 100 catch/animal trials, each consisting of the German word Taste (Button) or animal names. Participants responded to catch trials in the catch trial task or the animal names in the animal detection task by a button press; we excluded these trials from the analysis. We selected the letter strings based on simulations from the LCM, the IA, and the E&E model so that the stimulus set allowed to differentiate between the alternative models (i.e., see Fig 5G–5I). Letter strings were presented by the Presentation software (Neurobehavioral Systems Inc., Albany, CA, USA) for 800 ms (black Courier New font on gray background; ISI 2,150 ms; i.e., same as for Experiment 3). Also, 100 null events (fixation cross as in the ISI) were interspersed among trials. A genetic algorithm again determined the sequence of the presentation [58]. We divided the fMRI session into four runs with a duration of approximately 10 min each.

#### Training-based evaluation

76 non-native speakers of German completed a three-day training procedure with one session of lexical categorization training per day (see Fig 6A). Reading skill was assessed before and after the training procedure using a frequently-used German reading speed screening procedure as a test of reading skill (Salzburger Lese Screening/SLS; Adult version of [57]). During the training sessions, words and non-words were presented in random order (presentation time until response), and participants had to indicate by button press whether or not the presented letter string was a word (lexical decision). After the button press, feedback on the correctness of the response was given (200 ms; see Fig 6B), and the subsequent trial was presented after an inter-stimulus interval of 1,000 ms (during which a fixation cross was presented). In each session, 1,600 5-letter letter strings, including 800 words, 400 pseudowords, and 400 consonant strings, were presented (total length: about 50 min per training session). Stimuli were selected randomly from the letter strings included as the basis of the LCM (i.e., all lexicon words with five letters and non-words constructed from these words; see Fig 1A). The goal of the random selection was that the word/non-word characteristics, e.g., the word-likeness (OLD20) and word frequency, represent the naturally-occurring distributions in the lexicon to avoid artificial stimulus sets and allow participants to learn based on a representative set of words. Stimulus presentation was implemented with the Experiment Builder software (SR-Research, Ontario, Canada) using mono-spaced Courier-New font, with a visual angle of approximately 0.3° per letter. The presentation order of stimuli and experimental conditions was randomized for each participant and each of the three training sessions. For data analysis, we recorded response times and response accuracies.

Analysis of behavioral training data was based on a two-step procedure. First, a linear mixed model was used to estimate the effects of LCM-based entropy, training session, OLD20, logarithmic frequency (per million from the SUBTLEX database), logarithmic stimulus presentation order, lexicality, and the correctness of the response on logarithmically transformed response times. For random effects, we followed the logic of parsimonious mixed models [59] and ended up with a random effect structure that estimated the random effects of the intercepts of stimuli and participants. To investigate the strength of the individual interaction of the LC effect, i.e., the change of the LC effect with training, we estimated the random slope of this interaction term on the participants. Using the *ranef()* function of the lme4 package in the statistics package R, one can extract each participant’s interaction effect. We used the individual interaction effects estimation for the GLM analysis that used the individual slope estimates from the linear mixed model as a regressor describing the pre-/post-reading speed assessment (see Fig 6F). For Fig 6E, we used the fitted LMM model to visualize the change in the LCM effect across sessions based on simulations using the *remef* package [60] that allows removing co-varying effects.

Note that we combined this dataset from three studies and that all stimuli including lexical characteristics are available here: https://osf.io/3cjb7/

### Data acquisition and analysis

#### LCM simulations

Statistical comparisons of the simulations presented in Fig 2 were implemented with the *lm* function in R and *p*-values were Bonferroni-corrected for multiple comparisons. In total, nine benchmark effects were tested of which the contrast pseudowords>words>consonant strings [31] was a combination of the pseudowords>words [4] and the words>consonant strings [2] contrast. Significant differences were marked in Fig 2 (also implemented for alternative models presented in the S1 Text and S4 Fig with a black horizontal bar when the direction of the effect was expected from the literature and a red bar when the expected effect direction was violated. Figs 1, 2, 5 and 6 were implemented using *ggplot2* in R.

#### *fMRI data;* Experiments 1, 2, and 3

The three fMRI datasets were measured in two different laboratories (Experiment 1 and 2: University of Salzburg; Experiment 3: Brain imaging Center, Goethe University Frankfurt) at three different time points. This resulted in variation of data acquisition parameters. For this reason, a detailed description of data acquisition parameters is provided in S1 Methods. All experiments were measured using 3 Tesla Siemens Magnetom MR scanners, using BOLD sensitive T2*-weighted gradient echo, echo-planar imaging (EPI) sequence, acquiring 36 axial slices at a TR of 2250 ms.

For experiment 1 and 2 the SPM8 software (http://www.fil.ion.ucl.ac.uk/spm), running on Matlab 7.6 (Mathworks, Inc., MA, USA), was used for preprocessing and statistical analysis. Functional images were realigned, unwarped, corrected for geometric distortions by use of the FieldMap toolbox, and slice-time corrected. In Experiment 1 the high-resolution structural image was pre-processed and normalized using the VBM8 toolbox (http://dbm.neuro.uni-jena.de/vbm8). The image was segmented into gray matter, white matter and CSF, denoised, and warped into MNI space by registering it to the DARTEL template of the VBM8 toolbox using the high-dimensional DARTEL registration algorithm [61]. Based on these steps, a skull-stripped version of the structural image was created in native space. The functional images were co-registered to the skull-stripped structural image and then the parameters from the DARTEL registration were used to normalize the functional images to MNI space. In Experiment 2 the images were co-registered to the high-resolution structural image, which was normalized to the MNI T_1_ template image. The functional images were further resampled to isotropic 3 × 3 × 3 mm voxels in the fMRI-based Evaluation, Experiment 1, and 2 × 2 × 2 mm voxels in 2, Experiment 2, and smoothed with a 6 mm full width half maximum Gaussian kernel. For Experiment 3, we first set up the fMRI data in the BIDS format [62], which allowed us to use the fMRIPrep preprocessing pipeline [63].

For statistical analysis, in Experiment 1, 2, and 3, a two-stage mixed effects model was used. The first level is subject–specific and models stimulus onsets with a canonical hemodynamic response function and its temporal derivative. Movement parameters from the realignment step and catch trials were modeled as covariates of no interest. A high-pass filter with a cut off of 128 s was applied to the functional imaging data and an AR(1) model [64] corrected for autocorrelation. In Experiment 3, we used the Python-based *nistats* package for statistical analysis and the *nilearn* package to create figures [65] to model the fMRI statistics as in the SPM software. For the statistical analysis of ROI data, LMMs [66] were calculated in R (see below).

#### Training data

Linear mixed model (LMM) analysis is a linear regression analysis that is optimized to estimate statistical models with crossed random effects for items [66]. These analyses result in effect size estimates with confidence intervals (SE) and a *t*-value. *t*-values larger than 2 are considered significant since this indicates that the effect size ±2 SE does not include zero [67].

For the training data, we used LMMs to analyze the response time data from the LCM training task (i.e., a lexical decision with feedback). First, we excluded response times below 300 ms and above 4000 ms. Second, we log. transformed the response times to account for the ex-gaussian distribution that typically results from response times measurement. For the regression model, which we used to estimate the change of the entropy effect with training, the core term was a three-way interaction of training session, entropy, and word-likeness (i.e., OLD20). Besides, we controlled for the following covariates: word frequency, lexicality, trial index (i.e., at which position in the training session the letter string was presented), and if the response was erroneous or not. Random effects were the intercepts of letter string and participant.

For this secondary analysis, i.e., correlating individual LCM training effects with reading speed increase, we estimated the random slope of the interaction of entropy with training on the participants. With this individualized interaction estimate, we now have a predictor that can investigate if the LCM specific training effects translate to a reading speed increase. For this analysis, we estimated a linear regression model that predicted the reading speed increase in percent. As predictors, we included the pre-training reading speed and the individual LCM training estimate plus the two parameters’ interaction.

## Supporting information

S1 Text Computational implementations and simulations of alternative models. (DOCX)

S1 Fig Simulations from the Dual-Route Model of reading aloud (DRC) separated for activation from (a) the orthographic lexicon and (b) the grapheme-to-phoneme conversion route of the DRC. We estimated the activation per word by the normalized correlation of model cycles and the activation level. This metric results in a low activation when the orthographic lexicon activation rises fast. The former reflecting easy access, and the latter reflecting hard access to the lexical item. We applied the same logic to the grapheme-to-phoneme route. Here a fast activation rise indicates that the letter string can be decoded fast based on the letter-by-letter decoding process. Again a fast activation rise of that route is reflected in a low correlation. In contrast, a slow rise is reflected by a high correlation, i.e., assumed high activation. Interestingly, the orthographic lexicon activation resulted in a simulated activations pattern that mimicked the lexicon model, and the grapheme-to-phoneme route the local activin detector model simulations. All but the high activation for pseudohomophones could be simulated by either the orthographic lexicon or the grapheme-to-phoneme route. Thus, one can conclude that the DRC can, when orthographic lexicon and grapheme-to-phoneme route are both implemented in lvOT, explain most of the pattern found in the lvOT by one or the other route of the model. Still, when using the reaction times from the model, i.e., the simulations using both routes, the expected contrast differences could not be simulated better than the orthographic lexicon results. Nonetheless, the simulated activation for pseudohomophones was lower than one would expect from the literature pattern. I.e., simulated activations were lower than words, pseudowords, and consonant stings in both routes.
(DOCX)

S2 Fig fMRI whole-brain analyses based on the parameters from the Interactive account (IA) and engagement and effort model (E&E). Significant correlation results between BOLD signals and parameters from the IA (upper row) and E&E (bottom row) simulations modeled as a single, continuous predictor for the data from Experiment 3. Thresholds for all whole-brain analyses: voxel-level: *p* < .001 uncorrected (cluster-forming); cluster level: *p* < .05 family-wise error corrected. No other regions than those displayed were significant.
(DOCX)

S3 Fig Word-likeness and lexicality effects for fMRI based Experiment 1. In the main text, we reported an effect of word likeness in occipito-temporal regions posterior to the word-sensitive lvOT cluster in our second and third fMRI experiment (event-related single-trial design; Fig 3A and 3E). While the blocked design of fMRI experiment 1 was not primarily designed to demonstrate such stimulus-specific effects, we nevertheless also subjected this data set to an event-related analysis of word-likeness. Word-likeness, modeled as a continues factor, produced a more widespread activation effect in fMRI study 1, distributed over occipital regions of left and right hemisphere, with greater activity for more word-like letter strings (two significant clusters: Cluster 1: peak voxel at x = -12, y = -73, z = 1; Left lingual gyrus; T = 7.34; 514 voxels; Cluster 2: peak voxel at x = -6, y = -88, z = 37; Left cuneus; T = 4.0; 67 voxels). From the ventral view of the left hemisphere, it is visible that the cluster extended into the posterior lvOT, which is not the case in the right hemisphere (Activation effects are visualized at voxel level *p* < .001 uncorrected; cluster level *p* < .05 family-wise error corrected). In addition, we tested the words > pseudowords contrast: no significant activation difference between words and pseudowords was found. Only when neglecting the cluster correction, a small activation cluster was found in left frontal cortex (x = -39, y = 38, z = 25; Left frontal pole; T = 3.7; 7 voxel). To summarize, consistent with the second fMRI experiment, an effect of word-likeness on brain activation was found posterior to lvOT, while the (weak) lexicality effect was observed anterior to lvOT, i.e., in downstream regions of the frontal lobe.
(DOCX)

S4 Fig LCM implementations and simulations based on three alternative word-likeness measures. In the main text, we report an implementation of the LCM using OLD20 (29) as a measure of word likeness that has been reported in the literature to outperform other measures of word likeness (29). However, it is also possible to implement the LCM based on alternative measures of word-likeness. Here, we report three simulations of the benchmark effects tested in Evaluation 1 (cf. Fig 2), using (a) Coltheart’s neighbors, (b) trigram frequency, and (c) quadrigram frequency, as bases for the LCM simulations. The left-most columns show the distributions of the respective word-likeness measure for different types of letter strings as well as the probabilities of being a word or not and the resulting entropy (categorization uncertainty), analogous to Fig 1 in the main text. It is visible that all three measures are less well able to distinguish between words, pseudowords, and consonant strings than OLD20 (Fig 1A) does. As a result, the resulting entropy function has a different shape than the one derived from OLD20. The LCM implementation based on OLD20 (Figs 1 and 2) clearly outperformed (in terms of correctly predicted effects and estimated effect sizes) these models based on alternative word-likeness measures. When inspecting the pseudoword > words (4) contrast, only the model based on Coltheart’s N (S5A Fig) was able to predict this difference; on the other hand, this was the only model that did not predict the contrast words > consonant strings (2). For description of labels see Figs 1 and 2.
(DOCX)

S5 Fig Effect of lexicon size on word-likeness estimations and LCM simulations. We assumed that lexicon size influences word-likeness estimations (i.e. the number of items in the lexicon to which e.g. the OLD20 is estimated) and LCM simulations. First, word-likeness distributions for words, pseudowords, and consonant strings, which were used for the LCM model (see Figs 1 and 2; see Materials section), are presented for lexica consisting of the most frequent 100, 1,000, 10,000 and 100,000 words of the SUBTLEX database. When comparing the word-likeness distributions, it becomes obvious that increasing the size of the lexicon results in a better differentiation between letter string categories (e.g., stronger differentiation between words and consonant strings). Simulations from LCM models derived from these distributions (compare to Figs 1 and S5), in the lower panels (line graphs show median LCM simulated activation), showed that the model predicted no difference between categories with very small lexicons. Lexicons with intermediate size already allow a differentiation between consonant strings (yellow) and the other stimulus categories. Starting from lexicons with 10,000 words, clearer differentiation between words (gray) and pseudowords (blue)/pseudohomophones (green) was present. In part, besides established effects such as acquired letter knowledge or grapheme to phoneme conversion (for example (4)), these simulations demonstrate that the increasing lexicon size may account for critical patterns of developmental change during literacy acquisition; our present work, in this context, suggests that the lvOT may be an important mediator of such developmental processes.
(DOCX)

S6 Fig Correlation of the LCM calculated entropy from subsets of the stimuli with the full set of the stimuli. Upper section includes the mean correlation over twenty entropy correlations based on different randomly drawn sets of orthographic stimuli. Note, the proportion of words, pseudowords and consonant clusters was always the same. Lower section shows the standard deviation across the twenty calculations. Data is shown in 1% steps.
(DOCX)

S1 Methods fMRI measurement parameters. (DOCX)
