# Supplementary material for: The lexical categorization model: A computational model of left ventral occipito-temporal cortex activation in visual word recognition
Source: PLoS Comput Biol. 2022 Jun 9;18(6):e1009995. doi: 10.1371/journal.pcbi.1009995 (PMC9182256; doi:10.1371/journal.pcbi.1009995)
Supplement: S1 Text — (DOCX) [file pcbi.1009995.s001.docx]

*S1 Text*. Computational implementations and simulations of alternative models.

*
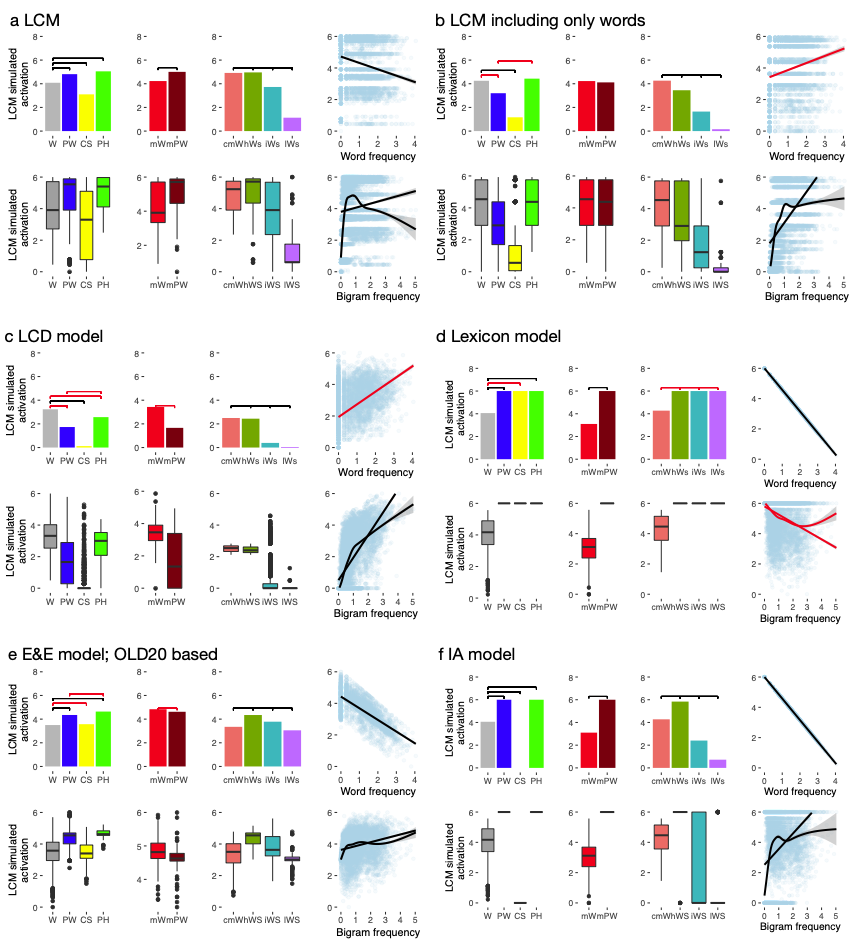
* Fig A. Simulation results from alternative models. (a) the LCM (similar as in Fig 2, adding boxplot figures), (b) an LCM variant that included only the distributions of words, (c) the local combination detector model (LCD), (d) the lexicon model, (e) the engagement and effort model (E&E), and (f) the interactive account model (IA). Above each subplot, horizontal black bars indicate simulations that resulted in the expected condition difference between letter string categories, as derived from linear models. Red bars indicate contrasts opposite to the expected direction.

In the following the details of each alternative model will be described, which allow to evaluate the LCM in contrast to alternative models of lvOT function. We implemented multiple accounts based on the verbal proposals [1,2][3,4] and a variant of the LCM which was not described previously (See Fig A for simulations). The critical difference between the LCM variant (Fig A b) and the LCM (Fig A a) presented previously (Fig 1) is that in the variant, we used only the word-likeness distribution of words as the basis for the entropy estimation. This implementation's primary motivation was that it would be reasonable to test if the entropy function can be implemented based on words only. If the variant LCM is better in simulating lvOT activation contrasts, one could argue that the representations in the lvOT are, to a great extent, words. Alternatively, i.e., if the LCM model described in the main text results in more accurate simulations, one could argue that the representations in the lvOT are, to a great extent, pre-lexical.

For the implementation, we used the cumulative probability of words based on word-likeness to estimate the probability of a sting being a word/pseudoword. In other words, we took all 3110 words and estimated the probability of all words starting with the highest word-likeness level.

Equation A:

$$p_{word} = 1-\frac{N_{max. word-likeness}}{N_{all words}}$$

In the following, we repeated this procedure for all word likeness levels to estimate the probability for a word for each bin. In the end, the cumulated probabilities are 1.

Equation B:

$$p_{word} = 1-\sum_{x=min. \overset{\to}{} max. word-likness} \frac{N_{x}}{N_{all words}}=0$$

The inverse of this probability for the string being a word is again the probability of being a non-word. After that, we can use the same equations as presented in the methods to estimate the entropy, i.e., the simulated activation of the lvOT. The simulation results show that the simulations of this words-only LCM are only correct for four benchmark effects (Fig A b; 4/9 effects simulated correctly). Thus, we learn here that the non-words distributions are a necessity for the LCM, suggesting that representations are, to some extent, pre-lexical in nature (see also [5]).

As an adequate test case for the LCM, we implemented four alternative models, i.e., two linear models: lexicon and LCD model, and two non-linear models, the E&E and the IA model. Here we focused on implementing the verbally described relationships between lvOT activation and the word characteristics central to the respective model. For the E&E and the IA models, we implemented more variants, but we only present those with the best fit. Overall, only the LCM and the IA were able to predict all nine benchmark contrasts. For qualitative model comparisons and model performance, see Fig 2F.

The *LCD mode l*[3] postulates that lvOT stores pre-lexical and lexical multi-letter representations (up to a length of about four letters). When a letter string is presented that contains these multi-letter combinations, their neural representations are activated irrespective of the lexicality of the string. Therefore, higher activations ($A_{lvOT}$) are predicted for often-occurring letter combinations in contrast to rare letter combinations. As formalized in Equation C, this is modeled by a linear increase of 0.4 per log quadrigram frequency (of a string). The specific value for this increase function was read out from Figure 4 of [5] (left hemisphere, y = -56;).

Equation C:

$$A_{lvOT} = log Qf_{string}\cdot0.4$$

This ‘(sub-) lexical storage/activation’ model of lvOT accounts for four benchmark contrasts (Fig A c). The LCD model predicts increased activation for words relative to consonant strings, as well as effects of word similarity and bigram frequency.

The *lexicon model* [6] conceptualizes the processes implemented in lvOT as a search in an orthographic lexicon. This lexicon is arranged by word frequency; accordingly, frequent words are found fast, resulting in fast response times and low lvOT activation, whereas it takes substantially longer to identify infrequent words, resulting in prolonged response times and high activation. lvOT activation ($A_{lvOT}$; cf. Equation D) decreases linearly by about 1 activation level (arbitrary unit) per 1 log frequency ($f_{string}$) unit (read out from Figure 2: Midfusiform/Posterior fusiform in [1]; $f_{string}$: frequency of the letter string, with frequencies of pseudo- and nonwords set to zero [1]; $f_{max}$: highest frequency in the lexicon).

Equation D:

$$A_{lvOT} = log f_{max} - {log f}_{string}$$

As shown in the upper panel of Fig A, the implementation of this model can account for five benchmark effects. Specifically, this ‘frequency ordered lexical search’ model obviously accounts for the word frequency effect but also for the lower activation for words in contrast to pseudowords irrespective if they were matched for OLD20 or not (black lines in Fig A d). None of the benchmark effects including consonant strings or word similarity manipulations (i.e., bigram frequency and word similarity effect) could be captured by this model.

None of the benchmark effects explained by the lexicon model could be explained by the LCD model and vice versa. Note that the patterns of predictions generated by these two models do not depend on the numeric values chosen for the change in activation, which were read out from the results figures of the original publications. Given that these two models are simple linear transformations of the respective lexical measure, the effect directions would be similar irrespective of which specific constant number would be used to predict lvOT activation in Equations D and C. In the following we present two models implementing a non-linear relationships as the basis for the activation pattern in the lvOT in response to words.

The *engagement and effort model* [7] combines two processes. One relates to the engagement of brain regions in visual word recognition. Engagement is high for letter strings that are likely words and low for i.e., consonant strings that violate basic rules for constituting orthographic stimuli (e.g., no vocals present). The second process is concerned with the effort one needs to recognize a word, e.g., a seldom word is more effortful to decode then a frequently used word. Here we used OLD20 to implement the engagement process, as the OLD20 parameter can differentiate between non-words. Note that we have to invert the OLD20 as it is a distance measure. Then a high value indicates a high word likeness, i.e., a high engagement of the region. To model the reduction of activation with reduced effort in processing, we subtracted word frequency from the engagement variables (i.e., both parameters were z-transformed). The result of this estimation is a non-linear response function of the lvOT with low activation for non-words with a low word-likeness, a high activation for pseudowords, and a reduced activation for words with a high frequency.

Equation E:

$$A_{lvOT}= {(WL}_{old20}*-1)- {log f}_{string}$$

The simulations show that the E&E model could simulate six benchmark effects correctly. This finding indicates that the non-linear models are better in explaining lvOT activation in response to various letter strings.

The *interactive account* of Price and Devlin [8] adopts a predictive coding perspective and postulates that lvOT activation reflects an interaction between top-down and bottom-up processes, in the sense of a prediction error that represents difference between, in the case of single word presentation, non-strategic general expectations derived from general word knowledge and the actual bottom-up visual orthographic input. Here we implement this by assuming bottom-up information as word frequency (i.e., often seen words are more likely expected). This reflects that bottom-up information, in standard single word presentations, is determined by predictions based on our orthographic knowledge (e.g., see [9] for an example and more deep discussion on this issue). This is as for seldom words or non-words sensory information cannot be predicted to a high extent resulting in a large prediction error. In contrast, predictions are more precise for frequent words and therefore the prediction error is reduced. In their paper, Price and Devlin state that this in only the case for letter strings that follow the basic rules of orthography. I.e., consonant strings that do not fall in this category therefore should not activate the lvOT. As a consequence this model also assumes a non-linear response function of the lvOT.

Equation F:

$$A_{lvOT} =\log f_{max}- {log f}_{string}$$

$$\left[ A_{lvOT} =0 \right] if \left[ string= consonant string \right]$$

Besides the LCM, the IA model, is, the second model approach that is able to simulate all benchmark effects. This finding once more, indicates that the lvOT follows a non-linear response profile but also strongly suggests that we need experimental data to distinguish between the LCM and the IA model (see fMRI-based Evaluation).

References

1. Kronbichler M, Hutzler F, Wimmer H, Mair A, Staffen W, Ladurner G. The visual word form area and the frequency with which words are encountered: evidence from a parametric fMRI study. NeuroImage. 2004;21: 946–953. doi:10.1016/j.neuroimage.2003.10.021

2. Kronbichler M, Bergmann J, Hutzler F, Staffen W, Mair A, Ladurner G, et al. Taxi vs. Taksi: On Orthographic Word Recognition in the Left Ventral Occipitotemporal Cortex. Journal of Cognitive Neuroscience. 2007;19: 1584–1594. doi:10.1162/jocn.2007.19.10.1584

3. Dehaene S, Cohen L. The unique role of the visual word form area in reading. Trends in Cognitive Sciences. 2011;15: 254–262. doi:10.1016/j.tics.2011.04.003

4. Dehaene S, Cohen L, Sigman M, Vinckier F. The neural code for written words: a proposal. Trends in Cognitive Sciences. 2005;9: 335–341. doi:10.1016/j.tics.2005.05.004

5. Vinckier F, Dehaene S, Jobert A, Dubus JP, Sigman M, Cohen L. Hierarchical Coding of Letter Strings in the Ventral Stream: Dissecting the Inner Organization of the Visual Word-Form System. Neuron. 2007;55: 143–156. doi:10.1016/j.neuron.2007.05.031

6. Wimmer H, Ludersdorfer P. Searching for the Orthographic Lexicon in the Visual Word Form Area. In: Lachmann T, Weis T, editors. Reading and Dyslexia: From Basic Functions to Higher Order Cognition. Cham: Springer International Publishing; 2018. pp. 57–69. doi:10.1007/978-3-319-90805-2_3

7. Taylor JSH, Rastle K, Davis MH. Can cognitive models explain brain activation during word and pseudoword reading? A meta-analysis of 36 neuroimaging studies. Psychological Bulletin. 2013;139: 766–791. doi:10.1037/a0030266

8. Price CJ, Devlin JT. The Interactive Account of ventral occipitotemporal contributions to reading. Trends in Cognitive Sciences. 2011;15: 246–253. doi:10.1016/j.tics.2011.04.001

9. Gagl B, Sassenhagen J, Haan S, Gregorova K, Richlan F, Fiebach CJ. An orthographic prediction error as the basis for efficient visual word recognition. NeuroImage. 2020;214: 116727. doi:10.1016/j.neuroimage.2020.116727
