# Supplementary material for: The lexical categorization model: A computational model of left ventral occipito-temporal cortex activation in visual word recognition
Source: PLoS Comput Biol. 2022 Jun 9;18(6):e1009995. doi: 10.1371/journal.pcbi.1009995 (PMC9182256; doi:10.1371/journal.pcbi.1009995)
Supplement: S1 Fig — We estimated the activation per word by the normalized correlation of model cycles and the activation level. This metric results in a low activation when the orthographic lexicon activation rises fast. The former reflecting easy access, and the latter reflecting hard access to the lexical item. We applied the same logic to the grapheme-to-phoneme route. Here a fast activation rise indicates that the letter string can be decoded fast based on the letter-by-letter decoding process. Again a fast activation rise of that route is reflected in a low correlation. In contrast, a slow rise is reflected by a high correlation, i.e., assumed high activation. Interestingly, the orthographic lexicon activation resulted in a simulated activations pattern that mimicked the lexicon model, and the grapheme-to-phoneme route the local activin detector model simulations. All but the high activation for pseudohomophones could be simulated by either the orthographic lexicon or the grapheme-to-phoneme route. Thus, one can conclude that the DRC can, when orthographic lexicon and grapheme-to-phoneme route are both implemented in lvOT, explain most of the pattern found in the lvOT by one or the other route of the model. Still, when using the reaction times from the model, i.e., the simulations using both routes, the expected contrast differences could not be simulated better than the orthographic lexicon results. Nonetheless, the simulated activation for pseudohomophones was lower than one would expect from the literature pattern. I.e., simulated activations were lower than words, pseudowords, and consonant stings in both routes. (DOCX) [file pcbi.1009995.s002.docx]

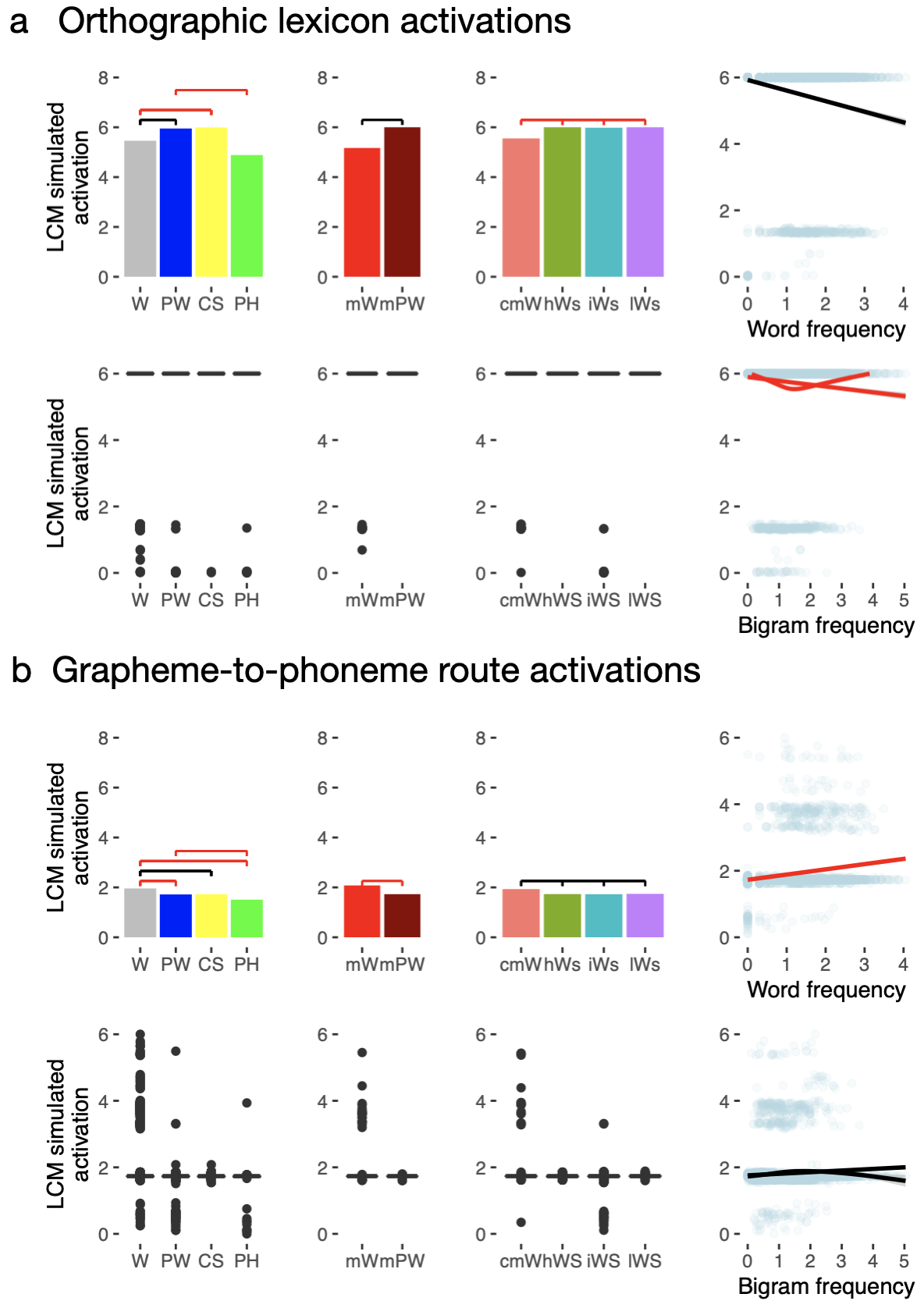


*S1 Fig*. Simulations from the Dual-Route Model of reading aloud (DRC) separated for activation from (a) the orthographic lexicon and (b) the grapheme-to-phoneme conversion route of the DRC. We estimated the activation per word by the normalized correlation of model cycles and the activation level. This metric results in a low activation when the orthographic lexicon activation rises fast. The former reflecting easy access, and the latter reflecting hard access to the lexical item. We applied the same logic to the grapheme-to-phoneme route. Here a fast activation rise indicates that the letter string can be decoded fast based on the letter-by-letter decoding process. Again a fast activation rise of that route is reflected in a low correlation. In contrast, a slow rise is reflected by a high correlation, i.e., assumed high activation.

Interestingly, the orthographic lexicon activation resulted in a simulated activations pattern that mimicked the lexicon model, and the grapheme-to-phoneme route the local activin detector model simulations. All but the high activation for pseudohomophones could be simulated by either the orthographic lexicon or the grapheme-to-phoneme route. Thus, one can conclude that the DRC can, when orthographic lexicon and grapheme-to-phoneme route are both implemented in lvOT, explain most of the pattern found in the lvOT by one or the other route of the model. Still, when using the reaction times from the model, i.e., the simulations using both routes, the expected contrast differences could not be simulated better than the orthographic lexicon results. Nonetheless, the simulated activation for pseudohomophones was lower than one would expect from the literature pattern. I.e., simulated activations were lower than words, pseudowords, and consonant stings in both routes.
