# Supplementary material for: The lexical categorization model: A computational model of left ventral occipito-temporal cortex activation in visual word recognition
Source: PLoS Comput Biol. 2022 Jun 9;18(6):e1009995. doi: 10.1371/journal.pcbi.1009995 (PMC9182256; doi:10.1371/journal.pcbi.1009995)
Supplement: S2 Fig — Significant correlation results between BOLD signals and parameters from the IA (upper row) and E&E (bottom row) simulations modeled as a single, continuous predictor for the data from Experiment 3. Thresholds for all whole-brain analyses: voxel-level: p < .001 uncorrected (cluster-forming); cluster level: p < .05 family-wise error corrected. No other regions than those displayed were significant. (DOCX) [file pcbi.1009995.s003.docx]

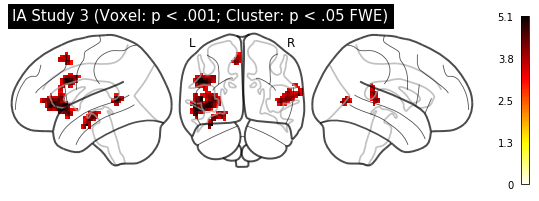

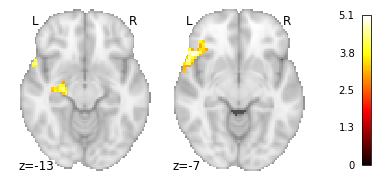


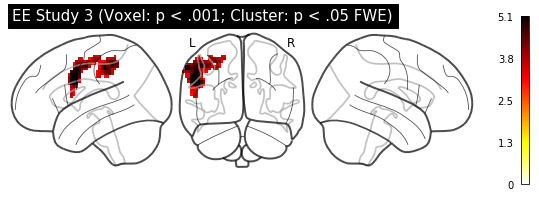

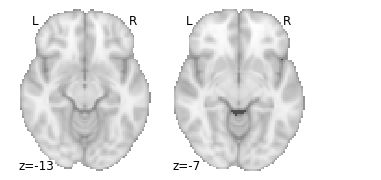


S2 Fig. fMRI whole-brain analyses based on the parameters from the Interactive account (IA) and engagement and effort model (E&E). Significant correlation results between BOLD signals and parameters from the IA (upper row) and E&E (bottom row) simulations modeled as a single, continuous predictor for the data from Experiment 3. Thresholds for all whole-brain analyses: voxel-level: p < .001 uncorrected (cluster-forming); cluster level: p < .05 family-wise error corrected. No other regions than those displayed were significant.
