# Supplementary material for: The lexical categorization model: A computational model of left ventral occipito-temporal cortex activation in visual word recognition
Source: PLoS Comput Biol. 2022 Jun 9;18(6):e1009995. doi: 10.1371/journal.pcbi.1009995 (PMC9182256; doi:10.1371/journal.pcbi.1009995)
Supplement: S3 Fig — In the main text, we reported an effect of word likeness in occipito-temporal regions posterior to the word-sensitive lvOT cluster in our second and third fMRI experiment (event-related single-trial design; Fig 3A and 3E). While the blocked design of fMRI experiment 1 was not primarily designed to demonstrate such stimulus-specific effects, we nevertheless also subjected this data set to an event-related analysis of word-likeness. Word-likeness, modeled as a continues factor, produced a more widespread activation effect in fMRI study 1, distributed over occipital regions of left and right hemisphere, with greater activity for more word-like letter strings (two significant clusters: Cluster 1: peak voxel at x = -12, y = -73, z = 1; Left lingual gyrus; T = 7.34; 514 voxels; Cluster 2: peak voxel at x = -6, y = -88, z = 37; Left cuneus; T = 4.0; 67 voxels). From the ventral view of the left hemisphere, it is visible that the cluster extended into the posterior lvOT, which is not the case in the right hemisphere (Activation effects are visualized at voxel level p < .001 uncorrected; cluster level p < .05 family-wise error corrected). In addition, we tested the words > pseudowords contrast: no significant activation difference between words and pseudowords was found. Only when neglecting the cluster correction, a small activation cluster was found in left frontal cortex (x = -39, y = 38, z = 25; Left frontal pole; T = 3.7; 7 voxel). To summarize, consistent with the second fMRI experiment, an effect of word-likeness on brain activation was found posterior to lvOT, while the (weak) lexicality effect was observed anterior to lvOT, i.e., in downstream regions of the frontal lobe. (DOCX) [file pcbi.1009995.s004.docx]

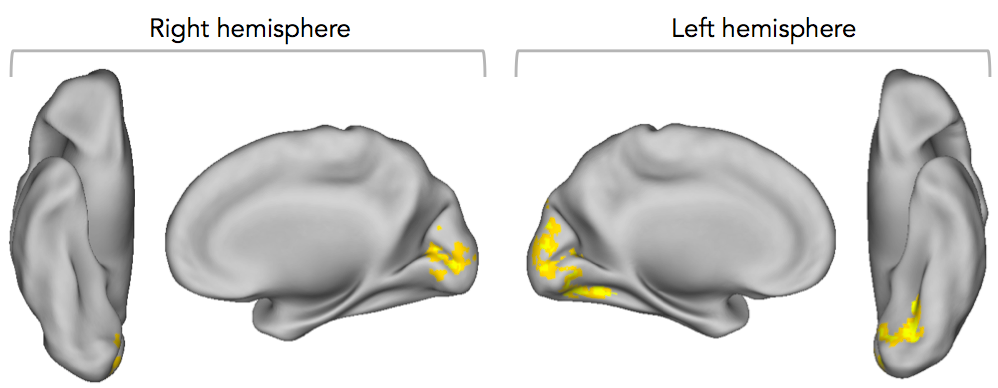
 *S3 Fig.* Word-likeness and lexicality effects for fMRI based Experiment 1. In the main text, we reported an effect of word likeness in occipito-temporal regions posterior to the word-sensitive lvOT cluster in our second and third fMRI experiment (event-related single-trial design; Fig 3A and 3E). While the blocked design of fMRI experiment 1 was not primarily designed to demonstrate such stimulus-specific effects, we nevertheless also subjected this data set to an event-related analysis of word-likeness. Word-likeness, modeled as a continues factor, produced a more widespread activation effect in fMRI study 1, distributed over occipital regions of left and right hemisphere, with greater activity for more word-like letter strings (two significant clusters: Cluster 1: peak voxel at x = -12, y = -73, z = 1; Left lingual gyrus; T =7.34; 514 voxels; Cluster 2: peak voxel at x = -6, y = -88, z = 37; Left cuneus; T = 4.0; 67 voxels). From the ventral view of the left hemisphere, it is visible that the cluster extended into the posterior lvOT, which is not the case in the right hemisphere (Activation effects are visualized at voxel level *p* < .001 uncorrected; cluster level *p*< .05 family-wise error corrected). In addition, we tested the words > pseudowords contrast: no significant activation difference between words and pseudowords was found. Only when neglecting the cluster correction, a small activation cluster was found in left frontal cortex (x = -39, y = 38, z = 25; Left frontal pole; T = 3.7; 7 voxel). To summarize, consistent with the second fMRI experiment, an effect of word-likeness on brain activation was found posterior to lvOT, while the (weak) lexicality effect was observed anterior to lvOT, i.e., in downstream regions of the frontal lobe.
