# Supplementary material for: The lexical categorization model: A computational model of left ventral occipito-temporal cortex activation in visual word recognition
Source: PLoS Comput Biol. 2022 Jun 9;18(6):e1009995. doi: 10.1371/journal.pcbi.1009995 (PMC9182256; doi:10.1371/journal.pcbi.1009995)
Supplement: S4 Fig — In the main text, we report an implementation of the LCM using OLD20 (29) as a measure of word likeness that has been reported in the literature to outperform other measures of word likeness (29). However, it is also possible to implement the LCM based on alternative measures of word-likeness. Here, we report three simulations of the benchmark effects tested in Evaluation 1 (cf. Fig 2), using (a) Coltheart’s neighbors, (b) trigram frequency, and (c) quadrigram frequency, as bases for the LCM simulations. The left-most columns show the distributions of the respective word-likeness measure for different types of letter strings as well as the probabilities of being a word or not and the resulting entropy (categorization uncertainty), analogous to Fig 1 in the main text. It is visible that all three measures are less well able to distinguish between words, pseudowords, and consonant strings than OLD20 (Fig 1A) does. As a result, the resulting entropy function has a different shape than the one derived from OLD20. The LCM implementation based on OLD20 (Figs 1 and 2) clearly outperformed (in terms of correctly predicted effects and estimated effect sizes) these models based on alternative word-likeness measures. When inspecting the pseudoword > words (4) contrast, only the model based on Coltheart’s N (S5A Fig) was able to predict this difference; on the other hand, this was the only model that did not predict the contrast words > consonant strings (2). For description of labels see Figs 1 and 2. (DOCX) [file pcbi.1009995.s005.docx]

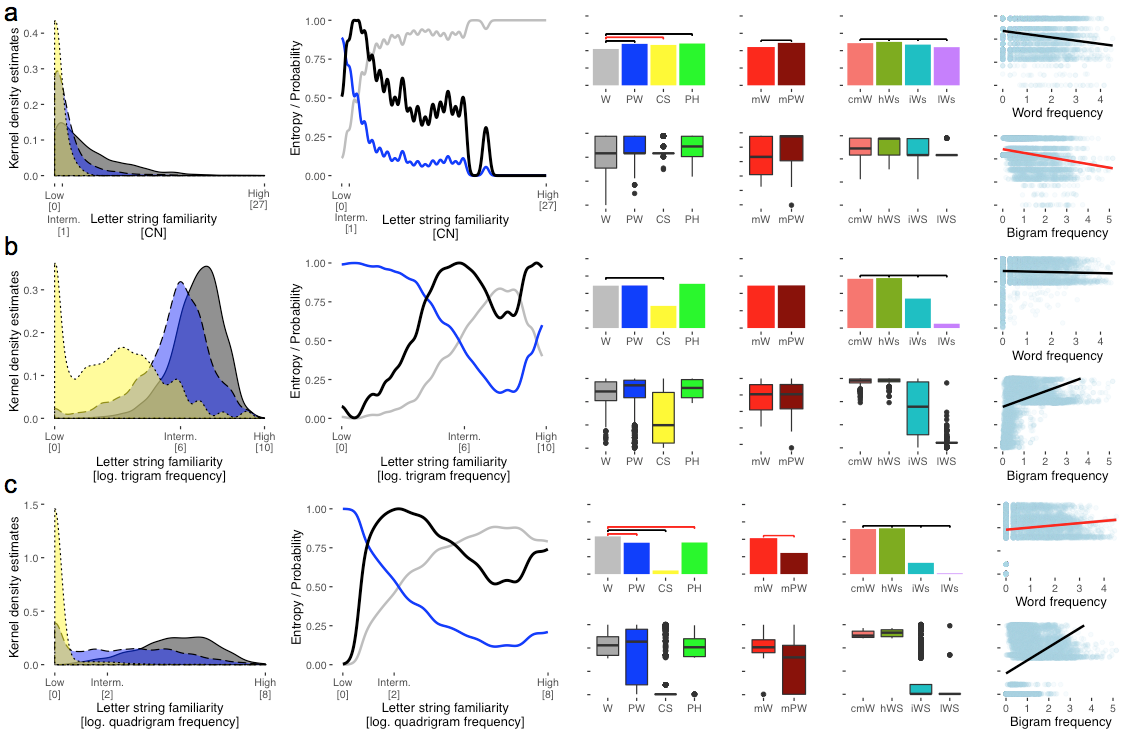


*S4 Fig.* LCM implementations and simulations based on three alternative word-likeness measures. In the main text, we report an implementation of the LCM using OLD20 (29) as a measure of word likeness that has been reported in the literature to outperform other measures of word likeness (29). However, it is also possible to implement the LCM based on alternative measures of word-likeness. Here, we report three simulations of the benchmark effects tested in Evaluation 1 (cf. Fig 2), using (a) Coltheart’s neighbors, (b) trigram frequency, and (c) quadrigram frequency, as bases for the LCM simulations. The left-most columns show the distributions of the respective word-likeness measure for different types of letter strings as well as the probabilities of being a word or not and the resulting entropy (categorization uncertainty), analogous to Fig 1 in the main text. It is visible that all three measures are less well able to distinguish between words, pseudowords, and consonant strings than OLD20 (Fig 1A) does. As a result, the resulting entropy function has a different shape than the one derived from OLD20. The LCM implementation based on OLD20 (Figs 1 and 2) clearly outperformed (in terms of correctly predicted effects and estimated effect sizes) these models based on alternative word-likeness measures. When inspecting the pseudoword > words (4) contrast, only the model based on Coltheart’s N (Suppl. Fig. 5a) was able to predict this difference; on the other hand, this was the only model that did not predict the contrast words > consonant strings (2). For description of labels see Figs 1 and 2.
