# Supplementary material for: The lexical categorization model: A computational model of left ventral occipito-temporal cortex activation in visual word recognition
Source: PLoS Comput Biol. 2022 Jun 9;18(6):e1009995. doi: 10.1371/journal.pcbi.1009995 (PMC9182256; doi:10.1371/journal.pcbi.1009995)
Supplement: S5 Fig — We assumed that lexicon size influences word-likeness estimations (i.e. the number of items in the lexicon to which e.g. the OLD20 is estimated) and LCM simulations. First, word-likeness distributions for words, pseudowords, and consonant strings, which were used for the LCM model (see Figs 1 and 2; see Materials section), are presented for lexica consisting of the most frequent 100, 1,000, 10,000 and 100,000 words of the SUBTLEX database. When comparing the word-likeness distributions, it becomes obvious that increasing the size of the lexicon results in a better differentiation between letter string categories (e.g., stronger differentiation between words and consonant strings). Simulations from LCM models derived from these distributions (compare to Figs 1 and S5), in the lower panels (line graphs show median LCM simulated activation), showed that the model predicted no difference between categories with very small lexicons. Lexicons with intermediate size already allow a differentiation between consonant strings (yellow) and the other stimulus categories. Starting from lexicons with 10,000 words, clearer differentiation between words (gray) and pseudowords (blue)/pseudohomophones (green) was present. In part, besides established effects such as acquired letter knowledge or grapheme to phoneme conversion (for example (4)), these simulations demonstrate that the increasing lexicon size may account for critical patterns of developmental change during literacy acquisition; our present work, in this context, suggests that the lvOT may be an important mediator of such developmental processes. (DOCX) [file pcbi.1009995.s006.docx]

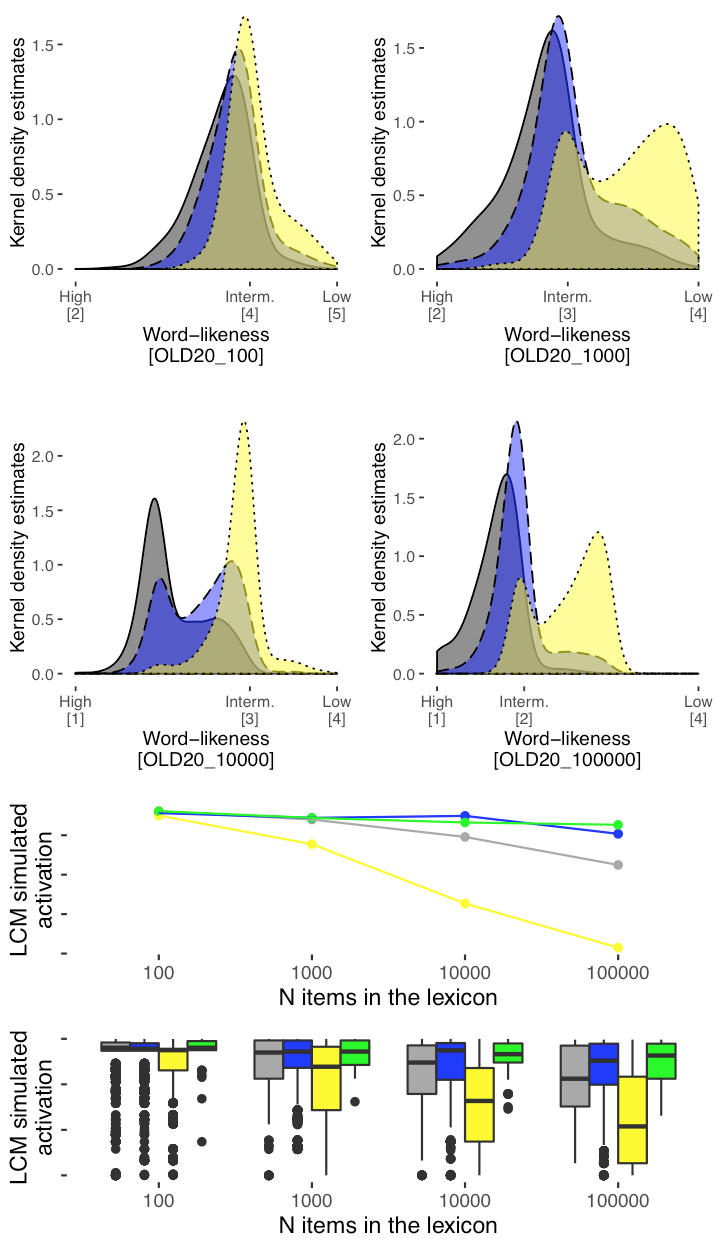


*S5 Fig*. Effect of lexicon size on word-likeness estimations and LCM simulations. We assumed that lexicon size influences word-likeness estimations (i.e. the number of items in the lexicon to which e.g. the OLD20 is estimated) and LCM simulations. First, word-likeness distributions for words, pseudowords, and consonant strings, which were used for the LCM model (see Figs 1 and 2; see Materials section), are presented for lexica consisting of the most frequent 100, 1,000, 10,000 and 100,000 words of the SUBTLEX database. When comparing the word-likeness distributions, it becomes obvious that increasing the size of the lexicon results in a better differentiation between letter string categories (e.g., stronger differentiation between words and consonant strings). Simulations from LCM models derived from these distributions (compare to Figs 1 and S4), in the lower panels (line graphs show median LCM simulated activation), showed that the model predicted no difference between categories with very small lexicons. Lexicons with intermediate size already allow a differentiation between consonant strings (yellow) and the other stimulus categories. Starting from lexicons with 10,000 words, clearer differentiation between words (gray) and pseudowords (blue)/pseudohomophones (green) was present. In part, besides established effects such as acquired letter knowledge or grapheme to phoneme conversion (for example(4)), these simulations demonstrate that the increasing lexicon size may account for critical patterns of developmental change during literacy acquisition; our present work, in this context, suggests that the lvOT may be an important mediator of such developmental processes.
