# Supplementary material for: The lexical categorization model: A computational model of left ventral occipito-temporal cortex activation in visual word recognition
Source: PLoS Comput Biol. 2022 Jun 9;18(6):e1009995. doi: 10.1371/journal.pcbi.1009995 (PMC9182256; doi:10.1371/journal.pcbi.1009995)
Supplement: S6 Fig — Upper section includes the mean correlation over twenty entropy correlations based on different randomly drawn sets of orthographic stimuli. Note, the proportion of words, pseudowords and consonant clusters was always the same. Lower section shows the standard deviation across the twenty calculations. Data is shown in 1% steps. (DOCX) [file pcbi.1009995.s007.docx]

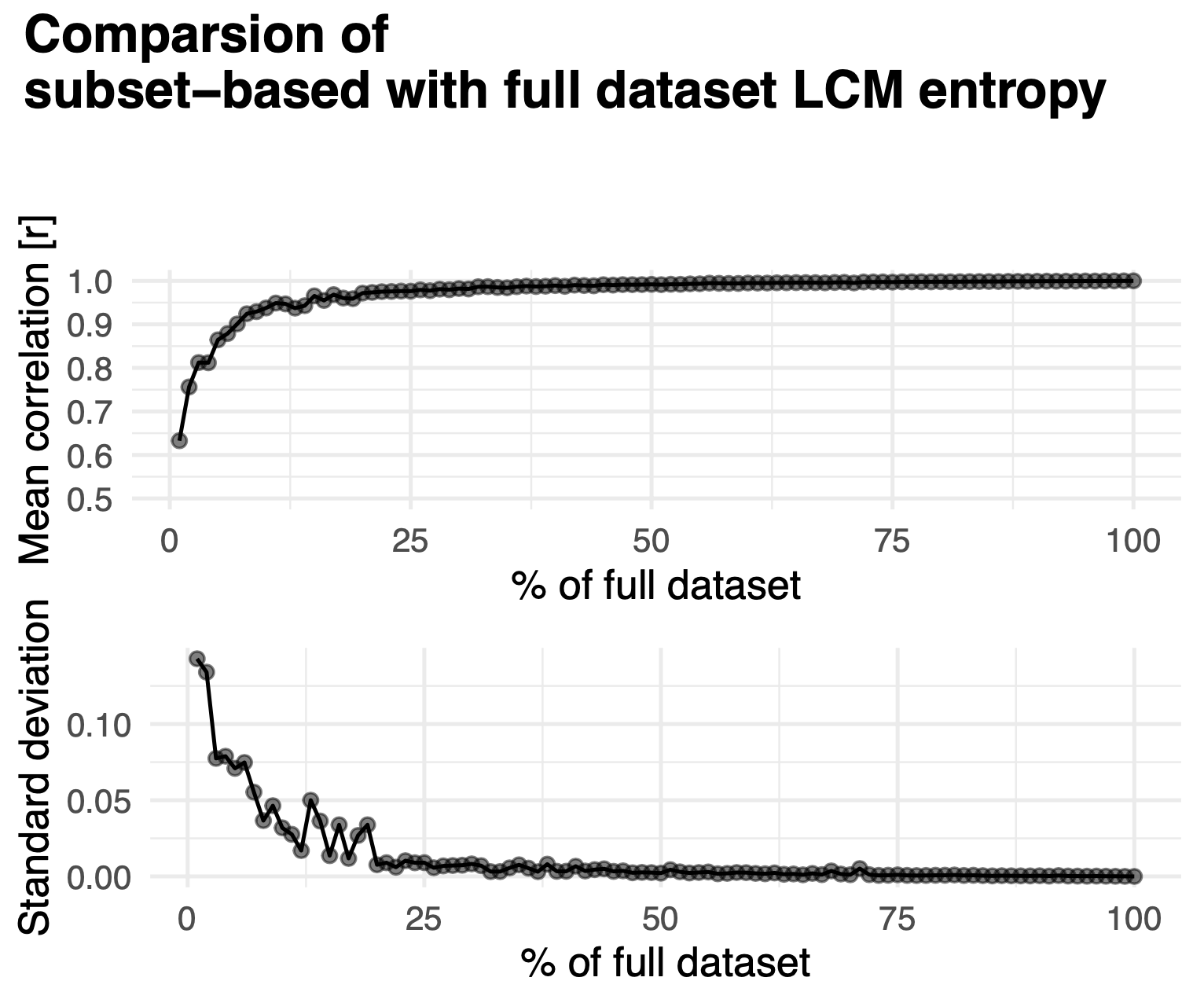


S6 Fig. Correlation of the LCM calculated entropy from subsets of the stimuli with the full set of the stimuli. Upper section includes the mean correlation over twenty entropy correlations based on different randomly drawn sets of orthographic stimuli. Note, the proportion of words, pseudowords and consonant clusters was always the same. Lower section shows the standard deviation across the twenty calculations. Data is shown in 1% steps.
