# Supplementary material for: The lexical categorization model: A computational model of left ventral occipito-temporal cortex activation in visual word recognition
Source: PLoS Comput Biol. 2022 Jun 9;18(6):e1009995. doi: 10.1371/journal.pcbi.1009995 (PMC9182256; doi:10.1371/journal.pcbi.1009995)
Supplement: S1 Methods — (DOCX) [file pcbi.1009995.s008.docx]

S1 Methods. fMRI measurement parameters

Experiment 1.

A Siemens Magnetom TRIO 3-Tesla scanner (Siemens AG, Erlangen, Germany) equipped with a 12-channel head-coil was used for functional and anatomical image acquisition. The BOLD signal was acquired with a T2*-weighted gradient echo-planar imaging (EPI) sequence (TR = 2250 ms; TE = 30 ms; Flip angle = 70°, 64 x 64 matrix; FoV = 210 mm). Thirty-six descending axial slices with a slice thickness of 3 mm and a slice gap of 0.3 mm were acquired within the TR. In addition, for each participant a gradient echo field map (TR = 488 ms; TE 1 = 4.49 ms; TE 2 = 6.95 ms) and a high-resolution structural scan (T1-weighted MPRAGE sequence; 1 x 1 x 1.2 mm) was acquired. Stimulus presentation was implemented by an MR-compatible LCD screen (NordicNeuroLab, Bergen, Norway) with a refresh rate of 60 Hz and a resolution of 1024x768 pixels.

Experiment 2.

A Siemens Magnetom TRIO 3-Tesla scanner (Siemens AG, Erlangen, Germany) equipped with a 32-channel head-coil was used for functional and anatomical image acquisition. The BOLD signal was acquired with a T2*-weighted gradient echo-planar imaging (EPI) sequence (TR = 2250 ms; TE = 30 ms; Flip angle = 70°, 86 x 86 matrix; FoV = 192 mm). Thirty-six descending axial slices with a slice thickness of 3 mm and a slice gap of 0.3 mm were acquired within the TR. In addition, for each participant a gradient echo field map (TR = 488 ms; TE 1 = 4.49 ms; TE 2 = 6.95 ms) and a high-resolution structural scan (T1-weighted MPRAGE sequence; 1 x 1 x 1.2 mm) was acquired. Stimulus presentation was implemented by an MR-compatible LCD screen (NordicNeuroLab, Bergen, Norway) with a refresh rate of 60 Hz and a resolution of 1024x768 pixels.

Experiment 3.

A Siemens Magnetom TRIO 3-Tesla scanner (Siemens AG, Erlangen, Germany) equipped with an 8-channel head-coil was used for functional and anatomical image acquisition. The BOLD signal was acquired with a T2*-weighted gradient echo-planar imaging (EPI) sequence (TR = 2250 ms; TE = 30 ms; Flip angle = 90°; 64 x 64 matrix; FoV = 210 mm). Thirty-six descending axial slices with a slice thickness of 3 mm and a slice gap of 0.3 mm were acquired within the TR. In addition, for each participant a gradient echo field map (TR = 650 ms; TE 1 = 4.89 ms; TE 2 = 7.35 ms) and a high-resolution structural scan (T1-weighted MPRAGE sequence; Experiment 3: 1 x 1 x 1 mm) was acquired. Stimulus presentation was implemented by a Sanyo PLC-XP41-projector (SANYO Electric Co., Osaka City, Japan) with a refresh rate of 60 Hz and a resolution of 1024x768 pixels.
