# Supplementary material for: The lexical categorization model: A computational model of left ventral occipito-temporal cortex activation in visual word recognition
Source: PLoS Comput Biol. 2022 Jun 9;18(6):e1009995. doi: 10.1371/journal.pcbi.1009995 (PMC9182256; doi:10.1371/journal.pcbi.1009995)
Supplement: Attachment — Submitted filename: Response-letter-PLOS_compBio_R1.pdf [file pcbi.1009995.s009.pdf]

Department of Linguistics • University of Vienna

Dr. Benjamin Gagl

Sensengasse 3a
1090 Wien

benjamin.gagl@univie.ac.at

To Editors-in-Chief
Prof. Peters & Prof. Einhäuser
PLOS Computational Biology

Dear Prof. Peters, Dear Prof. Einhäuser,

We want to re-submit our manuscript "*The lexical categorization model: A computational model of left ventral occipito-temporal cortex activation in visual word recognition*", coauthored by Fabio Richlan, Philipp Ludersdorfer, Jona Sassenhagen, Susanne Eisenhauer, Klara Gregorova, Christian J. Fiebach, and myself to be considered for publication in *PLOS Computational Biology* (ID: PCOMPBIOL-D-21-00321).

First, we thank the editors for considering our original submission and inviting us to submit a revised version of the manuscript. The reviews were of high quality and helped us further improve the paper's quality. We address all reviewer concerns point by point. Please find our detailed responses below.

For convenience, we here highlight the main changes:

- We added two more analyses of fMRI experiment 3, investigating the correlation of brain activation with the two most promising alternative models. Interestingly, the whole brain result did not show any significant correlates of alternative model parameters with activation in the ventral occipito-temporal cortex. See Supplemental Figure 3 for details
- We extended the discussion part by multiple paragraphs containing issues like limitations, outlook, dyslexia, and brain localization.
- More extensive methodological description of the behavioral training study.
- New model stability estimation, based on randomly drawn subsets of orthographic items, showed that only 8 % of the items are sufficient to generate a model entropy that is correlated with > .9 with the entropy estimated of the unconstrained lexical categorization model (see supplemental Figure 7).

We think that the work we added while revising the manuscript helps to strengthen the reported results and to support our main conclusion from the present work additionally, i.e., our proposal of a lexical categorization process implemented in the word sensitive part of the left ventro-occipito temporal cortex in visual word recognition and reading. Also, we now introduce here a framework for explicit quantitative testing of alternative models against our proposal while previous models of the brain bases of visual word recognition have remained purely verbal in nature. As such, we are convinced that this work will stimulate further model-based research on the neurocognitive bases of visual word recognition processes.

We look forward to hearing back from you and thank you for your consideration.

Cordially,

Benjamin Gagl, on behalf of all co-authors.

Reviewer comments and responses

Reviewer #1

Gagl and colleagues present an elegant and simple computational model to explain response properties in regions of ventral occipitotemporal cortex that respond to words. Their "Lexical Categorization Model" is intuitively simple while also having great explanatory power. It is based on the concept of "word likeness" which they instantiate in a simple and well-defined computation. Gagl and colleagues take a field that generally relies on verbal descriptions of effects and builds the first fully computable model of lexical processing in VOTC. I believe that this paper defines a solid foundation for future work to build on. I would also like to commend the authors for making their simulation code and model fitting code publicly available: allowing other scientists to work with this model will amplify the impact of this work. However, there is one major technical issue that needs to be addressed and there are a series of theoretical points that I think require further thought and attention.

Sincerely,

Jason Yeatman

#Technical issue:
Was a whole-brain analysis conducted for all the different models? The results suggest that they searched for voxels that fit the LCM model and then model comparisons were conducted on this region. This analysis would bias results in favor of the LCM model - this is not a valid approach to model comparison. To perform an unbiased model comparison it would be important to establish an a priori localizer to define a region that would then be compared across the different models. Alternatively, a whole brain analysis could be run for each model, and the results could be reported (but not directly compared). It is likely that there isn't a single computation performed in VOTC and that different models might fit better for different regions. This alternative seems more likely than a single model that characterizes all of VOTC.

Response:

We agree with the reviewer that one could have been the impression that the analysis favors the LCM. In the revised version, we now add two new whole-brain analyses of the third fMRI experiment (Study 3), based on the simulations of the IA and the E&E models, as an extension to figure 3. We chose this alternative as we did not implement independent localizer scans in the study. We used the data from Study 3 for this analysis, as this study used stimulus material specifically chosen to distinguish between the cognitive processes implemented in the most promising models (which are capable of simulating most effects present in the literature). As suggested by the reviewer, if an alternative model describes a computation implemented elsewhere in vOT, this analysis should identify it. As the correlations among the parameters from the different models are low, we would in that case expect a neuroanatomical differentiation between the three models. The correlation analysis of model simulations and brain activation was implemented in the same way as the LCM-based analysis already presented (methods on p. 42).

From this analysis, the critical finding was that no voxels of the vOT correlated significantly with either IA or E&E model parameters (see Supplementary Fig. 3 and below). Also, the results of these two analyses identified different brain areas as correlating with the parameters from the different

models: The IA model correlates with left frontal and bilateral temporal regions (Figure, upper row). In contrast, the E&E model correlates with left peri-central regions. (For comparison, the analogous analysis for the LCM model is presented in Figures 3 and 4 and shows robust correlation effects in lvOT.)

The reviewer also discusses the likelihood that a single model can characterize the cognitive processes of all vOT. We agree with the reviewer that other factors and processes beyond those captured by the LCM model may contribute to the processes that lvOT contributes to word recognition. Given the currently available data and models, we think that the additional results add evidence to our conclusion that the LCM, out of the currently available models, at present provides the best quantitative description of lvOT activation in response to words and non-words. The model fit is not perfect, so there might be different explanations with better results, but the LCM-based stimulus selection used in the fMRI experiment 3 and the LCM motivated training study are two pieces of evidence that indicate potential causality. Lastly, we agree with the reviewer that lvOT may implement different computations, not just the process proposed here for word recognition. Nevertheless, we think that the quantitative approach taken here for word-specific processes makes it possible to investigate in a quantitatively more explicit manner the relationships between different cognitive processes involved in word recognition and other aspects of high-level vision implemented in the lvOT.

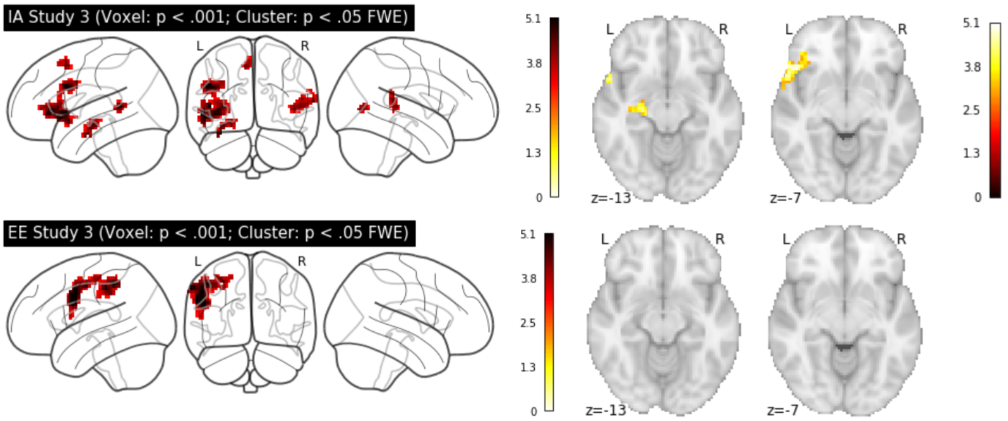

Supplemental figure 3. IA (upper row) and E&E model evaluation (bottom row): fMRI whole-brain analyses. Significant correlation results between BOLD signals and parameters from the IA and E&E simulations modeled as a single, continuous predictor in Study 3. Thresholds for all whole-brain analyses: voxel-level: $p < .001$ uncorrected (cluster-forming); cluster level: $p < .05$ family-wise error corrected. No other regions than those displayed were significant.

#Conceptual issue 1:

There is one major conceptual clarification that should be made up front. The goal of the LCM is to explain the processing of orthographic stimuli in VOTC. This is very different from a general purpose model to explain the wide array of response properties reported in VOTC. For example, the response to "false fonts" or foreign character strings or line drawings is nearly as high as the response to words and there is still a moderate response to checker boards, faces, objects and other visual stimuli in VOTC. There are also neighboring regions that prefer these other stimuli. The LCM model only explains the incremental differences in response to different orthographic stimuli and is not computable based on the wide range of other visual stimuli that have been used to probe

VOTC responses. This is a major distinction between the LCM and the fully computable model of VOTC developed by Kay and Yeatman 2017. In contrast, the sequence of models developed by Kay and Yeatman 2017 sought to characterize the general response pattern of different category selective VOTC regions but did not address the effects of different orthographic and lexical properties. I think it would be interesting to consider whether these two models might represent different stages of processing in word-selective cortex. For example, the template model from Kay and Yeatman might be implemented in an earlier (e.g. VWFA-1 or OWFA) word-selective region that feeds into a later (e.g. VWFA-2) word-selective region that computes the LCM. Discussing these different approaches would be helpful and, at the very least, clearly defining the goal of modeling response differences to orthographic stimuli as opposed to a fully-computable, image-based model would be a useful distinction.

Response:

This is a great point, as it fully aligns with our thinking about the modeling of lvOT responses. As the reviewer correctly points out, our motivation for proposing the LCM was to go one step beyond existing work and investigate processes implemented in vOT that come into play after a visual stimulus was categorized as a letter string or, in other words, to model cognitive processes implemented within the brain region selectively activated by written stimuli. We have now added more detail concerning this point into the Introduction section:

"The LCM proposal is consistent with one of the core computational functions of the ventral visual stream, i.e., categorizing percepts into different categories of objects based on a hierarchy of spatially different cortical processing stages (Grill-Spector & Weiner, 2014). For example, animate vs. inanimate objects activate separable ventral stream regions, while spatially dissociable sub-regions represent different lower-level features, such as faces or eyes for the animate subcategory. This object categorizing architecture is also known to categorize words vs. non-orthographic objects efficiently (Ben-Shachar et al., 2007; Glezer & Riesenhuber, 2013; Hirshorn et al., 2016), and activation in these regions correlates with lexical decision performance (Agrawal et al., 2020). Within the framework of hierarchically organized ventral stream processing, we thus assume that a categorical distinction between known and unknown orthographic percepts is computed after a lower-level categorization of sensory information into orthographic vs. non-orthographic input has taken place. More domain-general computational work has characterized the functional role of category-specific response patterns in vOT (e.g., (Kay & Yeatman, 2017)). In contrast, we here aim to more specifically model the effects of orthographic and lexical stimulus properties on word-selective cortex. Whereas previous work indicates that the former categorization process (e.g., words vs. faces) is based on visually distinct features, the lexical categorization process postulated here is proposed to rely on category-specific (e.g., orthographic, lexical) information." p. 6

#Conceptual issue 2:

fMRI study 1 is a nice test of the model. However, a distinction should be made between a whole-brain analysis of data in template space and a model fit to responses from individually defined VWFA ROIs. The location of the VWFA varies dramatically across participants and the reported coordinates vary dramatically across studies. For example, it has become clear that there are, in fact, a sequence of spatially distinct word-selective regions with different functions: individual localizers are essential to accurately distinguish these regions. This is not a problem for the presented analyses per se, but should be considered in the interpretation. For example, see here the dramatic variability in reported "VWFA" coordinates across the literature (https://osf.io/hv3y2/) and see White et al. 2019 for a detailed, model-based characterization of computational differences in VWFA-1 and VWFA-2: White, Alex L., John Palmer, Geoffrey M.

Boynton, and Jason D. Yeatman. 2019. "Parallel Spatial Channels Converge at a Bottleneck in Anterior Word-Selective Cortex." Proceedings of the National Academy of Sciences of the United States of America 116 (20): 10087–96.

Response:

We fully agree with the comment regarding high inter-individual variability in localization of the word-selective activation. We had in our original submission already referred to the proposal of more posterior vs. more anterior subregions within word-selective cortex (citing a recent study by Lerma-Usabiaga et al. 2018). Given this reviewer comment, we discuss this proposal in more detail (also referring to the more recent paper by White et al., 2019) and discuss that our model-driven lvOT results, in the light of this posterior/anterior distinction should be interpreted as pre-lexical effects:

"*The LCM postulates that this results from similar orthographic-perceptual familiarity of words and non-words fed into (word-sensitive subregions of) lvOT from lower levels, making a more detailed analysis of the orthographic input necessary. In the LCM implementation, this is accounted for by the partly overlapping distributions of word-likeness (OLD20) of words and non-words. The additional processing required by letter strings with intermediate word-likeness results in increased lvOT activation and slower responses, a proposal consistent with seminal behavioral work which proposed spelling information as a potential source aiding categorization when word-likeness information is insufficient (Balota & Chumbley, 1984). This, in turn, is compatible with our observation from fMRI Study 3, in which we used stimulus materials that were particularly difficult to categorize (e.g., Consonant strings with a high word-likeness). In this experiment, we observed that a parietal network that likely represents a dorsal sensorimotor interface connecting auditory-phonological with articulatory representations (Hickok & Poeppel, 2007) co-activates with lvOT when lexical integration difficulty increases. This parietal co-activation, interestingly, also relates our results to the recent proposal of a posterior/anterior dissociation within the word-selective ventral occipito-temporal cortex (Lerma-Usabiaga et al., 2018; White et al., 2019): Whereas posterior lvOT (also referred to as VWFA-1 by (Lerma-Usabiaga et al., 2018; White et al., 2019)) has been characterized as implementing pre-lexical processing during visual word recognition and was shown to be structurally connected to the parietal cortex (Caffarra et al., 2021; Lerma-Usabiaga et al., 2018), the anterior lvOT (VWFA-2) shows selectivity to lexical stimulus properties (Lerma-Usabiaga et al., 2018; White et al., 2019). The LCM-correlate identified in lvOT in the present study (Study 1: y = -64; Study 3: y = -75) is located close to the posterior lvOT subregion (Ref. (Lerma-Usabiaga et al., 2018): y < -63; Ref. (White et al., 2019): y ~ -69), suggesting a primarily prelexical nature of the categorization process implemented in the LCM. A proposal that is in line with the functional profiles of reading-related brain regions identified with EcOG electrodes (Lochy et al., 2018; Woolnough et al., 2020). Note, however, that these group-level effects also need to be interpreted in light of substantial inter-individual differences in functional localization of word-selective activity (e.g., (Caffarra et al., 2021)), so that the association between LCM effects and posterior lvOT/VWFA-2 should be replicated based on individual functional localizers.*" p. 34

#Conceptual issue 3:

In line with the above comment of anatomical precision, sentences like "LCM provides a fair characterization of lvOT BOLD activation patterns during visual word recognition and that it predicts lvOT activity better than other current models of lvOT function", should be revised. The whole brain analysis shows that there are some voxels for which this statement is true. But the statement is not true about all of lvOT nor is it true about specific sub-regions of word-selective cortex for which the model does not explain certain effects. To be concrete, sentences like "In sum, empirical data from three fMRI studies support the proposal that lvOT implements a lexical categorization process that operates upon an estimate of word-likeness computed by hierarchically preceding brain regions." Should be revised to say something like "In sum, empirical data from

three fMRI studies support the proposal that THERE IS A REGION IN lvOT THAT implements a lexical categorization process that operates upon an estimate of word-likeness computed by hierarchically preceding brain regions."

Response:

We fully agree with the reviewer. This was not formulated optimally, and we have adjusted these sentences accordingly:

P. 21: "In summary, the fMRI data of Study 1 indicate that the LCM provides a fair characterization of BOLD activation patterns in several lvOT voxels during visual word recognition and that it predicts activity in these voxels better than other current models of lvOT function."

P. 22: "In sum, empirical data from three fMRI studies support the proposal that there is a region in lvOT that implements a lexical categorization process which operates upon an estimate of word-likeness computed by upstream, more posteriorly located brain regions."

We have also made sure that results are described more carefully throughout the remaining text upon preparing the revised manuscript.

Similarly, the first sentence of the discussion should not label this region the visual word form area without performing a localizer in individual brains. "In the vicinity of the visual word form area" would be a fair statement.

Response:

We have changed this sentence accordingly.

"The newly proposed LCM model postulates that word-sensitive lvOT areas in the vicinity of the functionally localized visual word form area implement a categorization process that classifies letter strings into meaningful (words) vs. meaningless input, preventing attempts to apply downstream lexical-semantic processing to non-linguistic input." p. 30

Fmri Study 2 was a clever manipulation with clear results. But, in line with my previous point, the claims should be made specifically about the region identified in the above group average analysis not about VOTC in general or the VWFA specifically.

Response:

We now provide a more accurate description of our findings regarding the location and labeling of the brain areas in question.

For example: " Model comparisons based on the word/pseudoword contrast showed that the LCM predicted the observed activation contrast best (i.e., <1 SD; Fig. 5n). To summarize, here we replicated

the superior prediction performance of the LCM model in an event-related design, even when no stimulus-specific difference was found in a ROI located in the lvOT. " (p. 22)

<u>#A couple general points about references:</u>

Binder and colleagues showed similar findings to reference 12 (Vinckier) and would be a good citation to add about word similarity. I believe this was the first paper to make the referenced point Binder, J. R., D. A. Medler, C. F. Westbury, E. Liebenthal, and L. Buchanan. 2006. "Tuning of the Human Left Fusiform Gyrus to Sublexical Orthographic Structure." NeuroImage 33 (2): 739–48.

<u>Response</u>:

Thanks for pointing this out and reminding us of this paper. We have included and cited this paper (Reference N.: 13) when referring to word similarity, e.g., on pages 4 and 12.

#Conceptual issue 4:

The paper doesn't confront the broader work on computations performed in ventral occipitotemporal cortex. Even though there has not been much modeling work done for word recognition, there is a wealth of work developing computational models of the response properties in visual cortex. There are other papers that propose models of VOTC that address the response to a broad range of visual stimuli (without a specific focus on the differences between different letter combinations). A comprehensive review of this literature is not necessary. But acknowledging how the present model fits within the broader literature would make this work broadly influential. For example, Doris Tsao's lab just published a paper in Nature presenting a new "object space" model. Our previous work developed a template model with top-down modulation from the IPS. Srihasam and colleagues posited a general architecture that predicts where word-selective regions should emerge in VOTC. Citations below:
Bao, Pinglei, Liang She, Mason Mcgill, and Doris Y. Tsao. 2020. "A Map of Object Space in Primate Inferotemporal Cortex." Nature, no. January 2019. https://doi.org/10.1038/s41586-020-2350-5.
Kay, Kendrick N., and Jason D. Yeatman. 2017. "Bottom-up and Top-down Computations in Word- and Face-Selective Cortex." eLife 6 (February). https://doi.org/10.7554/eLife.22341.
Srihasam, Krishna, Justin L. Vincent, and Margaret S. Livingstone. 2014. "Novel Domain Formation Reveals Proto-Architecture in Inferotemporal Cortex." Nature Neuroscience 17 (12): 1776–83.

<u>Response</u>:

We thank the reviewer for this point and now provide a better reference frame for the LCM in the manuscript. In our opinion, sorting models, i.e., a model ontology, is at the current state only possible on a general level (i.e., similar as in Kay, 2018). For that reason, we now added a paragraph to the

discussion that relates current modeling approaches of IT cortex to each other. Predominantly, Deep Neuronal Network (DNN) models have been used so far, but also more transparent/explainable approaches have been used in object recognition, like the *Template model* model by Kay & Yeatman, 2017. To account for the comment, we add the following paragraph to the paper's discussion section. In this paragraph, we stress that the nature of a model on the axis of model transparency/explainablility could be a helpful reference frame.

" *Various computational models have described cognitive processes in higher vision implemented in the vOT. These models are concerned with visual word recognition (e.g., (Kay & Yeatman, 2017; Rajalingham et al., 2020)), object or face recognition (e.g., (Bao et al., 2020; Kar et al., 2019; Srihasam et al., 2014)). Current computational cognitive model approaches are often based on deep neuronal network models (DNNs) that can investigate complex system behavior and differences in computational architectures (Cichy & Kaiser, 2019; Ma & Peters, 2020). Also, these models achieve highly accurate predictions for vOT activation patterns during object recognition (e.g., (Güçlü & Gerven, 2015)). Still, DNNs typically include numerous free parameters fitted in a specific training setup, i.e., typically in an image classification task. This characteristic of DNNs comes at the cost of reduced model transparency and explainability (e.g., (Cichy & Kaiser, 2019; Kay, 2018)). More transparent approaches, like the Template model used by Yeatman and Kay (Kay & Yeatman, 2017), have a similar scope, i.e., an image input and a categorical output (word vs. face), but achieve this with only a limited number of free parameters (N=3). The model described here shares this aspect of greater transparency and explainability. Developing the LCM was under the premise that the model should be as simple as possible in the implementation and as deterministic as possible (i.e., with a minimal number of free parameters). Given that the LCM model output only depends on the words stored in the lexicon, it is deterministic in the sense of not including any free parameters and thus also fully transparent. However, the current implementation of LCM lacks critical model infrastructure, i.e., an interface for image inputs or a decision algorithm."* p. 35

Reviewer #2:

#Issue 1:

I'm not specialized in practice on computational model and MRI data processing, but both the method and results sections are quite clear and comprehensive. The introduction might be improved by adding some modeling / method comparison to help the reader clearly identify the objectives and originality of the study.

Response:

Thank you for pointing out this potential shortcoming. Given that we quite explicitly introduced several competing models from the word recognition literature and their assumptions (i.e., the local combination detector model, the lexicon model, the engagement and effort model, and Devlin and Price's interactive account; cf. p. 5, paragraph 2), we interpret this comment as referring more generally to the methodological approach of model-driven fMRI analysis. Please note that these models have so far remained verbal-descriptive,i.e., they have not been computationally implemented, so that quantitative hypotheses cannot be derived. However, model falsification can be implemented more optimally when based on quantitatively explicit models and predictions. The importance of computationally implemented hypotheses and models for characterizing brain responses to cognitive challenges has been highlighted repeatedly in the last years (e.g., Forstmann, B. U., & Wagenmakers, E. J., 2015). We think that the present work's originality arises from the objective of an explicit model comparison using model-based fMRI, which has been lacking in the study of lvOT involvement in visual word recognition. To highlight this more strongly, we have made the following changes to the introduction section:

"... Non-linear models include the engagement and effort (E&E) model (Taylor et al., 2013) and the interactive account (IA) model (Price & Devlin, 2011). The E&E model assumes that the lvOT activation reflects the combination of the engagement and effort related to an orthographic stimulus. Engagement reflects that an illegal letter string (i.e., highly unfamiliar letter combinations) is less likely to be processed by a word recognition area when compared to a known word, so that brain activation is expected to be lower for the illegal string. Effort, in contrast, reflects that lvOT activation is greater for less often processed words than for frequently encountered words (Taylor et al., 2013). The IA model assumes orthographic processing based on the principles of predictive coding (Rao & Ballard, 1999) based orthographic processing, such that lvOT activation reflects the error associated with internally generated predictions about the upcoming word (Price & Devlin, 2011). The non-linearity of the IA model comes from the assumption that consonant strings do activate lvOT to a lesser extent since for these letter strings, few top-down predictions can be formed (e.g., in the context of an experimental task), which results in a low prediction error. In contrast, rare words are less often expected and thus often violate current predictions, so that the prediction error (and consequently the activation of lvOT), is high. Frequent words, on the other hand, can be predicted better so that both the prediction error and lvOT activation are lower for high- than for low-frequent words. None of these models of lvOT activation during word recognition have been implemented yet, so that explicit model comparisons are missing. Also, these alternative non-linear models can likely capture the response profile of lvOT activation to some extent but are conceptually harder to integrate into the predominant categorization-based accounts present for object recognition (e.g., (Grill-Spector & Weiner, 2014) and see above) than the LCM.

Here, we evaluate the LCM against ad hoc implementations of the four alternative models introduced above and against a widely acknowledged, implemented cognitive model of visual word recognition, the Dual Route model (Coltheart et al., 2001) (which is in part a connectionist model). These evaluations focus primarily on comparing simulated vs. empirically observed lvOT responses to different stimulus characteristics – by generating simulated brain activations of observed activation patterns from different models (model-based fMRI analysis; see, e.g., (Forstmann & Wagenmakers, 2015)). First, models are compared based on their ability to simulate nine benchmark effects reported in the literature (Evaluation based on simulations). Second, we report empirical evaluations based on new fMRI studies that localize a neural correlate of the lexical categorization computation. After that, we compare models based on how well their simulations fit to the observed patterns of lvOT activatio (fMRI-based Evaluation). Finally, we investigate if training the core computation assumed in the LCM, i.e., the process of lexical categorization, increases reading efficiency (Training-based Evaluation). "
p. 7

#Issue 2:

In the introduction, the authors wrote that empirical data indicate that word similarity increases lvOT activation and word-familiarity decreases lvOT activation. It would be interesting to present data of morphological and both morpho-semantic and morpho-orthographic processing since word similarity is related to sub-lexical orthographic similarity (and also morpho-orthographic processing) and word similarity is related to word frequency (and also morpho-semantic processing).

Response:

Morphemes are not investigated yet, as they are word characteristics that have not been in the focus while developing the LCM model. In the appendix (Supplementary Figure 5) of the paper, we compare the LCM performance based on alternatives to the OLD20 word characteristic (i.e., Coltheart N, tri- or quadrigram-frequency) and observe that the OLD20-based version of the LCM results in the most accurate simulation results. Without any doubt, representational qualities related to morphemes are

fascinating in future research. We now added a paragraph to the discussion indicating the importance of morphemes. Please find this paragraph below in our response to Issue 3.

Also, some papers using representational similarity analysis (but also other analyses) have showed differences in the degree of activation along the lvOT cortex – left ventral inferior temporal cortex which is dependent to the level of stimuli and processing (posterior part more low level and anterior part more high level). If relevant, a discussion would be interesting.

Response:

Unless we misunderstand this proposal, representational similarity analysis is not applicable because the model implementations do not have variance. I.e., every simulation run related to a specific word would end up in the same number. However, this variance would be needed to implement a representational similarity analysis. We also think that RSA is not necessary for the present study because we show that simulated activations from the LCM directly predict activity in a subfield of lvOT. Please note that inspired by a comment of Reviewer 1, we now also conducted whole-brain model-based fMRI analyses based on parameters from competing models, and none of those was predictive of lvOT activity. For this reason, even though RSA is an intriguing method for relating representational spaces of models to brain activation data, we report even more direct associations between models and fMRI activations.

#Issue 3:

The discussion should be better organized, especially the first paragraph which should present the objectives and results summary. Overall, I find the discussion quite short about the perspectives of application of this study and limitations. How these findings can be used by researchers or future studies? Also, a discussion about how this finding can be compared with connexionist approach would be very interesting in a theoretical perspective.

Response:

We now restructured the first paragraph of the discussion section. Please find the new version below. We also included several further new paragraphs into the discussion section (i.e., on Individual differences, lvOT location, or limitations and outlook) so that we hope the length of the discussion is now more applicable. We added a section discussing possible limitations and an outlook on potential future work following the present report (see below). Concerning the proposal of comparing the LCM findings to a connectionist model, we would like to remind the reviewer that we report an explicit model comparison between the LCM and the dual-route model. In that model, the grapheme-to-phoneme route is characterized as a connectionist architecture.

Initial discussion paragraph:

"*In the work reported here, we used model-based fMRI analysis to clarify the nature of computations implemented in (subregions of) left-ventral occipitotemporal cortex (lvOT) in response to orthographic percepts (letter strings). To achieve this, we implemented the four most prevalent theories of lvOT function during language processing (e.g., (Dehaene et al., 2005)), the most widely accepted*

psychological model of visual word recognition (i.e., the Dual Route Model; (Coltheart et al., 2001)), and a new hypothesis, the lexical categorization model (LCM), as computationally explicit models and performed direct model comparisons using different types of data. The newly proposed LCM model postulates that word-sensitive lvOT areas in the vicinity of the functionally localized visual word form area implement a categorization process that classifies letter strings into meaningful (words) vs. meaningless input, preventing attempts to apply downstream lexical-semantic processing to non-linguistic input. The LCM implementation makes explicit how the lexical categorization process is related to neural processing, i.e., via the estimated difficulty of categorizing a letter string.

We compared the implementations of the LCM and alternative models directly by simulating established effects from previous fMRI studies. Through its lexical categorization mechanism, the LCM successfully accounts for the intricate pattern of lvOT activation in response to a variety of different (sets of) orthographic stimuli, irrespective of their specific task context (compare, e.g., (Kronbichler et al., 2004; Vinckier et al., 2007)). Most notably, the LCM model can simulate multiple published fMRI findings that have been viewed as contradictory, and it does so better than competing models. In addition, we found that alternative models implementing processes that in principle allow for non-linear activation patterns (IA model, (Price & Devlin, 2011); E&E model, (Taylor et al., 2013)) performed better than linear models like the local combination detector model (e.g., (Dehaene et al., 2005)). Furthermore, we demonstrate that the LCM reliably predicts empirically measured BOLD fMRI activations in voxels of the mid-lvOT and outperforms alternative non-linear models in three experiments that differed concerning the selection and composition of stimulus sets (in both whole-brain and peak-voxel defined ROI analyses). We observed that word-likeness, i.e., the quantitative psycholinguistic property on which the LCM's lexical categorization process is based, is predominantly represented in regions posterior to the brain regions showing a LCM effect. In contrast, a dichotomous lexicality effect with increased activity for words is implemented in brain regions anterior to the lvOT subregion associated with the LCM effect. These findings are consistent with the proposal that lvOT's categorization process gates known words into subsequent stages of lexical processing (see also (Lerma-Usabiaga et al., 2018)).

Finally, we demonstrate that repetitive behavioral training based on lexical decisions, which involve lexical categorization as a core process, significantly increases reading speed. Given that reading speed as tested in this study does not explicitly involve word/nonword decisions, we argue that this training study can provide causal evidence for an involvement of lexical categorization in the process of reading. Overall, these findings suggest that during reading, following the initial stages of visual-perceptual processing, posterior brain regions compute an estimate of the word-likeness (relative to the known lexicon of words). According to the LCM model, this word-likeness estimate is fed forward to a lexical categorization process implemented in a subregion of lvOT, which in turn precedes the extraction of word meaning (lexical access) at downstream cortical sites, including more anterior temporal and frontal cortex of the left hemisphere (Fig. 7)." p.30

Discussion paragraph on further research.

"The current implementation of the LCM model is limited by the fact that it cannot, at present, account for individual differences in reading abilities, reading experience, or knowledge of words. These limitations, however, are not principle limitations but a pragmatic decision to demonstrate the general feasibility and predictive value of the proposed lexical categorization process. Fitting the model to actual participants' performance will require estimating parameters related to lexicon size, as these are the basis for deriving the word similarity parameter that is the basis for the lexical categorization process. Also, our current work was restricted to the German language. However, there are no principled reasons

why the proposed lexical categorization process should apply more to (the orthography of) one language than others. Thus, future work should also explore the LCM model in other languages and orthographies (e.g., see Gagl et al., 2021 for a potential use case). Lastly, the present work may also potentially be driven by the specific nature of pseudowords and non-words used in our work. However, during stimulus construction, we aimed to represent a wide variety of letter strings (concerning their word-likeness) so that the LCM evaluations reported here should be broadly generalizable. On the other hand, future work could test various types of pseudo- or non-words that, e.g., differ on specific word characteristics like morpheme structure."

#Issue 4:

Does this model is sensitive to (modulated by) the reading related skills (word reading and pseudoword decoding skills, at least) of participants that were included in the fMRI experiment?

Response:

The LCM does not have a variance component representing individual differences between participants (see also our response above w.r.t. RSA). As an approximation, the current implementation is based on the assumption that the lexicon (and thus similarity among lexical entries, at present reflected by OLD20) is homogeneous across participants. Our application of the LCM model for analyzing training effects (Study 4) demonstrates that the model can be used to estimate how strongly a cognitive process, i.e., represented by model parameters, is involved in the reading process separately for every participant. To account for individual differences in reading skills as proposed by the reviewer, the size and contents of the lexicon (depending on the participant's individual learning history) and resulting word similarity parameters would have to be estimated individually. This adaptation is challenging and has been left out of the present report as we primarily focused on introducing this model and conducting fundamental comparisons to relevant competitor models. However, this is in principle possible, and we agree with the reviewer that this will be an important future application of the LCM model. In the revised manuscript, we now also discuss the potentials of individual-level modeling in the new limitations and outlook paragraph of the revised discussion section (see our response to your previous point).

#Issue 5:

Would the authors be more specific and detailed about the training experiment (Evaluation C)? As currently wrote it is very difficult to have a clear picture of the training content and procedure.

Response:

Thank you for pointing this out. We made efforts to describe the procedures of the training study in more detail. Please find the new version below and on p- 44 of the revised manuscript.

" Training-based Evaluation: 76 non-native speakers of German completed a three-day training procedure with one session of lexical categorization training per day (see Figure 6a). Reading skill was assessed before and after the training procedure using a frequently-used German reading speed screening procedure as a test of reading skill (Salzburger Lese Screening/SLS; Adult version of (Auer et al., 2005)). During the training sessions, words and non-words were presented in random order (presentation time until response), and participants had to indicate by button press whether or not the presented letter string was a word (lexical decision). After the button press, feedback on the correctness

of the response was given (200 ms; see Figure 6b), and the subsequent trial was presented after an inter-stimulus interval of 1,000 ms (during which a fixation cross was presented). In each session, 1,600 5-letter letter strings, including 800 words, 400 pseudowords, and 400 consonant strings, were presented (total length: about 50 min per training session). Stimuli were selected randomly from the letter strings included as the basis of the LCM (i.e., all lexicon words with five letters and non-words constructed from these words; see Figure 1a). The goal of the random selection was that the word/non-word characteristics, e.g., the word-likeness (OLD20) and word frequency, represent the naturally-occurring distributions in the lexicon to avoid artificial stimulus sets and allow participants to learn based on a representative set of words. Stimulus presentation was implemented with the Experiment Builder software (SR-Research, Ontario, Canada) using mono-spaced Courier-New font, with a visual angle of approximately 0.3° per letter. The presentation order of stimuli and experimental conditions was randomized for each participant and each of the three training sessions. For data analysis, we recorded response times and response accuracies. Analysis of behavioral training data was based on a two-step procedure. First, a linear mixed model was used to estimate the effects of LCM-based entropy, training session, OLD20, logarithmic frequency (per million from the SUBTLEX database), logarithmic stimulus presentation order, lexicality, and the correctness of the response on logarithmically transformed response times. For random effects, we followed the logic of parsimonious mixed models (Bates, Kliegl, et al., 2015) and ended up with a random effect structure that estimated the random effects of the intercepts of stimuli and participants. To investigate the strength of the individual interaction of the LC effect, i.e., the change of the LC effect with training, we estimated the random slope of this interaction term on the participants. Using the ranef() function of the lme4 package in the statistics package R, one can extract each participant's interaction effect. We used the individual interaction effects estimation for the GLM analysis that used the individual slope estimates from the linear mixed model as a regressor describing the pre-/post-reading speed assessment (see Figure 6f). For Figure 6e, we used the fitted LMM model to visualize the change in the LCM effect across sessions based on simulations using the remef package (Hohenstein & Kliegl, 2014/2017) that allows removing co-varying effects. " p.48

#Issue 5:

It would have been interesting to use Representational similarity analysis to model the fMRI data. I am not asking the authors to do it, but maybe it would be interesting to make some hypothesis on other type of analyses, especially because you are using correlation analysis (Figure3).

<u>Response</u>:

As outlined above (i.e., response to Issue #4), the current implementation of the LCM model avoids an issue that is typically the primary motivation for using representational similarity analyses (RSA): The classical RSA overcomes the problem that two responses to the same stimulus cannot be directly compared. For example, brain activity measured by multivariate methods using BOLD cannot be directly related to electrophysiological responses (e.g., see Cichy et al., 2014, Nature Neuroscience). In such cases, RSA can abstract from the constraints of the measurement methodology onto a representational correlation matrix that can be directly compared to correlation matrices derived from other sources, like a model of the representational space of interest. Here, however, we have a fundamentally different situation, as the output of the LCM is a simulated brain activation that can directly be compared to the measured fMRI BOLD response (as is also done with other model-based approaches, see, for example, the extensive discussion in the book by Forstmann & Wagenmakers, 2015). Put differently, we do not need to 'move' our analysis into the abstract representational space to compare the simulated with the measured brain activation. As said above, we think that predicting brain activity, per stimulus, directly from a model parameter, is strong support for correspondence between modeled processes and the actual neurocognitive processes implemented in that specific area. We fully agree that RSA is an intriguing method and have used it in other contexts (e.g., for comparing image

representations in Gagl et al., 2020). However, applying RSA in the present work would be a less direct approach to establish correspondence between the model and brain processes.

#Issue 6:

I understand it is not the topic of this study, but since the authors have started the introduction by presenting the limit of the studies conducted on lvOT, including the fact that this region is compromised in developmental reading disorders but that there is no agreed-upon mechanistic understanding of the specific processes lvOT contributes to visual word recognition, it would be interesting to provide a summary of this specific question, and also some hypotheses about reading disabilities.

Response:

We now add discussion on our hypotheses that arise from our finding that the LC algorithm is implemented in a region that shows altered activation patterns in dyslexia. Also, we suggest how to train a deficit that has been identified based on an individualized computational implementation of the LCM. Please find the sections below and on p. 34 of the revised manuscript.

" *The present results may also have implications for understanding dyslexia, i.e., a developmental deficit in reading performance. People who have dyslexia are characterized by altered structure and function in the subregion of lvOT that we related in the present work to the process of lexical categorization (Linkersdörfer et al., 2012; Richlan et al., 2009). Consequently, novel hypotheses concerning the nature of the deficit(s) underlying dyslexia might emerge from LCM-based studies of the process of lexical categorization or the word-similarity signal on which the lexical categorization process operates. Extending the LCM model to fit individual subjects' behavioral performance patterns would be interesting to test such hypotheses (i.e., as implemented here (Gagl et al., 2021)). Also, modeling subject-specific behavioral performance would provide a novel approach to quantitative, model-driven diagnostics from which personalized training schemes could be derived.*" p.36

Reviewer #3:

In this article, taking advantage of non-linear simulation based on information theory, authors challenged to explore the specific processes that the lvOT contributes to visual word recognition. They succeeded in explaining in a convincing manner the reason of superficial contradiction why word-similarity or sub-lexical orthographic similarity increases lvOT activation, where word-familiarity in the sense of word frequency decreases lvOT activation. I can underscore the research strategy of this article for substantive approach to the core of evaluating the neural activity as a non-linear function of orthographic structure. It is quite interesting to see that their methodology was accomplished by inventing a sound and quite ingenious simulation methodology. I quite agree with the authors that they could capture very well by the concept of entropy the pattern of lexical categorization difficulty.

Response:

Thank you very much for your positive feedback on our work.

#Issue 1:

Yet, in spite of their unimpeachable line of reasoning, it cannot be helped saying that this paper made a more or less messy impression. I strongly recommend that the authors devise nomenclature for brief representation of each particular experiment or methodology instead of mere numbering such as "Study 1" or "Evaluation C," once these notations appear alone like symbols. Since the organization of this paper is too complicated, I am afraid that the readers will feel difficulties in referring to the definitions and the contents only by casting a glance at these notations.

Response:

Thank you for this proposal. We now changed 'Evaluation A' to 'Evaluation based on simulations'; 'Evaluation B' to 'fMRI-based Evaluation', and 'Evaluation C' to 'Training-based Evaluation'. Within the section on the fMRI-based Evaluation, we differentiate between Experiments 1-3.

#Issue 2:

The explanation about each of the three fMRI experiments should be more concise in the Methods section (too long). This section is too labyrinthic to easily return to the main thread from each ramification. For example, readers will be wondering why the scan settings (even head coils were disparate) were so different across these three, because the experimenters did not need to overly adjust the purposes of the experiments to the minimal capacity of scanning conditions. It would be better to put these details to the supplementary documents to avoid apparent complication.

Response:

This point is well taken. These differences reflect that existing datasets from different laboratories were used for the initial model evaluations, while the third fMRI dataset was newly acquired. We think that this is generally an optimal approach in the sense of cost efficiency and research transparency. We have followed the recommendation and moved the information into the supplement. Most specifically:

1. We explicitly describe in the methods section that variance in acquisition parameters exists due to the use of datasets from different laboratories.
2. We have distilled shared information that is retained in the main text.
3. We have moved the description of specific parameter values that differ between fMRI experiments into the supplement.

Main text:
"fMRI data; Experiments 1, 2, and 3. The three fMRI datasets were measured in two different laboratories (Experiment 1 and 2: University of Salzburg; Experiment 3: Brain imaging Center, Goethe University Frankfurt) at three different time points. This resulted in variation of data acquisition parameters. For this reason, a detailed description of data acquisition parameters is

provided in Supplementary Methods 1. All experiments were measured using 3 Tesla Siemens Magnetom MR scanners, using BOLD sensitive T2-weighted gradient echo, echo-planar imaging (EPI) sequence, acquiring 36 axial slices at a TR of 2250 ms.*"p.49

Supplement:

"*Supplementary Methods 1. fMRI measurement parameters*

Experiment 1.
A Siemens Magnetom TRIO 3-Tesla scanner (Siemens AG, Erlangen, Germany) equipped with a 12-channel head-coil was used for functional and anatomical image acquisition. The BOLD signal was acquired with a T2-weighted gradient echo-planar imaging (EPI) sequence (TR = 2,250 ms; TE = 30 ms; Flip angle = 70°, 64 x 64 matrix; FoV = 210 mm). Thirty-six descending axial slices with a slice thickness of 3 mm and a slice gap of 0.3 mm were acquired within each TR. In addition, for each participant a gradient echo field map (TR = 488 ms; TE 1 = 4.49 ms; TE 2 = 6.95 ms) and a high-resolution structural scan (T1-weighted MPRAGE sequence; 1 x 1 x 1.2 mm) was acquired. Stimulus presentation was implemented by an MR-compatible LCD screen (NordicNeuroLab, Bergen, Norway) with a refresh rate of 60 Hz and a resolution of 1024 x 768 pixels.*

Experiment 2.
A Siemens Magnetom TRIO 3-Tesla scanner (Siemens AG, Erlangen, Germany) equipped with a 32-channel head-coil was used for functional and anatomical image acquisition. The BOLD signal was acquired with a T2-weighted gradient echo-planar imaging (EPI) sequence (TR = 2,250 ms; TE = 30 ms; Flip angle = 70°, 86 x 86 matrix; FoV = 192 mm). Thirty-six descending axial slices with a slice thickness of 3 mm and a slice gap of 0.3 mm were acquired within the TR. In addition, for each participant a gradient echo field map (TR = 488 ms; TE 1 = 4.49 ms; TE 2 = 6.95 ms) and a high-resolution structural scan (T1-weighted MPRAGE sequence; 1 x 1 x 1.2 mm) was acquired. Stimulus presentation was implemented by an MR-compatible LCD screen (NordicNeuroLab, Bergen, Norway) with a refresh rate of 60 Hz and a resolution of 1024 x 768 pixels.*

Experiment 3.
A Siemens Magnetom TRIO 3-Tesla scanner (Siemens AG, Erlangen, Germany) equipped with an 8-channel head-coil was used for functional and anatomical image acquisition. The BOLD signal was acquired with a T2-weighted gradient echo-planar imaging (EPI) sequence (TR = 2,250 ms; TE = 30 ms; Flip angle = 90°; 64 x 64 matrix; FoV = 210 mm). Thirty-six descending axial slices with a slice thickness of 3 mm and a slice gap of 0.3 mm were acquired within the TR. In addition, for each participant a gradient echo field map (TR = 650 ms; TE 1 = 4.89 ms; TE 2 = 7.35 ms) and a high-resolution structural scan (T1-weighted MPRAGE sequence; Experiment 3: 1 x 1 x 1 mm) was acquired. Stimulus presentation was implemented by a Sanyo PLC-XP41-projector (SANYO Electric Co., Osaka City, Japan) with a refresh rate of 60 Hz and a resolution of 1024 x 768 pixels.*"

#Issue 3:

I think the Discussion part dwells too much upon technical sides to give sufficient room to description for providing broad view of modelling (except Figure 7). Authors slightly touched upon the schema of framework only in the last paragraph playing the role of conclusion.

There is no paragraph where the authors should have mentioned limitations of the current study and clarified future perspective. The authors succeeded indeed in taking advantage of information theoretical concept of entropy, which allowed us to grasp the non-linearity of brain activation pattern for responses to orthographically legal pseudo words and orthographically anomalous (anormal) consonant strings. However, it would be worthwhile to reconfirm the effectiveness of this modeling by using the other ways of creating different types of pseudo words, such as less

noticeable misspelling with inversion of letters or random sequences of vowel as well as consonant letters. Readers might be interested in how various types of pseudo words left untouched will be placed within the graded continuum of this non-linearity.

Response:

We now added multiple paragraphs to the Discussion that are less technical. The very short Discussion resulted from an attempt to limit the length of this extensive manuscript, but we understand from reviewer comments that more Discussion should be devoted to conceptual issues. New sections of the Discussion focus on Dyslexia, model sprecificities, lvOT location, limitations and outlook.

The reviewer raises a question concerning different types of pseudowords than those used in our work so far. We think this is a fascinating question and a very plausible road for future work within the framework laid out here, but given the complexity of the manuscript, we did not attempt to include further empirical work.

While targeting a slightly different question, we nevertheless felt motivated by this reviewer comment to explore the stability of the LCM model against variation in the specific selection of stimuli (in terms of set size and specific items). To investigate the issue of how different sets of pseudowords influence the LCM simulations, we opted to estimate the internal stability of the model by randomly selecting subsets of different size (20 repetitions per set size) from the complete set of orthographic items that has been the basis of the LCM (see Figure 1a for the distributions of the complete set). Please note that our model formulation includes pseudowords that have been constructed with only subtle changes from the original words (i.e., one vocal change) and consonant strings, including more drastic changes. The recalculation of the LCM entropy with subsets of the orthographic stimuli indicated that with already 7% of all letter strings (N = 654 Words and nonwords), the correlation of the subset entropy and the entropy based on the entire set was above .9 (see Figure). This indicates that the entropy parameter calculated within the LCM framework is already stable with only a fraction of the entire dataset. We added this figure and a description of this result into the supplement, and refer to it from the figure capture of Figure 1 of the main text. Please find the figure below:

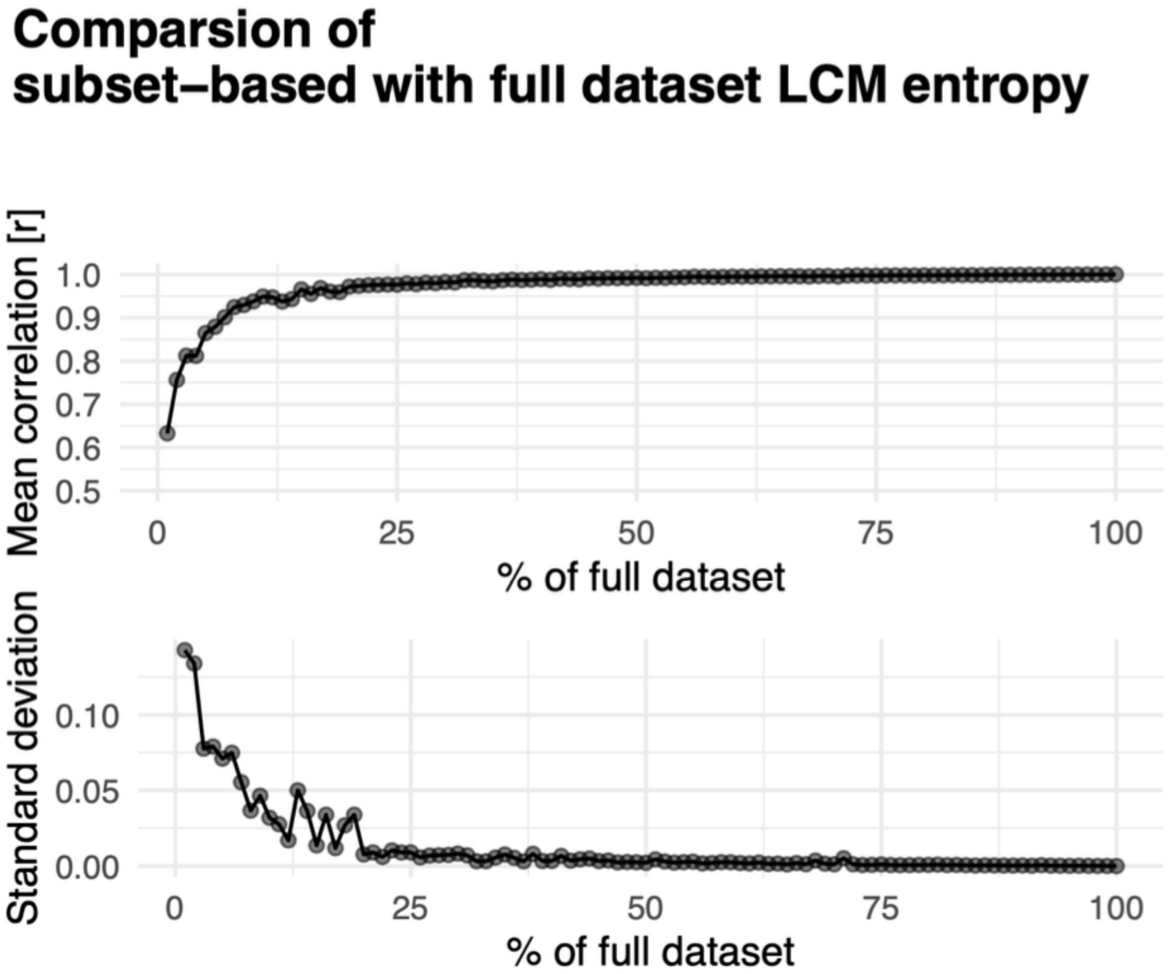

Supplementary figure 7. Correlation of the LCM calculated entropy from subsets of the stimuli with the full set of the stimuli. Upper section includes the mean correlation over twenty entropy correlations based on different randomly drawn sets of orthographic stimuli. Note, the proportion of words, pseudowords and consonant clusters was always the same. Lower section shows the standard deviation across the twenty calculations. Data is shown in 1% steps.

#Issue 4:

Things that I worry in addition is that although the ROIs of this paper should be in and around the visual word form area, no cluster peak was found in fusiform gyrus (we didn't find this region name in the text), and instead the activation was elicited rather in dorsal areas such as precuneus according to Supplementary Figure 3 for the fMRI study 1, This inconsistency might have prevented us from conceiving some detailed and concrete functional anatomy and networks of LCM except for the sketchy framework of Figure 7, I think that the authors might want to use the term of lvOT with reservations that it can include peripheral areas as a word in a broader sense.

<u>Response</u>:

We think that by using the label lvOT, we are consistent with many other groups in the field. This choice was made to avoid using the functional label of 'visual word form area', as this label is also closely

attached to a specific hypothesis that has been tested as an alternative model. Detailed anatomical descriptions of activation effects can be found in Table 1. Here, the LCM effect in fMRI Experiment 1 is explicitly attributed also to the fusiform gyrus by the automatic labeling system we have used (Cluster 1). As Figure 3 and 4 show, the LCM effect in Experiment 3 is in the direct vicinity of the cluster found in Experiment 1, but indeed not extending into FG, which is reflected in Table 1 (Cluster 3). Given that the peak coordinates of these two clusters are at a distance of at most 1cm in all three directions, we would attribute this to normal variation also observed across other studies in the field. We now add a more detailed discussion concerning the exact location of our findings in relation to results from the relevant literature (see below and at p. 29).

Please also note that we erroneously labeled the activation in the cuneus (shown in Suppl. Figure 3) as pre-cuneus. This error was corrected in the revised manuscript. Thanks for pointing it out.

"*At its core, the LCM-simulated activation reflects the difficulty of the process of lexical categorization based on the word-likeness of the orthographic input. It is easy to identify a letter string as a meaningful word when the word-likeness (i.e., orthographic familiarity) is high. It is also easy to categorize a letter string as meaningless when early visual processing stages identify it as unfamiliar given what we have learned, i.e., the lexicon of words we have acquired so far. Both describe efficient cases of lexical categorization of letter strings, resulting in fast responses. At intermediate levels of word-likeness, however, lexical categorization difficulty is high. The LCM postulates that this results from similar orthographic-perceptual familiarity of words and non-words fed into (word-sensitive subregions of) lvOT from lower levels, making a more detailed analysis of the orthographic input necessary. In the LCM implementation, this is accounted for by the partly overlapping distributions of word-likeness (OLD20) of words and non-words. The additional processing required by letter strings with intermediate word-likeness results in increased lvOT activation and slower responses, a proposal consistent with seminal behavioral work which proposed spelling information as a potential source aiding categorization when word-likeness information is insufficient (Balota & Chumbley, 1984). This, in turn, is compatible with our observation from fMRI Study 3, in which we used stimulus materials that were particularly difficult to categorize (e.g., Consonant strings with a high word-likeness). In this experiment, we observed that a parietal network that likely represents a dorsal sensorimotor interface connecting auditory-phonological with articulatory representations (Hickok & Poeppel, 2007) co-activates with lvOT when lexical integration difficulty increases. This parietal co-activation, interestingly, also relates our results to the recent proposal of a posterior/anterior dissociation within the word-selective ventral occipito-temporal cortex (Lerma-Usabiaga et al., 2018; White et al., 2019): Whereas posterior lvOT (also referred to as VWFA-1 by (Lerma-Usabiaga et al., 2018; White et al., 2019)) has been characterized as implementing pre-lexical processing during visual word recognition and was shown to be structurally connected to the parietal cortex (Caffarra et al., 2021; Lerma-Usabiaga et al., 2018), the anterior lvOT (VWFA-2) shows selectivity to lexical stimulus properties (Lerma-Usabiaga et al., 2018; White et al., 2019). The LCM-correlate identified in lvOT in the present study (Study 1: y = -64; Study 3: y = -75) is located close to the posterior lvOT subregion (Ref. (Lerma-Usabiaga et al., 2018): y < -63; Ref. (White et al., 2019): y ~ -69), suggesting a primarily prelexical nature of the categorization process implemented in the LCM. A proposal that is in line with the functional profiles of reading-related brain regions identified with EcOG electrodes (Lochy et al., 2018; Woolnough et al., 2020). Note, however, that these group-level effects also need to be interpreted in light of substantial inter-individual differences in functional localization of word-selective activity (e.g., (Caffarra et al., 2021)), so that the association between LCM effects and posterior lvOT/VWFA-2 should be replicated based on individual functional localizers.*" p.34

#Issue 5:

I enumerate here some trivial flaws here. p36. "a more optimal word-likeness estimate (i.e., OLD20) evident from previous comparisons of different word-likeness meases based on behavioral data (26)." What do they mean by "meases"?

Response:

Thanks for pointing this typo out. We corrected this to 'measures'.

Reviewer #4:

In this manuscript, the authors proposed that a lexical categorization model (LCM) reflects the processing in the left ventral occipito-temporal cortex (lvOT), and could account for different activation patterns in the literature. The authors performed an impressive amount of work, examining the LCM model in 3 different fMRI experiments and in behavioral training. The results give support that some activation clusters around lvOT performs similar computations as the LCM model predicted.

Response: Thank you for the nice words.

#Issue 1:

I have two comments related to each other:

1. *Conceptually, the authors attempted to use one model to explain a large list of possible discrepancies in the literature, which may be a bit ambitious. Because the discrepancy of the results in the literature is not necessarily found for exactly the same brain region. Regions nearby (e.g. posterior or anterior to the lvOT ROIs defined by the authors) may have different processing properties, see Lochy et al. (2018) for example. Lochy, A., Jacques, C., Maillard, L., Colnat-Coulbois, S., Rossion, B., & Jonas, J. (2018). Selective visual representation of letters and words in the left ventral occipito-temporal cortex with intracerebral recordings. Proceedings of the National Academy of Sciences, 115(32), E7595-E7604.*

Response:

We agree with the reviewer that there is variability in the localization of the effects reported across published studies, even though all of them have been attributed by the original authors to the visual word form area or word-sensitive lvOT. The cited paper by Lochy et al. is exciting. It seems to add to a growing literature discussing potential subdivisions within lvOT, responsive to different components of the word recognition process (see also the work by Lerma-Usabiaga et al., 2018, and White et al., 2019 proposing a functional dissociation between posterior/prelexical and anterior/lexical and semantic subregions). Concerning the posterior/anterior distinction proposed by these studies, our results seem to associate the lexical categorization process with the more posterior, pre-lexical processes. However, Lochy et al.'s results show inter-mingled lexical and pre-lexical functions roughly in the more posterior

location discussed by Lerma-Usabiaga and White. Ultimately, it would be interesting to apply a model-based approach as reported in our paper to intracranial data like that reported in Lochy et al. In light of the present comment and comments by reviewers 1 and 2, we have expanded the discussion of potential functional subdivisions within the word-sensitive cortex. Please refer to our response to Reviewer 1, Issue 2, where we also refer to the study by Lochy et al. cited here.

As the reviewer points out – it does not necessarily have to be the case that one model can account for all effects reported in the previous literature - particularly not if different effects stem from different brain regions. However, it should also be noted that there are also descriptively similar effects in the previous literature that varied in their specific peak-coordinates between studies (e.g., Caffarra et al., 2021). For that reason, we tend to believe that not all variations in localization can be considered robust and meaningful. Here, we would like to stress that we have proposed a computationally explicit model that can account for a wide range of effects – and its model parameter is mapped onto a single location in more than one dataset in our study. Given this result, we see at present no need to integrate anatomical variability into our considerations. However, the model-based approach introduced here bears the potential that future models can propose more fine-grained modeling of subfield specific effects.

"At its core, the LCM-simulated activation reflects the difficulty of the process of lexical categorization based on the word-likeness of the orthographic input. It is easy to identify a letter string as a meaningful word when the word-likeness (i.e., orthographic familiarity) is high. It is also easy to categorize a letter string as meaningless when early visual processing stages identify it as unfamiliar given what we have learned, i.e., the lexicon of words we have acquired so far. Both describe efficient cases of lexical categorization of letter strings, resulting in fast responses. At intermediate levels of word-likeness, however, lexical categorization difficulty is high. The LCM postulates that this results from similar orthographic-perceptual familiarity of words and non-words fed into (word-sensitive subregions of) lvOT from lower levels, making a more detailed analysis of the orthographic input necessary. In the LCM implementation, this is accounted for by the partly overlapping distributions of word-likeness (OLD20) of words and non-words. The additional processing required by letter strings with intermediate word-likeness results in increased lvOT activation and slower responses, a proposal consistent with seminal behavioral work which proposed spelling information as a potential source aiding categorization when word-likeness information is insufficient (Balota & Chumbley, 1984). This, in turn, is compatible with our observation from fMRI Study 3, in which we used stimulus materials that were particularly difficult to categorize (e.g., Consonant strings with a high word-likeness). In this experiment, we observed that a parietal network that likely represents a dorsal sensorimotor interface connecting auditory-phonological with articulatory representations (Hickok & Poeppel, 2007) co-activates with lvOT when lexical integration difficulty increases. This parietal co-activation, interestingly, also relates our results to the recent proposal of a posterior/anterior dissociation within the word-selective ventral occipito-temporal cortex (Lerma-Usabiaga et al., 2018; White et al., 2019): Whereas posterior lvOT (also referred to as VWFA-1 by (Lerma-Usabiaga et al., 2018; White et al., 2019)) has been characterized as implementing pre-lexical processing during visual word recognition and was shown to be structurally connected to the parietal cortex (Caffarra et al., 2021; Lerma-Usabiaga et al., 2018), the anterior lvOT (VWFA-2) shows selectivity to lexical stimulus properties (Lerma-Usabiaga et al., 2018; White et al., 2019). The LCM-correlate identified in lvOT in the present study (Study 1: $y = -64$; Study 3: $y = -75$) is located close to the posterior lvOT subregion (Ref. (Lerma-Usabiaga et al., 2018): $y < -63$; Ref. (White et al., 2019): $y \sim -69$), suggesting a primarily prelexical nature of the categorization process implemented in the LCM. A proposal that is in line with the functional profiles of reading-related brain regions identified with EcOG electrodes (Lochy et al., 2018; Woolnough et al., 2020). Note, however, that these group-level effects also need to be interpreted in light of substantial inter-individual differences in functional localization of word-selective activity (e.g., (Caffarra et al., 2021)),

so that the association between LCM effects and posterior lvOT/VWFA-2 should be replicated based on individual functional localizers. " p.34

2. The authors used the LCM simulation as the predictor, and found activation clusters in lvOT. This result by itself is interesting. But then the authors used these activation clusters as ROIs, and compared different models within this ROI, this creates circularity and a certain bias towards the LCM model (Figure 5 m and o), since the voxels were selected by this model. This problem is present in study 1 and 3, not in study 2 (independent voxel selection, used the voxels from study 1). If the authors use the E&E and IA models as single predictors and select the activation clusters as ROIs, the activation clusters may appear in different locations, and would be biased towards the E&E and IA models.

It would be useful to see the activation for the E&E and IA models, and see whether they overlap in the lvOT clusters found by the LCM model. The overlapping voxels could be then used for model evaluation, which does not have a big bias towards one model. If they do not result in overlapping voxels at the group level, the authors could examine the overlap and define ROIs at the individual level, although it is a lot of work.

Another way to avoid circularity (for study 3) is to use half of the data to define the ROI, and the other half to evaluate the models.

Kriegeskorte, N., Simmons, W. K., Bellgowan, P. S., & Baker, C. I. (2009). Circular analysis in systems neuroscience: the dangers of double dipping. Nature neuroscience, 12(5), 535-540.

Response:

We agree with the reviewer and also want to point out that exactly the same criticism was raised by Reviewer 1. In response to this, we have now also conducted unbiased whole-brain analyses of the third fMRI experiment (Study 3), based on the simulations of the IA and the E&E models, respectively, as an extension to Figure 3. We chose this alternative as we did not implement independent localizer scans in the study, and we used the data from Study 3 for this analysis, as this study used stimulus material specifically chosen to distinguish between the cognitive processes implemented in the most promising models (which are capable of simulating most effects present in the literature). As suggested by the reviewer, if an alternative model describes a computation implemented elsewhere in vOT, this analysis should identify it. As the correlations among the parameters from the different models are low, we would expect a neuroanatomical differentiation between the three models. The correlation analysis of model simulations and brain activation was implemented in the same way as the LCM-based analysis already presented (methods on p. 42).

From this analysis, the critical finding was that no voxels of the vOT correlated significantly with the IA and E&E model parameters (see Supplentary Fig. 3 and below). Also, the results of these two analyses identified different brain areas as correlating with the parameters from the different models: The IA model correlates with left frontal and bilateral temporal regions (Figure, upper row). In contrast, the E&E model with left peri-central regions. For comparison, the analogous analysis for the LCM model is presented in Figure 3&4 and shows robust correlation effects with voxels in lvOT.

The reviewer also discusses the likelihood that a single model can characterize the cognitive processes of all vOT. We agree with the reviewer that other factors and processes beyond those captured by the LCM model may contribute to the processes that lvOT contributes to word recognition. Given

the currently available data and models, we think that the additional results add evidence to our conclusion that the LCM, out of the currently available models, at present provides the best quantitative description of lvOT activation in response to words and non-words. The model fit is not perfect, so there might be different explanations with better results, but the LCM-based design for stimuli used in the fMRI experiment 3 and the LCM motivated training study are two pieces of evidence that indicate causality. In addition, other factors, besides computation, are likely to be responsible for variation here.

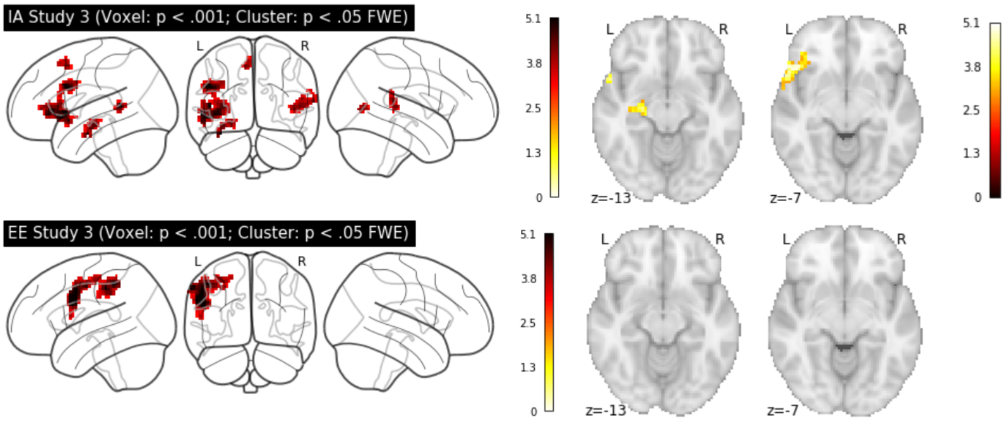

Supplemental figure 3. IA (upper row) and E&E model evaluation (bottom row): fMRI whole-brain analyses. Significant correlation results between BOLD signals and parameters from the IA and E&E simulations modeled as a single, continuous predictor in Study 3. Thresholds for all whole-brain analyses: voxel-level: $p < .001$ uncorrected (cluster-forming); cluster level: $p < .05$ family-wise error corrected. No other regions than those displayed were significant.

Minor comments:

Somehow the dots in Figure 2 (de) and Figure 5 (pq) were missing in the pdf file of the manuscript.

Response:

Thanks for pointing this out. This may have happened during the upload into the submission system, as the dots are present in the bioRxiv preprint. We will specifically check this upon re-submission.

Supplementary Figure 3: the second cluster appears to be cuneus, rather than precuneus.

Response:

Thank you for pointing this out. This is absolutely correct and was corrected.
