# Supplementary material for: The lexical categorization model: A computational model of left ventral occipito-temporal cortex activation in visual word recognition
Source: PLoS Comput Biol. 2022 Jun 9;18(6):e1009995. doi: 10.1371/journal.pcbi.1009995 (PMC9182256; doi:10.1371/journal.pcbi.1009995)
Supplement: Attachment — Submitted filename: Response.docx [file pcbi.1009995.s010.docx]

2022/1/25

I think that the initial request I had made for revision has been entirely and appropriately accommodated by the authors. I accept this article. Although this response seems a little short, I have decided to send you my positive answer after meticulously checking all the works that they made for improving the original version of this article.

Thank you very much.

Hiroyuki Akama
