# Supplementary material for: The lexical categorization model: A computational model of left ventral occipito-temporal cortex activation in visual word recognition
Source: PLoS Comput Biol. 2022 Jun 9;18(6):e1009995. doi: 10.1371/journal.pcbi.1009995 (PMC9182256; doi:10.1371/journal.pcbi.1009995)
Supplement: Peer review history

# Peer review: Decision Letter 0

**DOI:** 10.1371/journal.pcbi.1009995.r001

**Authors:** Wolfgang Einhäuser, Megan A. K. Peters

29 Jul 2021

Dear Dr. Gagl,

Thank you very much for submitting your manuscript "The lexical categorization model: A computational model of left ventral occipito-temporal cortex activation in visual word recognition" for consideration at PLOS Computational Biology.

As with all papers reviewed by the journal, your manuscript was reviewed by members of the editorial board and by several independent reviewers. In light of the reviews (below this email), we would like to invite the resubmission of a significantly-revised version that takes into account the reviewers' comments.

All four reviewers found the results intriguing. Reviewers 1 and 4 especially noted some technical and conceptual issues that should be addressed in a revision. I encourage you to consider these suggestions carefully in revising your manuscript, and also to note that several reviewers commented also about the availability of the data and code. Additionally, several reviewers suggested some organizational changes that I suggest you consider.

We cannot make any decision about publication until we have seen the revised manuscript and your response to the reviewers' comments. Your revised manuscript is also likely to be sent to reviewers for further evaluation.

When you are ready to resubmit, please upload the following:

[1] A letter containing a detailed list of your responses to the review comments and a description of the changes you have made in the manuscript. Please note while forming your response, if your article is accepted, you may have the opportunity to make the peer review history publicly available. The record will include editor decision letters (with reviews) and your responses to reviewer comments. If eligible, we will contact you to opt in or out.

[2] Two versions of the revised manuscript: one with either highlights or tracked changes denoting where the text has been changed; the other a clean version (uploaded as the manuscript file).

Important additional instructions are given below your reviewer comments.

Please prepare and submit your revised manuscript within 60 days. If you anticipate any delay, please let us know the expected resubmission date by replying to this email. Please note that revised manuscripts received after the 60-day due date may require evaluation and peer review similar to newly submitted manuscripts.

Thank you again for your submission. We hope that our editorial process has been constructive so far, and we welcome your feedback at any time. Please don't hesitate to contact us if you have any questions or comments.

Sincerely,

Megan A. K. Peters, Ph.D.

Associate Editor

PLOS Computational Biology

Wolfgang Einhäuser

Deputy Editor

PLOS Computational Biology

***********************

Reviewer's Responses to Questions

**Comments to the Authors:**

**Please note here if the review is uploaded as an attachment.**

Reviewer #1: Gagl and colleagues present an elegant and simple computational model to explain response properties in regions of ventral occipitotemporal cortex that respond to words. Their “Lexical Categorization Model” is intuitively simple while also having great explanatory power. It is based on the concept of “word likeness” which they instantiate in a simple and well-defined computation. Gagl and colleagues take a field that generally relies on verbal descriptions of effects and builds the first fully computable model of lexical processing in VOTC. I believe that this paper defines a solid foundation for future work to build on. I would also like to commend the authors for making their simulation code and model fitting code publicly available: allowing other scientists to work with this model will amplify the impact of this work. However, there is one major technical issue that needs to be addressed and there are a series of theoretical points that I think require further thought and attention.

Sincerely,

Jason Yeatman

Technical issue:

Was a whole-brain analysis conducted for all the different models? The results suggest that they searched for voxels that fit the LCM model and then model comparisons were conducted on this region. This analysis would bias results in favor of the LCM model - this is not a valid approach to model comparison. To perform an unbiased model comparison it would be important to establish an a priori localizer to define a region that would then be compared across the different models. Alternatively, a wholebrain analysis could be run for each model, and the results could be reported (but not directly compared). It is likely that there isn’t a single computation performed in VOTC and that different models might fit better for different regions. This alternative seems more likely than a single model that characterizes all of VOTC.

Conceptual:

There is one major conceptual clarification that should be made up front. The goal of the LCM is to explain the processing of orthographic stimuli in VOTC. This is very different from a general purpose model to explain the wide array of response properties reported in VOTC. For example, the response to “false fonts” or foreign character strings or line drawings is nearly as high as the response to words and there is still a moderate response to checker boards, faces, objects and other visual stimuli in VOTC. There are also neighboring regions that prefer these other stimuli. The LCM model only explains the incremental differences in response to different orthographic stimuli and is not computable based on the wide range of other visual stimuli that have been used to probe VOTC responses. This is a major distinction between the LCM and the fully computable model of VOTC developed by Kay and Yeatman 2017. In contrast, the sequence of models developed by Kay and Yeatman 2017 sought to characterize the general response pattern of different category selective VOTC regions but did not address the effects of different orthographic and lexical properties. I think it would be interesting to consider whether these two models might represent different stages of processing in word-selective cortex. For example, the template model from Kay and Yeatman might be implemented in an earlier (e.g. VWFA-1 or OWFA) word-selective region that feeds into a later (e.g. VWFA-2) word-selective region that computes the LCM. Discussing these different approaches would be helpful and, at the very least, clearly defining the goal of modeling response differences to orthographic stimuli as opposed to a fully-computable, image-based model would be a useful distinction.

fMRI study 1 is a nice test of the model. However, a distinction should be made between a whole-brain analysis of data in template space and a model fit to responses from individually defined VWFA ROIs. The location of the VWFA varies dramatically across participants and the reported coordinates vary dramatically across studies. For example, it has become clear that there are, in fact, a sequence of spatially distinct word-selective regions with different functions: individual localizers are essential to accurately distinguish these regions. This is not a problem for the presented analyses per se, but should be considered in the interpretation. For example, see here the dramatic variability in reported “VWFA” coordinates across the literature (https://osf.io/hv3y2/) and see White et al. 2019 for a detailed, model-based characterization of computational differences in VWFA-1 and VWFA-2: White, Alex L., John Palmer, Geoffrey M. Boynton, and Jason D. Yeatman. 2019. “Parallel Spatial Channels Converge at a Bottleneck in Anterior Word-Selective Cortex.” Proceedings of the National Academy of Sciences of the United States of America 116 (20): 10087–96.

In line with the above comment of anatomical precision, sentences like “LCM provides a fair characterization of lvOT BOLD activation patterns during visual word recognition and that it predicts lvOT activity better than other current models of lvOT function“, should be revised. The wholebrain analysis shows that there are some voxels for which this statement is true. But the statement is not true about all of lvOT nor is it true about specific sub-regions of word-selective cortex for which the model does not explain certain effects. To be concrete, sentences like “In sum, empirical data from three fMRI studies support the proposal that lvOT implements a lexical categorization process that operates upon an estimate of word-likeness computed by hierarchically preceding brain regions.” Should be revised to say something like “In sum, empirical data from three fMRI studies support the proposal that THERE IS A REGION IN lvOT THAT implements a lexical categorization process that operates upon an estimate of word-likeness computed by hierarchically preceding brain regions.”

Similarly, the first sentence of the discussion should not label this region the visual word form area without performing a localizer in individual brains. “In the vicinity of the visual word form area” would be a fair statement.

Fmri Study 2 was a clever manipulation with clear results. But, in line with my previous point, the claims should be made specifically about the region identified in the above group average analysis not about VOTC in general or the VWFA specifically.

A couple general points about references:

Binder and colleagues showed similar findings to reference 12 (Vinckier) and would be a good citation to add about word similarity. I believe this was the first paper to make the referenced pointBinder, J. R., D. A. Medler, C. F. Westbury, E. Liebenthal, and L. Buchanan. 2006. “Tuning of the Human Left Fusiform Gyrus to Sublexical Orthographic Structure.” NeuroImage 33 (2): 739–48.

The paper doesn’t confront the broader work on computations performed in ventral occipitotemporal cortex. Even though there has not been much modeling work done for word recognition, there is a wealth of work developing computational models of the response properties in visual cortex. There are other papers that propose models of VOTC that address the response to a broad range of visual stimuli (without a specific focus on the differences between different letter combinations). A comprehensive review of this literature is not necessary. But acknowledging how the present model fits within the broader literature would make this work broadly influential. For example, Doris Tsao’s lab just published a paper in Nature presenting a new “object space” model. Our previous work developed a template model with top-down modulation from the IPS. Srihasam and colleagues posited a general architecture that predicts where word-selective regions should emerge in VOTC. Citations below:

Bao, Pinglei, Liang She, Mason Mcgill, and Doris Y. Tsao. 2020. “A Map of Object Space in Primate Inferotemporal Cortex.” Nature, no. January 2019. https://doi.org/10.1038/s41586-020-2350-5.

Kay, Kendrick N., and Jason D. Yeatman. 2017. “Bottom-up and Top-down Computations in Word- and Face-Selective Cortex.” eLife 6 (February). https://doi.org/10.7554/eLife.22341.

Srihasam, Krishna, Justin L. Vincent, and Margaret S. Livingstone. 2014. “Novel Domain Formation Reveals Proto-Architecture in Inferotemporal Cortex.” Nature Neuroscience 17 (12): 1776–83.

Reviewer #2: I’m not specialized in practice on computational model and MRI data processing, but both the method and results sections are quite clear and comprehensive. The introduction might be improved by adding some modeling / method comparison to help the reader clearly identify the objectives and originality of the study.

In the introduction, the authors wrote that empirical data indicate that word similarity increases lvOT activation and word-familiarity decreases lvOT activation. It would be interesting to present data of morphological and both morpho-semantic and morpho-orthographic processing since word similarity is related to sub-lexical orthographic similarity (and also morpho-orthographic processing) and word similarity is related to word frequency (and also morpho-semantic processing).

Also, some papers using representational similarity analysis (but also other analyses) have showed differences in the degree of activation along the lvOT cortex – left ventral inferior temporal cortex which is dependent to the level of stimuli and processing (posterior part more low level and anterior part more high level). If relevant, a discussion would be interesting.

The discussion should be better organized, especially the first paragraph which should present the objectives and results summary. Overall, I find the discussion quite short about the perspectives of application of this study and limitations. How these findings can be used by researchers or future studies? Also, a discussion about how this finding can be compared with connexionist approach would be very interesting in a theoretical perspective.

Does this model is sensitive to (modulated by) the reading related skills (word reading and pseudoword decoding skills, at least) of participants that were included in the fMRI experiment?

Would the authors be more specific and detailed about the training experiment (Evaluation C)? As currently wrote it is very difficult to have a clear picture of the training content and procedure.

It would have been interesting to use Representational similarity analysis to model the fMRI data. I am not asking the authors to do it, but maybe it would be interesting to make some hypothesis on other type of analyses, especially because you are using correlation analysis (Figure3).

I understand it is not the topic of this study, but since the authors have started the introduction by presenting the limit of the studies conducted on lvOT, including the fact that this region is compromised in developmental reading disorders but that there is no agreed-upon mechanistic understanding of the specific processes lvOT contributes to visual word recognition, it would be interesting to provide a summary of this specific question, and also some hypotheses about reading disabilities.

Reviewer #3: In this article, taking advantage of non-linear simulation based on information theory, authors challenged to explore the specific processes that the lvOT contributes to visual word recognition. They succeeded in explaining in a convincing manner the reason of superficial contradiction why word-similarity or sub-lexical orthographic similarity increases lvOT activation, where word-familiarity in the sense of word frequency decreases lvOT activation. I can underscore the research strategy of this article for substantive approach to the core of evaluating the neural activity as a non-linear function of orthographic structure. It is quite interesting to see that their methodology was accomplished by inventing a sound and quite ingenious simulation methodology. I quite agree with the authors that they could capture very well by the concept of entropy the pattern of lexical categorization difficulty.

Yet, in spite of their unimpeachable line of reasoning, it cannot be helped saying that this paper made a more or less messy impression. I strongly recommend that the authors devise nomenclature for brief representation of each particular experiment or methodology instead of mere numbering such as "Study 1" or "Evaluation C," once these notations appear alone like symbols. Since the organization of this paper is too complicated, I am afraid that the readers will feel difficulties in referring to the definitions and the contents only by casting a glance at these notations.

The explanation about each of the three fMRI experiments should be more concise in the Methods section (too long). This section is too labyrinthic to easily return to the main thread from each ramification. For example, readers will be wondering why the scan settings (even head coils were disparate) were so different across these three, because the experimenters did not need to overly adjust the purposes of the experiments to the minimal capacity of scanning conditions. It would be better to put these details to the supplementary documents to avoid apparent complication,

I think the Discussion part dwells too much upon technical sides to give sufficient room to description for providing broad view of modelling (except Figure 7). Authors slightly touched upon the schema of framework only in the last paragraph playing the role of conclusion.

There is no paragraph where the authors should have mentioned limitations of the current study and clarified future perspective. The authors succeeded indeed in taking advantage of information theoretical concept of entropy, which allowed us to grasp the non-linearity of brain activation pattern for responses to orthographically legal pseudo　words and orthographically anomalous (anormal) consonant strings. However, it would be worthwhile to reconfirm the effectiveness of this modeling by using the other ways of creating different types of pseudo words, such as less noticeable misspelling with inversion of letters or random sequences of vowel as well as consonant letters. Readers might be interested in how various types of pseudo words left untouched will be placed within the graded continuum of this non-linearity.

Things that I worry in addition is that although the ROIs of this paper should be in and around the visual word form area, no cluster peak was found in fusiform gyrus (we didn't find this region name in the text), and instead the activation was elicited rather in dorsal areas such as precuneus according to Supplementary Figure 3 for the fMRI study 1, This inconsistency might have prevented us from conceiving some detailed and concrete functional anatomy and networks of LCM except for the sketchy framework of Figure 7, I think that the authors might want to use the term of lvOT with reservations that it can include peripheral areas as a word in a broader sense.

I enumerate here some trivial flaws here.

p36. "a more optimal word-likeness estimate (i.e., OLD20) evident from previous comparisons of different word-likeness meases based on behavioral data (26)." What do they mean by "meases"?

Reviewer #4: In this manuscript, the authors proposed that a lexical categorization model (LCM) reflects the processing in the left ventral occipito-temporal cortex (lvOT), and could account for different activation patterns in the literature. The authors performed an impressive amount of work, examining the LCM model in 3 different fMRI experiments and in behavioral training. The results give support that some activation clusters around lvOT performs similar computations as the LCM model predicted.

I have two comments related to each other:

1. Conceptually, the authors attempted to use one model to explain a large list of possible discrepancies in the literature, which may be a bit ambitious. Because the discrepancy of the results in the literature is not necessarily found for exactly the same brain region. Regions nearby (e.g. posterior or anterior to the lvOT ROIs defined by the authors) may have different processing properties, see Lochy et al. (2018) for example.

Lochy, A., Jacques, C., Maillard, L., Colnat-Coulbois, S., Rossion, B., & Jonas, J. (2018). Selective visual representation of letters and words in the left ventral occipito-temporal cortex with intracerebral recordings. Proceedings of the National Academy of Sciences, 115(32), E7595-E7604.

2. The authors used the LCM simulation as the predictor, and found activation clusters in lvOT. This result by itself is interesting. But then the authors used these activation clusters as ROIs, and compared different models within this ROI, this creates circularity and a certain bias towards the LCM model (Figure 5 m and o), since the voxels were selected by this model. This problem is present in study 1 and 3, not in study 2 (independent voxel selection, used the voxels from study 1). If the authors use the E&E and IA models as single predictors and select the activation clusters as ROIs, the activation clusters may appear in different locations, and would be biased towards the E&E and IA models.

It would be useful to see the activation for the E&E and IA models, and see whether they overlap in the lvOT clusters found by the LCM model. The overlapping voxels could be then used for model evaluation, which does not have a big bias towards one model. If they do not result in overlapping voxels at the group level, the authors could examine the overlap and define ROIs at the individual level, although it is a lot of work.

Another way to avoid circularity (for study 3) is to use half of the data to define the ROI, and the other half to evaluate the models.

Kriegeskorte, N., Simmons, W. K., Bellgowan, P. S., & Baker, C. I. (2009). Circular analysis in systems neuroscience: the dangers of double dipping. Nature neuroscience, 12(5), 535-540.

Minor comments:

Somehow the dots in Figure 2 (de) and Figure 5 (pq) were missing in the pdf file of the manuscript.

Supplementary Figure 3: the second cluster appears to be cuneus, rather than precuneus.

**********

**Have the authors made all data and (if applicable) computational code underlying the findings in their manuscript fully available?**

The PLOS Data policy requires authors to make all data and code underlying the findings described in their manuscript fully available without restriction, with rare exception (please refer to the Data Availability Statement in the manuscript PDF file). The data and code should be provided as part of the manuscript or its supporting information, or deposited to a public repository. For example, in addition to summary statistics, the data points behind means, medians and variance measures should be available. If there are restrictions on publicly sharing data or code —e.g. participant privacy or use of data from a third party—those must be specified.

Reviewer #1: Yes

Reviewer #2: Yes

Reviewer #3: **No: **The reviewer checked that behavioral data files are available here: https://bit.ly/2I8leM5. However, according to the authors, fMRI raw data will not be made available due to data security issues. They declared that after acceptance the repository will be moved to OSF.

Reviewer #4: None

**********

PLOS authors have the option to publish the peer review history of their article (what does this mean?). If published, this will include your full peer review and any attached files.

If you choose “no”, your identity will remain anonymous but your review may still be made public.

**Do you want your identity to be public for this peer review?** For information about this choice, including consent withdrawal, please see our Privacy Policy.

Reviewer #1: **Yes: **Jason D. Yeatman

Reviewer #2: No

Reviewer #3: No

Reviewer #4: No

Figure Files:

While revising your submission, please upload your figure files to the Preflight Analysis and Conversion Engine (PACE) digital diagnostic tool, https://pacev2.apexcovantage.com. PACE helps ensure that figures meet PLOS requirements. To use PACE, you must first register as a user. Then, login and navigate to the UPLOAD tab, where you will find detailed instructions on how to use the tool. If you encounter any issues or have any questions when using PACE, please email us at figures@plos.org.

Data Requirements:

Please note that, as a condition of publication, PLOS' data policy requires that you make available all data used to draw the conclusions outlined in your manuscript. Data must be deposited in an appropriate repository, included within the body of the manuscript, or uploaded as supporting information. This includes all numerical values that were used to generate graphs, histograms etc.. For an example in PLOS Biology see here: http://www.plosbiology.org/article/info%3Adoi%2F10.1371%2Fjournal.pbio.1001908#s5.

Reproducibility:

To enhance the reproducibility of your results, we recommend that you deposit your laboratory protocols in protocols.io, where a protocol can be assigned its own identifier (DOI) such that it can be cited independently in the future. Additionally, PLOS ONE offers an option to publish peer-reviewed clinical study protocols. Read more information on sharing protocols at https://plos.org/protocols?utm_medium=editorial-email&utm_source=authorletters&utm_campaign=protocols

# Author comment: Author response to Decision Letter 0

**DOI:** 10.1371/journal.pcbi.1009995.r002

10 Jan 2022

Attachment Submitted filename: Response-letter-PLOS_compBio_R1.pdf Click here for additional data file.

# Peer review: Decision Letter 1

**DOI:** 10.1371/journal.pcbi.1009995.r003

**Authors:** Wolfgang Einhäuser, Megan A. K. Peters

7 Mar 2022

Dear Dr. Gagl,

We are pleased to inform you that your manuscript 'The lexical categorization model: A computational model of left ventral occipito-temporal cortex activation in visual word recognition' has been provisionally accepted for publication in PLOS Computational Biology.

Before your manuscript can be formally accepted you will need to complete some formatting changes, which you will receive in a follow up email. A member of our team will be in touch with a set of requests.

Please note that your manuscript will not be scheduled for publication until you have made the required changes, so a swift response is appreciated.

IMPORTANT: The editorial review process is now complete. PLOS will only permit corrections to spelling, formatting or significant scientific errors from this point onwards. Requests for major changes, or any which affect the scientific understanding of your work, will cause delays to the publication date of your manuscript.

Should you, your institution's press office or the journal office choose to press release your paper, you will automatically be opted out of early publication. We ask that you notify us now if you or your institution is planning to press release the article. All press must be co-ordinated with PLOS.

Thank you again for supporting Open Access publishing; we are looking forward to publishing your work in PLOS Computational Biology. 

Best regards,

Megan A. K. Peters, Ph.D.

Associate Editor

PLOS Computational Biology

Wolfgang Einhäuser

Deputy Editor

PLOS Computational Biology

***********************************************************

Reviewer's Responses to Questions

**Comments to the Authors:**

**Please note here if the review is uploaded as an attachment.**

Reviewer #1: The authors have done a comprehensive job addressing all my comments and this is a strong paper that will contribute substantially to the literature.

Sincerely,

Jason Yeatman

Reviewer #3: Thank you very much for your great efforts to revise your important article.

Reviewer #4: The authors have addressed most of the points in my comments.

I agree with Reviewer #1 on the point of the large inter-individual variability (I also suggested for examining the effect of models in individual participants to account for that). The authors seem to have touched on this point lightly, by one sentence in the discussion, citing ref 41. If the authors are willing to improve the manuscript a bit further, the authors could expand the discussion a bit more, based on ref 21 (Glezer & Riesenhuber, 2013, showing the word-selectivity effect being washed out in ROIs defined at the group level, or from coordinates in the literature), which is currently cited on a different occasion.

**********

**Have the authors made all data and (if applicable) computational code underlying the findings in their manuscript fully available?**

The PLOS Data policy requires authors to make all data and code underlying the findings described in their manuscript fully available without restriction, with rare exception (please refer to the Data Availability Statement in the manuscript PDF file). The data and code should be provided as part of the manuscript or its supporting information, or deposited to a public repository. For example, in addition to summary statistics, the data points behind means, medians and variance measures should be available. If there are restrictions on publicly sharing data or code —e.g. participant privacy or use of data from a third party—those must be specified.

Reviewer #1: None

Reviewer #3: Yes

Reviewer #4: None

**********

PLOS authors have the option to publish the peer review history of their article (what does this mean?). If published, this will include your full peer review and any attached files.

If you choose “no”, your identity will remain anonymous but your review may still be made public.

**Do you want your identity to be public for this peer review?** For information about this choice, including consent withdrawal, please see our Privacy Policy.

Reviewer #1: **Yes: **Jason Yeatman

Reviewer #3: **Yes: **Hiroyuki Akama

Reviewer #4: No

Attachment Submitted filename: Response.docx Click here for additional data file.

# Editor report: Acceptance letter

**DOI:** 10.1371/journal.pcbi.1009995.r004

**Authors:** Wolfgang Einhäuser, Megan A. K. Peters

17 May 2022

PCOMPBIOL-D-21-00321R1 

The lexical categorization model: A computational model of left ventral occipito-temporal cortex activation in visual word recognition

Dear Dr Gagl,

I am pleased to inform you that your manuscript has been formally accepted for publication in PLOS Computational Biology. Your manuscript is now with our production department and you will be notified of the publication date in due course.

The corresponding author will soon be receiving a typeset proof for review, to ensure errors have not been introduced during production. Please review the PDF proof of your manuscript carefully, as this is the last chance to correct any errors. Please note that major changes, or those which affect the scientific understanding of the work, will likely cause delays to the publication date of your manuscript. 

Soon after your final files are uploaded, unless you have opted out, the early version of your manuscript will be published online. The date of the early version will be your article's publication date. The final article will be published to the same URL, and all versions of the paper will be accessible to readers.

Thank you again for supporting PLOS Computational Biology and open-access publishing. We are looking forward to publishing your work! 

With kind regards,

Anita Estes

PLOS Computational Biology | Carlyle House, Carlyle Road, Cambridge CB4 3DN | United Kingdom ploscompbiol@plos.org | Phone +44 (0) 1223-442824 | ploscompbiol.org | @PLOSCompBiol
